# The Interaction of Radio-Frequency Fields With Dielectric Materials at Macroscopic to Mesoscopic Scales

**DOI:** 10.6028/jres.117.001

**Published:** 2012-02-02

**Authors:** James Baker-Jarvis, Sung Kim

**Affiliations:** Electromagnetics Division, National Institute of Standards and Technology, Boulder, Colorado 80305

**Keywords:** dielectric, electromagnetic fields, loss factor, metamaterials, microwave, millimeter wave, nanoscale, permeability, permittivity, plasmon, polariton

## Abstract

The goal of this paper is to overview radio-frequency (RF) electromagnetic interactions with solid and liquid materials from the macroscale to the nanoscale. The overview is geared toward the general researcher. Because this area of research is vast, this paper concentrates on currently active research areas in the megahertz (MHz) through gigahertz (GHz) frequencies, and concentrates on dielectric response. The paper studies interaction mechanisms both from phenomenological and fundamental viewpoints. Relaxation, resonance, interface phenomena, plasmons, the concepts of permittivity and permeability, and relaxation times are summarized. Topics of current research interest, such as negative-index behavior, noise, plasmonic behavior, RF heating, nanoscale materials, wave cloaking, polaritonic surface waves, biomaterials, and other topics are overviewed. Relaxation, resonance, and related relaxation times are overviewed. The wavelength and material length scales required to define permittivity in materials is discussed.

## 1. Introduction

### 1.1 Background

In this paper we will overview electromagnetic interactions with solid and liquid dielectric and magnetic materials from the macroscale down to the nanoscale. We will concentrate our effort on radio-frequency (RF) waves that include microwaves (MW) and millimeter-waves (MMW), as shown in Table 1. Radio frequency waves encompass frequencies from 3 kHz to 300 GHz. Microwaves encompass frequencies from 300 MHz to 30 GHz. Extremely high-frequency waves (EHF) and millimeter waves range from 30 GHz to 300 GHz.

Many devices operate through the interaction of RF electromagnetic waves with materials. The characterization of the interface and interaction between fields and materials is a critical task in any electromagnetic (EM) device or measurement instrument development, from nanoscale to larger scales. Electromagnetic waves in the radio-frequency range have unique properties. These attributes include the ability to travel in guided-wave structures, the ability of antennas to launch waves that carry information over long distances, possess measurable phase and magnitude, the capability for imaging and memory storage, dielectric heating, and the ability to penetrate materials.

Some of the applications we will study are related to areas in microelectronics, bioelectromagnetics, homeland security, nanoscale and macroscale probing, magnetic memories, dielectric nondestructive sensing, radiometry, dielectric heating, and microwave-assisted chemistry. For nanoscale devices the RF wavelengths are much larger than the device. In many other applications the feature size may be comparable or larger than the wavelength of the applied field.

We will begin with an introduction of the interaction of fields with materials and then overview the basic notations and definitions of EM quantities, then progress into dielectric and magnetic response, definitions of permittivity and permeability, fields, relaxation times, surfaces waves, artificial materials, dielectric and magnetic heating, nanoscale interactions, and field fluctuations. The paper ends with an overview of biomaterials in EM fields and metrologic issues. Because this area is very broad, we limit our analysis to emphasize solid and liquid dielectrics over magnetic materials, higher frequencies over low frequencies, and classical over quantum-mechanical descriptions. Limited space will be used to overview electrostatic fields, radiative fields, and terahertz interactions. There is minimal discussion of EM interactions with nonlinear materials and gases.

### 1.2 Electromagnetic Interactions From the Microscale to Macroscale

In this section we want to briefly discuss electromagnetic interaction with materials on the microscale to the macroscale.

Matter is modeled as being composed of many uncharged and charged particles including for example, protons, electrons, and ions. On the other hand, the electromagnetic field is composed of photons. The internal electric field in a material is related to the sum of the fields from all of the charged particles plus any applied field. When particles such as biological molecules, cells, or inorganic materials are subjected to external electric fields, the molecules can respond in a number of ways. For example, a single charged particle will experience a force in an applied electric field. Also, in response to electric fields, the charges in a neutral many-body particle may separate to form induced dipole moments, which tend to align in the field; however this alignment is in competition with thermal effects. Particles that have permanent dipole moments will interact with applied dc or high-frequency fields. In an electric field, particles with permanent dipole-moments will tend to align due to the electrical torque, but in competition to thermal randomizing effects. When EM fields are applied to elongated particles with mobile charges, they tend to align in the field. If the field is nonuniform, the particle may experience dielec-trophoresis forces due to field gradients.

**Fig. 1:**
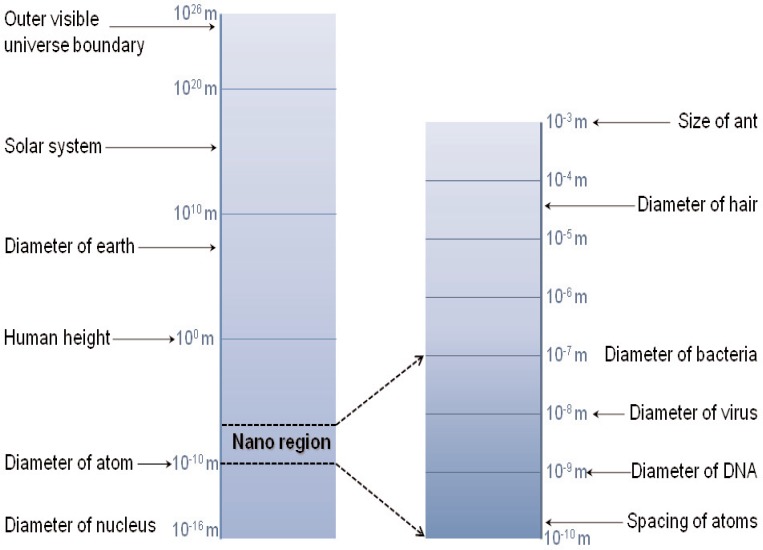
Scales of objects.

On the microscopic level we know that the electromagnetic field is modeled as a collection of photons [2]. In theory, the electromagnetic field interactions with matter may be modeled on a microscopic scale by solving Schrödinger’s equation, but generally other approximate approaches are used. At larger scales the interaction with materials is modeled by macroscopic Maxwell’s equations together with constitutive relations and boundary conditions. At a courser level of description, phenomenological and circuit models are commonly used. Typical scales of various objects are shown in Fig. 1. The mesoscopic scale is where classical analysis begins to be modified by quantum mechanics and is a particularly difficult area to model.

The interaction of the radiation field with atoms is described by quantum electrodynamics. From a quantum-mechanical viewpoint the radiation field is quantized, with the energy of a photon of angular frequency ω being *E* = *ħ*ω. Photons exhibit wave-duality and quantization. This quantization also occurs in mechanical behavior where lattice vibrational motion is quantized into phonons. Commonly, an atom is modeled as a harmonic oscillator that absorbs or emits photons. The field is also quantized, and each field mode is represented as a harmonic oscillator and the photon is the quantum particle.

The radiation field is usually assumed to contain a distribution of various photon frequencies. When the radiation field interacts with atoms at the appropriate frequency, there can be absorption or emission of photons. When an atom emits a photon, the energy of the atom decreases, but then the field energy increases. Rigorous studies of the interaction of the molecular field with the radiation field involve quantization of the radiation field by expressing the potential energy *V*(*r*) and vector potential **A**(**r**, *t*) in terms of creation and annihilation operators and using these fields in the Hamiltonian, which is then used in the Schrödinger equation to obtain the wavefunction (see, for example, [3]). The static electromagnetic field is sometimes modeled by virtual photons that can exist for the short periods allowed by the uncertainty principle. Photons can interact by depositing all their energy in photoelectric electron interactions, by Compton scattering processes, where they deposit only a portion of the energy together with a scattered photon, or by pair production. When a photon collides with an electron it deposits its kinetic energy into the surrounding matter as it moves through the material. Light scattering is a result of changes in the media caused by the incoming electromagnetic waves [4]. In Rayleigh elastic light scattering, the photons of the scattered incident light are used for imaging material features. Brillouin scattering is an inelastic collision that may form or annihilate quasiparticles such as phonons, plasmons, and magnons. Plasmons relate to plasma oscillations, often in metals, that mimic a particle and magnons are the quanta in spin waves. Brillouin scattering occurs when the frequency of the scattered light shifts in relation to the incident field. This energy shift relates to the energy of the interacting quasiparticles. Brillouin scattering can be used to probe mesoscopic properties such as elasticity. Raman scattering is an inelastic process similar to Brillouin scattering, but where the scattering is due to molecular or atomic-level transitions. Raman scattering can be used to probe chemical and molecular structure. Surface-enhanced Raman scattering (SERS) is due to enhancement of the EM field by surface-wave excitation [5].

Optically transparent materials such as glass have atoms with bound electrons whose absorption frequencies are not in the visible spectrum and, therefore, incident light is transmitted through the material. Metallic materials contain free electrons that have a distribution of resonant frequencies that either absorb incoming light or reflect it. Materials that are absorbing in one frequency band may be transparent in another band.

Polarization in atoms and molecules can be due to permanent electric moments or induced moments caused by the applied field, and spins or spin moments. The response of induced polarization is usually weaker than that of permanent polarization, because the typical radii of atoms are on the order of 0.1 nm. On application of a strong external electric field, the electron cloud will displace the bound electrons only about 10^−16^ m. This is a consequence of the fact that the atomic electric fields in the atom are very intense, approximately 10^11^ V/m. The splitting of spectral lines due to the interaction of electric fields with atoms and molecules is called the Stark effect. The Stark effect occurs when interaction of the electric-dipole moment of molecules interacts with an applied electric field that changes the potential energy and promotes rotation and atomic transitions. Because the rotation of the molecules depends on the frequency of the applied field, the Stark effect depends on both the frequency and field strength. The interaction of magnetic fields with molecular dipole moments is called the Zeeman effect. Both the Stark and Zeeman effects have fine-structure modifications that depend on the molecule’s angular momentum and spin. On a mesoscopic scale, the interactions are summarized in the Hamiltonian that contains the internal energy of the lattice, electric and magnetic dipole moments, and the applied fields.

In modeling EM interactions at macroscopic scales, a homogenization process is usually applied and the classical Maxwell field is treated as an average of the photon field. There also is a homogenization process that is used in deriving the macroscopic Maxwell equations from the microscopic Maxwell equations. The macroscopic Maxwell’s equations in materials are formed by averaging the microscopic equations over a unit cell. In this averaging procedure, the macroscopic charge and current densities, the magnetic field **H**, the magnetization **M**, the displacement field **D**, and the electric polarization field **P** are formed. At these scales, the molecule dipole moments are averaged over a unit cell to form continuous dielectric and magnetic polarizations **P** and **M**. The constitutive relations for the polarization and magnetization are used to define the permittivity and permeability. At macroscopic to mesoscopic scales the permittivity, permeability, refractive index, and impedance are used to model the response of materials to applied fields. We will discuss this in detail in Sec. 4.5. Quantities such as permittivity, permeability, refractive index, and wave impedance are not microscopic quantities, but are defined through an averaging procedure. This averaging works well when the wavelength is much larger than the size of the molecules or atoms and when there are a large number of molecules. In theoretical formulations for small scales and wavelengths near molecular dimensions, the dipole moment and polarizability tensor of atoms and molecules can be used rather than the permittivity or permeability. In some materials, such as magnetoelectric and chiral materials, there is a coupling between the electric and magnetic responses. In such cases the time-harmonic constitutive relations are $\tilde{B}(\omega )=\mu \tilde{H}(\omega )+\eta_{1}\tilde{E}(\omega )$ and $\tilde{D}(\omega )=\varepsilon \tilde{E}(\omega )+\eta_{2}\tilde{H}(\omega )$. In most materials the constitutive relations $\tilde{B}(\omega )=\mu_{0}(\tilde{M}(\omega )+\tilde{H}(\omega ))$ and $\tilde{D}(\omega )=\varepsilon_{0}\tilde{E}(\omega )+\tilde{P}(\omega )$ are used.

In any complex lossy system, energy is converted from one form to another, such as the transformation of EM energy to lattice kinetic energy and thermal energy through photon-phonon interactions. Some of the energy in the applied fields that interact with materials is transfered into thermal energy as infrared phonons. In a waveguide, there is a constant exchange of energy between the charge in the guiding conductors and the fields [6].

When the electromagnetic field interacts with material degrees of freedom, a collective response may be generated. The term polariton relates to bosonic quasi-particles resulting from the coupling of EM photons or waves with an electric or magnetic dipole-carrying excitation [4, 5]. The resonant and nonresonant coupling of EM fields in phonon scattering is mediated through the phonon-polariton transverse-wave quasi-particle. Phonon polaritons are formed from photons interacting with terahertz to optical phonons. Ensembles of electrons in metals form plasmas and high-frequency fields applied to these electron gases produce resonant quasi-particles, commonly called plasmons. Plasmons are a collective excitation of a group of electrons or ions that simultaneously oscillate in the field. An example of a plasmon is the resonant oscillation of free electrons in metals and semiconductors in response to an applied high-frequency field. Plasmons may also form at the interface of a dielectric and a metal and travel as a surface wave with most of the EM energy confined to the low-loss dielectric. A surface plasmon polariton is the coupling of a photon with surface plasmons. Whereas transverse plasmons can couple to an EM field directly, longitudinal plasmons couple to the EM field by secondary particle collisions. In the microwave and millimeter wave bands artificial structures can be machined in metallic surfaces to produce plasmons-like excitations due to geometry. Magnetic coupling is mediated through magnons and spin waves. A magnon is a quantum of a spin wave that travels through a spin lattice. A polaron is an excitation caused by a polarized electron traveling through a material together with the resultant polarization of adjacent dipoles and lattice distortion [4]. All of these effects are manifest at the mesoscale through macroscale in the constitutive relations and the resultant permittivity and permeability.

### 1.3 Responses to Applied RF Fields

If we immerse a specimen in an applied field and the response is recorded by a measurement device, the data obtained are usually in terms of a digital readout or a needle deflection indicating the phase and magnitude of a voltage or current, a difference in voltage and current, power, force, temperature, or an interference fringe. For example, we deduce electric and magnetic field strengths and phase through Ampere’s and Faraday’s laws by means of voltage and current measurements. The scattering parameters measured on a network analyzer relates to the phase and magnitude of a voltage wave. The detection of a photon’s energy is sensed by an electron cascade current. Cavities and microwave evanescent probes sense material characteristics through shifts in resonance frequency from the influence of the specimen under test. The shift in resonance frequency is again determined by voltage and power measurements on a network analyzer. Magnetic interactions are also determined through measurements of current and voltage or forces [4, 7–9]. These measurement results are usually used with theoretical models, such as Maxwell’s equations, circuit parameters, or the Drude model, to obtain material properties.

High-frequency electrical responses include the measurement of the phase and magnitude of guided waves in transmission lines, fields from antennas, resonant frequencies and quality factors (Q) of cavities or dielectric resonators, voltage waves, movement of charge or spin, temperature changes, or forces on charge or spins. These responses are then combined through theoretical models to obtain approximations to important fundamental quantities such as: power, impedance, capacitance, inductance, conductance, resistance, conductivity, resistivity, dipole and spin moments, permittivity, and permeability, resonance frequency, Q, antenna gain, and near-field response [10–16].

The homogenization procedure used to obtain the macroscopic Maxwell equations from the microscopic Maxwell equations is accomplished by averaging the molecular dipole moments within a unit cell and constructing an averaged continuous charge density function. Then a Taylor series expansion of the averaged charge density is performed, and, as a consequence, it is possible to define the averaged polarization vector. The spatial requirement for the validity of this averaging is that the wavelength must be much larger than the unit cell dimensions (see Sec. 4.6). According to this analysis, the permittivity of an ensemble of molecules is valid for applied field wavelengths that are much larger than the dimensions of an ensemble of molecules or lattice, assuming one can isolate the effects of the molecules from the measurement apparatus. This metrology is not always easy because a measurement contains effects of electrodes, probes, and other environmental factors. The concepts of atomic polarizability and dipole molecular moment are valid on a smaller scale than are permittivity and permeability.

In the absence of an applied field, small random voltages with a zero mean are produced by equilibrium thermal fluctuations of random charge motion [17]. Fluctuations of these random voltages create electrical noise power in circuits. Analogously, spin noise is due to spin fluctuations. Quasi-monochromatic surface waves can also be excited by random thermal fluctuations. These surface waves are different from blackbody radiation [18]. Various interesting effects are achieved by random fields interacting with surfaces. For example, surface waves on two closely spaced surfaces can cause an enhanced radiative transfer. Noise in nonequilibrium systems is becoming more important in nanoscale measurements and in systems where the temperatures vary in time. The information obtained from radiometry at a large scale, or microscopic probing of thermal fluctuations of various material quantities, can produce an abundance of information on the systems under test.

### 1.4 RF Measurements at Various Scales

At RF frequencies the wavelengths are much larger than molecular dimensions. There are various approaches to obtaining material response with long wavelength fields to study small-scale particles or systems. These methods may use very sensitive detectors, such as single-charge or spin detectors or amplifiers, or average the response over an ensemble of particles to obtain a collective response. To make progress in the area of mesoscale measurement, detector sensitivity may need to exceed the three or four significant digits obtained from network analyzer scattering parameter measurements, or one must use large ensembles of cells for a bulk response and infer the small-scale response. Increased sensitivity may be obtained by using resonant methods or evanescent fields.

Material properties such as collective polarization and loss [19] are commonly obtained by immersing materials in the fields of EM cavities, dielectric resonators, free-space methods, or transmission lines. Some responses relate to intrinsic resonances in a material, such as polariton or plasmon response, ferromagnetic and anti-ferromagnetic resonances, and terahertz molecular resonances.

Broadband response is usually obtained by use of transmission lines or antenna-based systems [12–14, 19, 20]. Thin films are commonly measured with coplanar waveguides or microstrips [14]. Common methods used to measure material properties at small scales include near-field probes, micro-transmission lines, atomic-force microscopes, and lenses.

In strong fields, biological cells may rotate, deform, or be destroyed [21]. In addition, when there is more than one particle in the applied field, the fields between the particles can be modified by the presence of nearby particles. In a study by Friend et al. [22], the response of an amoeba to an applied field was studied in a capacitor at various voltages, power, and frequencies. They found that at 1 kHz and at 10 V/cm the amoeba oriented perpendicular to the field. At around 10 kHz and above 15 V/cm the amoeba’s internal membrane started to fail. Above 100 kHz and a field strength of above 50 V/cm, thermal effects started to damage the cells.

### 1.5 Electromagnetic Measurement Problems Unique to Microscale and Nanoscale Systems

Usually, the electrical skin depth for field penetration is much larger than the dimensions of nanoparticles. Because nanoscale systems are only 10 to 1000 times larger than the scale of atoms and small molecules, quantum mechanics plays a role in the transport properties. Below about 10 nm, many of the continuous quantities in classical electromagnetics take on a quantized aspect. These include charge transport, capacitance, inductance, and conductance. Fluctuations in voltage and current also become more important than in macroscopic systems. Electrical conduction at the 10 nanoscale involves movement of a small number of charge carriers through thin structures and may attain ballistic transport. For example, if a 1 *μ*A charge travels through a nanowire of radial dimensions 30 nm, then the current density is on the order of 3 × 10^9^ A/m^2^. Because of these large current densities, electrical transport in nanoscale systems is usually a non-equilibrium process, and there is a large influence of electron-electron and electron-ion interactions.

In nanoscale systems, boundary layers and interfaces strongly influence the electrical properties, and the local permittivity may vary with position [23]. Measurements on these scales must model the contact resistance between the nanoparticle and the probe or transmission line and deal with noise.

## 2. Fundamental Electromagnetic Parameters and Concepts Used in Material Characterization

### 2.1 Electrical Parameters for High-Frequency Characterization

In this section, the basic concepts and tools needed to study and interpret dielectric and magnetic response over RF frequencies are reviewed [24].

In the time domain, material properties can be obtained by analyzing the response to a pulse or impulse; however most material measurements are performed by subjecting the material to time-harmonic fields.

The most general causal linear time-domain relationships between the displacement and electric fields and induction and magnetic fields are
(1)
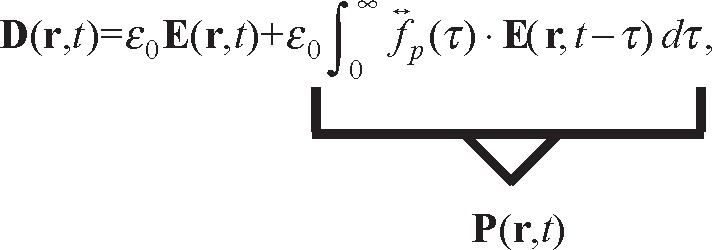

where ${\overset{↔}{f}}_{p}(t)$ is a polarization impulse-response dyadic,
(2)
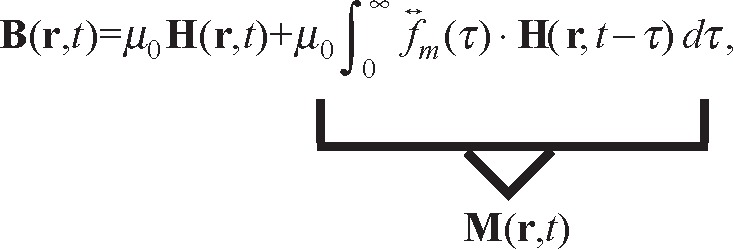

where ${\overset{↔}{f}}_{m}(t)$ is a magnetic impulse-response dyadic.

The permittivity $\overset{↔}{\varepsilon }(\omega )$ dyadic is the complex parameter in the time-harmonic field relation $\tilde{D}(\omega )=\overset{↔}{\varepsilon }(\omega )\cdot \tilde{E}(\omega )$ and, is defined in terms of the Fourier transform of the impulse-response function. For isotropic linear media, the scalar complex relative permittivity ε*_r_* is defined in terms of the absolute permittivity ε and the permittivity of vacuum ε_0_ (F/m), as follows ε(ω) = ε_0_ε*_r_*(ω), where $\varepsilon_{r}(\omega )=\varepsilon_{r\infty }+\chi_{r}(\omega )=\varepsilon_{r}^{\prime }(\omega )-i\varepsilon_{r}^{\prime \prime }(\omega )$, and ε*_r_*_∞_ is the optical-limit of the relative permittivity. The value of the permittivity of free space is $\varepsilon_{0}\equiv 1/\mu_{0}c_{\nu }^{2}\approx 8.854\times 10^{-12}\left(F/m\right)$, where the speed of light in vacuum is *c*_ν_ ≡ 299792458 (m/s) and the exact value of the permeability of free space is *μ*_0_ = 4π × 10^−7^ (H/m). Also, $\tan\delta_{d}=\varepsilon_{r}^{\prime \prime }/\varepsilon_{r}^{\prime }$ is the loss tangent in the material [25].

Note that in the SI system of units the speed of light, permittivity of vacuum, and permeability of vacuum are defined constants. All measurements are related to a frequency standard. Note that the minus sign before the imaginary part of the permittivity and permeability is due to the *e^i^*^ω^*^t^* time dependence. A subscript *eff* on the permittivity or permeability releases the quantity from some of the strict details of electrodynamic analysis. The permeability in no applied field is: $\mu (\omega )=\mu_{0}(\mu_{r}^{\prime }(\omega )-i\mu_{r}^{\prime \prime }(\omega ))$ and the magnetic loss tangent is $\tan\delta_{m}=\mu_{r}^{\prime \prime }(\omega )/\mu_{r}^{\prime }(\omega )$.

For anisotropic and gyrotropic media with an applied magnetic field, the permittivity and permeability tensors are hermitian and can be expressed in the general form

(3) $$\overset{↔}{\varepsilon }(\omega )=\left(\begin{array}{ccc} \varepsilon_{xx} & \varepsilon_{xy}-ig_{z} & \varepsilon_{xz}+ig_{y}\\ \varepsilon_{xy}+ig_{z} & \varepsilon_{yy} & \varepsilon_{yz}-ig_{x}\\ \varepsilon_{xz}-ig_{y} & \varepsilon_{yz}+ig_{x} & \varepsilon_{zz} \end{array}\right).$$

For a definition of gyrotropic media see [4]. The off-diagonal elements are due to gyrotropic behavior in an applied field.

Electric and magnetic fields are attenuated as they travel through lossy materials. Using time-harmonic signals the loss can be studied at specific frequencies, where the time dependence is *e^i^*^ω^*^t^*. The change in loss with frequency is related to dispersion.

The propagation coefficient of a plane wave is $\gamma =\alpha +i\beta =ik=i\omega \sqrt{\varepsilon \mu }$. The plane-wave attenuation coefficient in an infinitely thick half space, where the guided wavelength of the applied field is much longer than the size of the molecules or inclusions, is denoted by the quantity α and the phase is denoted by β. Due to losses of a plane wave, the wave amplitude decays as |**E**| ∝ exp(−α*z*). The power in a plane wave of the form *E*(*z*, *t*) = *E*_0_ exp (−α*z*) exp (*i*ω*t* − *i*β*z*), attenuates as *P* ∝ exp (−2α*z*). For waves in a guided structure: $\gamma =i\sqrt{k^{2}-k_{c}^{2}}$, where *k_c_* = *ω_c_/c* = 2*π*/*λ_c_* is the cutoff wavenumber, and speed of light *c*. Below cutoff, the propagation coefficient becomes $\gamma =\sqrt{k_{c}^{2}-k^{2}.}\;\alpha$ of a plane wave is given by

(4) $$\alpha =\frac{\omega }{c\sqrt{2}}\sqrt{\varepsilon_{r}^{\prime }\mu_{r}^{\prime }}{\left({\left({\left(\tan\delta_{d}\tan\delta_{m}-1\right)}^{2}+{\left(\tan\delta_{d}+\tan\delta_{m}\right)}^{2}\right)}^{1/2}+\left(\tan\delta_{d}\tan\delta_{m}-1\right)\right)}^{1/2},$$

and has units of Np/m. α is approximated for dielectric materials as

(5) $$\alpha =\frac{\omega }{c\sqrt{2}}\sqrt{\varepsilon_{r}^{\prime }\mu_{r}^{\prime }}\sqrt{\sqrt{1+\tan^{2}\delta_{d}}-1}.$$

In dielectric media with low loss, tanδ*_d_* ≪1, and α reduces in this limit to $\alpha \to \omega \sqrt{\varepsilon_{r}^{\prime }\mu_{r}^{\prime }}\tan\delta_{d}/2c$. The skin depth is the distance a plane wave travels until it decays to 1/*e* of its initial amplitude, and is related to the attenuation coefficient by δ_s_ = 1/α. The concept of skin depth is useful in modeling lossy dielectrics and metals. Energy conservation constrains a to be positive. The skin depth is defined for lossy dielectric materials as

(6) $$\delta_{s}=\frac{c\sqrt{2}}{\omega }\frac{1}{\sqrt{\varepsilon_{r}^{\prime }\mu_{r}^{\prime }}\sqrt{\sqrt{1+\tan^{2}\delta_{d}}-1}}.$$

In Eq. (6), δ*_s_* reduces in the low-conductivity limit to to $\delta_{s}\to 2c/(\omega \sqrt{\varepsilon_{r}^{\prime }\mu_{r}^{\prime }}\tan\delta_{d})$. The depth of penetration *D_p_* = δ_s_/2 is the depth where the plane-wave energy drops to 1/*e* of its value on the surface. In metals, where the conductivity is large, the skin depth reduces to

(7) $$\delta_{s}=\frac{1}{\sqrt{\pi f\mu_{0}\mu_{r}^{\prime }\sigma_{dc}}}$$

where σ*_dc_* is the dc conductivity and *f* is the frequency. We see that the frequency, conductivity, and permeability of the material determine the skin depth in metals.

The phase coefficient β for a plane wave is given by

(8) $$\beta =\pm \frac{\omega }{c\sqrt{2}}\sqrt{\varepsilon_{r}^{\prime }\mu_{r}^{\prime }}{\left({\left({\left(\tan\delta_{d}\tan\delta_{m}-1\right)}^{2}+{\left(\tan\delta_{d}+\tan\delta_{m}\right)}^{2}\right)}^{1/2}-\left(\tan\delta_{d}\tan\delta_{m}-1\right)\right)}^{1/2}.$$

In dielectric media, β reduces to

(9) $$\beta =\pm \frac{\omega }{c\sqrt{2}}\sqrt{\varepsilon_{r}^{\prime }\mu_{r}^{\prime }}\sqrt{\sqrt{1+\tan^{2}\delta_{d}}+1}.$$

The imaginary part of the propagation coefficient defines the phase of an EM wave and is related to the refractive index by $n=\pm \sqrt{\varepsilon_{r}^{\prime }\mu_{r}^{\prime }}$. In normaldielectrics the positive square root is taken in Eq. (8). Veselago [26] developed a theory of negative-index materials (NIM) where he used negative intrinsic $\varepsilon_{r}^{\prime }$ and $\mu_{r}^{\prime }$, and the negative square root in Eq. (8) is used. There is controversy over the interpretation of metamaterial NIM electrical behavior since the permeability and permittivity are commonly effective values. We will use the term NIM to describe materials that achieve negative effective permittivity and permeability over a band of frequencies.

The wave impedance for a transverse electric and magnetic mode (TEM) is $\sqrt{\mu /\varepsilon }$; for a transverse electric mode (TE) is *i*ω*μ*/*γ*, and for a transverse magnetic mode (TM) is *γ*/*i*ωε. The propagating plane wave wavelength in a material is decreased by a permittivity greater than that of vacuum; for example, for a TEM mode, $\lambda \approx c_{vac}/(\sqrt{\varepsilon_{r}^{\prime }\mu_{r}^{\prime }}f)$. In waveguides the guided wavelength *λ_g_* dependson the cutoff wavelength *λ_c_* and is given by $\lambda_{g}=1/\sqrt{\varepsilon^{\prime }\mu^{\prime }f^{2}/c^{2}-1/\lambda_{c}^{2}}=\lambda /\sqrt{1-{\left(\lambda /\lambda_{c}\right)}^{2}}$.

The surface impedance in ohms/square of a conducting material is *Z_m_* = (1 + *i*)σδ*_s_*. The surface resistance for highly lossy materials is

(10) $$R_{s}=\frac{1}{\delta_{s}\sigma_{dc}}=\sqrt{\frac{\pi f\mu_{0}\mu_{r}^{\prime }}{\sigma_{dc}}}.$$

When the conductors on a substrate are very thin, the fields can penetrate through the conductors into the substrate. This increases the resistance of a propagating field because it is in both the metal and the dielectric. As a consequence of the skin depth, the internal inductance in a highly-conducting material decreases with increasing frequency, whereas the surface resistance *R_s_* increases with frequency in proportion to $\sqrt{f}$.

Any transmission line will have propagation delay that relates to the propagation speed in the line. This is related to the dielectric permittivity and the geometry of the transmission line. Propagation loss is due to conductor and material loss.

Some materials exhibit ionic conductivity, so that when a static electric field is applied, a current is induced. This behavior is modeled by the dc conductivity σ*_dc_*, which produces a low-frequency loss (∝ 1/ω) in addition to polarization loss $\left(\varepsilon_{r}^{\prime \prime }\right)$. In some materials, such as semiconductors and disordered solids, the conductivity is complex and depends on frequency. This is because the free charge is partially bound and moves by tunneling through potential wells or hops from well to well.

The total permittivity for linear, isotropic materials that includes both dielectric loss and dc conductivity is defined from the Fourier transform of Maxwell’s equation: $i\omega \;\tilde{D}(\omega )+\tilde{J}(\omega )\equiv i\omega \varepsilon \;\tilde{E}\;(\omega )+\sigma_{dc}\;\tilde{E}(\omega )\equiv i\omega \varepsilon_{tot}\;\tilde{E}\;(\omega )$, so that

(11) $$\varepsilon_{tot}=\varepsilon_{r}^{\prime }\varepsilon_{0}-i(\varepsilon_{r}^{\prime \prime }\varepsilon_{0}+\frac{\sigma_{dc}}{\omega }).$$

In plots of RF measurements, the decibel scale is often used to report power or voltage measurements. The decibel (dB) is a relative unit and for power is calculated by 10 log_10_ (*P*_out_/*P*_in_). Voltages in decibels are defined as 20 log_10_ (*V*_out_/*V*_in_). α has units of Np/m. The attenuation can be converted from 1 Np/m = 8.686 dB/m. dBm is similar to dB, but relative to power in milliwatts 10 log(*P*/mW).

### 2.2 Electromagnetic Power

In the time domain the internal field energy *U* satisfies: ∂*U*/∂*t* = ∂**D**/∂*t* · **E** + ∂**B**/∂*t* · **H**. Using Maxwell’s equations with a current density **J**, then produces Poynting’s Theorem: ∂*U*/∂*t +* ∇ · (**E** × **H**) = − **J** · **E**, where the time-domain Poynting vector is **S**(**r**, t) = **E**(**r**, *t*) × **H**(**r**, *t*). The complex power flux (W/m^2^) is summarized by the complex Poynting vector $S_{c}(\omega )==(1/2)(\tilde{E}\;(\omega )\times {\tilde{H}}^{*}\;(\omega ))$. The real part of **S***_c_* represents dissipation and is the time average over a complete cycle. The imaginary part of **S***_c_* relates to the reactive stored energy.

### 2.3 Quality Factor

The band width of a resonance is usually modeled by the quality factor (*Q*) in terms of the decay of the internal energy. The combined internal energy in a mechanical system is the kinetic plus the potential energy; in an electromagnetic system it is the field stored energy plus the potential energy. In the time domain the quality factor is related to the decay of the internal energy for an unforced resonator as as [27]

(12) $$\frac{dU(t)}{dt}=-\frac{\omega_{0}}{Q_{0}}U(t).$$

The EM field is modeled by a damped harmonic oscillator at frequencies around the lossless resonant frequency ω_0_ and frequency pulling factor (the resonant frequency decreases from ω_0_ due to material losses), Δω as [27]

(13) $$E(t)=E_{0}e^{-\omega_{0}t/2Q_{0}}e^{i(\omega_{0}+\Delta \omega )t}.$$

**Fig. 2:**
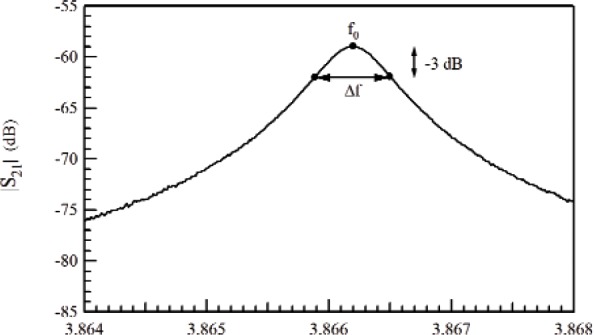
Measuring resonant frequency and Q.

Taking a Fourier transform of Eq. (13), the absolute value squared becomes

(14) $${\left|E(\omega )\right|}^{2}=\frac{A}{{\left(\omega -\omega_{0}-\Delta \omega \right)}^{2}+{\left(\frac{\omega }{2Q_{0}}\right)}^{2}},$$

and therefore |*E*(ω)|^2^, which is proportional to the power, is a Lorentzian. This linear model is not exact for dispersive materials, because *Q*_0_ may be dependent on frequency. The quality factor is calculated from the frequency at resonance *f*_0_ as *Q*_0_ = *f*_0_/2(|*f*_0_ − *f*_3_*_dB_*|), or from a fit of a circle when plotting *S*_11_(ω) on the Smith chart. The quality factor is calculated from *Q*_0_ = *f*_0_/Δ*_f_*, where Δ*f* is the frequency difference between 3 dB points on the *S*_21_ curve [28]. For resonant cavity measurements, the permittivity or permeability is determined from measurements of the resonance frequency and quality factor, as shown in Fig. 2. For time-harmonic fields the *Q* is related to the stored field energies *W_e_*,*W_h_*, the angular frequency at resonance ω*_r_*, and the power dissipated *P_d_* at the resonant frequency:

(15) $$Q=\omega_{r}\frac{W_{e}+W_{h}}{P_{d}}.$$

Resonant frequencies can be measured with high precision in high-Q systems; however the parasitic coupling of the fields to fixtures or materials needs to be modeled in order to make the result meaningful. Material measurements using resonances have much higher precision than using nonresonant transmission lines.

The term antiresonance is used when the reactive part of the impedance of a EM system is very high. This is in contrast to resonance, where the reactance goes to zero. In a circuit consisting of a capacitor and inductance in parallel, antiresonance occurs when the voltage and current are in phase.

## 3. Maxwell’s Equations in Materials

### 3.1 Maxwell’s Equations From Microscopic to Macroscopic Scales

Maxwell’s microscopic equations in a media with charged particles are written in terms of the microscopic fields **b**, **e** and sources **j**, and ρ*_m_* as

(16) $$\nabla \times b=\varepsilon_{0}\mu_{0}\frac{\partial e}{\partial t}+\mu_{0}j,$$

(17) $$\nabla \times e=-\frac{\partial b}{\partial t},$$

(18) $$\varepsilon_{0}\nabla \cdot e=\rho_{m},$$

(19) ∇⋅b=0.

Note, that at this level of description the macroscopic magnetic field **H** and the macroscopic displacement field **D** are not defined, but can be formed by averaging dielectric and magnetic moments and expanding the microscopic charge density in a Taylor series. In performing the averaging process, the material length scales allow the dipole moments in the media to be approximated by continuously varying functions **P** and **M**. Once the averaging is completed, the macroscopic Maxwell’s equations are (see Sec. 4.6) to obtain [27, 29, 30]

(20) $$\nabla \times H=\frac{\partial D}{\partial t}+J,$$

(21) $$\nabla \times E=-\frac{\partial B}{\partial t},$$

(22) ∇⋅D=ρ,

(23) ∇⋅B=0.

**J** denotes the current density due to free charge and source currents. Because there are more unknowns than equations, constitutive relations for **H** and **D** are needed. Even though **B** and **E** are the most fundamental fields, **D** usually is expressed in terms of **E**, and **B** is usually expressed in terms of **H**.

### 3.2 Constitutive Relations

#### 3.2.1 Linear Constitutive Relations

Since there are more unknowns than macroscopic Maxwell’s equations, we must specify the constitutive relationships between the polarization, magnetization, and current density as functions of the macroscopic electric and magnetic fields [31, 32]. In order to satisfy the requirements of linear superposition, any linear polarization relation must be time invariant, further, this must also be a causal relationship as given in Eqs. (1) and (2).

The fields and material-related quantities in Maxwell’s equations must satisfy underlying symmetries. For example, the dielectric polarization and electric fields are odd under parity transformations and even under time-reversal transformations. The magnetization and induction fields are even under parity transformation and odd under time reversal. These symmetry relationships place constraints on the nature of the allowed constitutive relationships and requires the constitutive relations to manifest related symmetries [29, 33–39]. The evolution equations for the constitutive relationships need to be causal, and in linear approximations must satisfy time-invariance properties. For example, the linear-superposition requirement is not satisfied if the relaxation time in Eq. (4) depends on time. This can be remedied by using an integrodifferential equation with restoring and driving terms [40, 41].

The macroscopic displacement and induction fields **D** and **B** are related to the macroscopic electric field **E** and magnetic fields **H**, as well as **M** and **P**, by

(24) $$D=\varepsilon_{0}E+P_{d}-\nabla \cdot \overset{↔}{Q}+\ldots \equiv \varepsilon_{0}E+P,$$

and

(25) $$B=\mu_{0}H+\mu_{0}M.$$

In addition,

(26) J=J(E,H),

where *J* is a function of the electric and magnetic fields, and $\overset{↔}{Q}$ is the macroscopic quadrupole moment density. **P***_d_* is the dipolemoment density, whereas **P** is the effective macroscopic polarization that also includes the effects of the macroscopic quadrupole-moment density [27, 29, 30, 32, 42]. The polarization and magnetization for time-domain linear response are expressed as convolutions in terms of the macroscopic fields. For chiral and magneto-electric materials, Eqs. (24) and (25) must be modified to accommodate cross-coupling behavior between magnetic and dielectric response. General, linear relations defining polarization in non-magnetoelectric and non-chiral dielectric and magnetic materials in terms of the impulse-response dyadics are given by Eqs. (1) and (2). Using the Laplace transform ℒ, gives

(27) $$L|\overset{\approx }{N}|(\omega )={\overset{↔}{\chi }}_{er}(\omega )\tilde{E}(\omega ),$$

where

(28) $${\overset{↔}{\chi }}_{er}(\omega )=\int_{0}^{\infty }{\overset{↔}{f}}_{p}(\tau )e^{-i\omega t}dt={\overset{↔}{\chi }}^{\prime }{}_{er}(\omega )-i{\overset{↔}{\chi }}^{\prime \prime }{}_{er}(\omega ).$$

So the real part is the even function of frequency given by

(29) $${\overset{↔}{\chi }}^{\prime }{}_{er}(\omega )=\int_{0}^{\infty }{\overset{↔}{f}}_{p}(t)\cos(\omega t)dt,$$

and the imaginary part is an odd function of frequency

(30) $${\overset{↔}{\chi }}^{\prime \prime }{}_{er}(\omega )=\int_{0}^{\infty }{\overset{↔}{f}}_{p}(t)\sin(\omega t)dt,$$

and therefore

(31) $${\overset{↔}{\varepsilon }}_{r}(\omega )=\overset{↔}{I}+{\overset{↔}{\chi }}^{\prime }{}_{er}(\omega )-i{\overset{↔}{\chi }}^{\prime \prime }{}_{er}(\omega );$$

also

(32) $$\overset{↔}{\chi_{er}^{\prime }}(0)=\int_{0}^{\infty }\overset{↔}{f_{p}}(t)dt,$$

(33) $$\overset{↔}{\chi_{er}^{\prime \prime }}(0)=0.$$

The time-evolution constitutive relations for dielectric materials are generally summarized by generalized harmonic oscillator equations or Debye-like equations as overviewed in Sec. 5.2.

#### 3.2.2 Generalized Constitutive Relations

Through the methods of nonequilibrium quantum-based statistical-mechanics it is possible to show that the constitutive relation for the magnetization in ferromagnetic materials is an evolution equation given by

(34) $$\begin{array}{c} \frac{\partial M(r,t)}{\partial t}=-|\gamma_{g}|M(r,t)\times H_{eff}(r,t)\\ -\int d^{3}r^{\prime }\int_{0}^{t}{\overset{\leftarrow }{K}}_{m}(r,t,r^{\prime },\tau )\cdot \chi_{0}H_{eff}(r^{\prime },\tau )d\tau , \end{array}$$

where ${\overset{↔}{K}}_{m}$ is a kernel that contains of the microstructural interactions given in [43], γ*_g_* is the gyromagnetic ratio, χ_0_ is the static susceptibility, and **H***_eff_* is the effective magnetic field. Special cases of Eq. (34) reduce to constitutive relations such as the Landau-Lifshitz, Gilbert, and Bloch equations. The Landau-Lifshitz equation of motion is useful for ferromagnetic and ferrite solid materials:
(35)
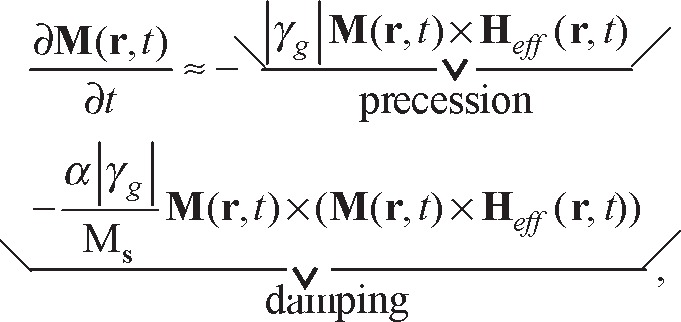

where α is a damping constant. Another special case of Eq. (34) reduces to the Gilbert equation

(36) $$\begin{array}{r} \frac{\partial M(r,t)}{\partial t}\approx -|\gamma_{g}|M(r,t)\times H_{eff}(r,t)\\ +\frac{\alpha }{M_{s}}M(r,t)\times \frac{\partial M(r,t)}{\partial t}. \end{array}$$

In electron-spin resonance (EPR) and nuclear magnetic resonance (NMR) measurements, the Bloch equations with characteristic relaxation times *T*_1_ and *T*_2_ are used to model relaxation. *T*_1_ relates to spin-lattice relaxation as the paramagnetic material interacts with the lattice. *T*_2_ relates to spin-spin interactions:

(37) $$\begin{array}{r} \frac{\partial M(r,t)}{\partial t}\approx -|\gamma_{g}|M(r,t)\times H(r,t)\\ -{\overset{↔}{\chi }}_{b}\cdot M(r,t)+\frac{M_{s}}{T_{1}}, \end{array}$$

where ${\overset{↔}{\chi }}_{b}$ has only the diagonal elements χ*_b_*_(11)_ = 1/*T*_2_, χ*_b_*_(22)_ = 1/*T*_2,_ χ*_b_*_(33)_ = 1/*T*_1_, and $M_{s}=M_{s}\vec{z}$. An equation analogous to (34) can be written for the electrical polarization [46] as [43]

(38) $$\begin{array}{r} \frac{\partial P(r,t)}{\partial t}=-\int d^{3}r^{\prime }\int_{0}^{t}{\overset{↔}{K}}_{e}(r,t,r^{\prime },\tau )\\ \times \left(P(r^{\prime },\tau )-{\overset{↔}{\chi }}_{0}\cdot E(r^{\prime },\tau )d\tau \right). \end{array}$$

The Debye relaxation differential equation is recovered from Eq. (38) when ${\overset{↔}{K}}_{e}(r,t,r^{\prime },\tau )=\overset{↔}{I}\delta (t-\tau )\delta (r,-r^{\prime })/\tau_{e}$.

## 4. Electromagnetic Fields in Materials

### 4.1 The Time-Harmonic Field Approximation

Time-harmonic fields are very useful for solving the linear Maxwell’s equations when transients are not important. In the time harmonic field approximation, the field is assumed to be present without beginning or end. Periodic signals over − ∞ < *t* < ∞ are nonphysical since all fields have a beginning where transients are generated, but are very useful in probing material response.

Solutions of Maxwell’s equations that include transients are most easily obtained with the Laplace transform. Note that the Laplace or Fourier transformed fields do not have the same units as the time-harmonic fields due to integration over time. In Eq. (1), causality is incorporated into the convolution relation for linear response. **D**(*t*) depends only on **E**(*t*) at earlier times and not future times.

### 4.2 Material Response to Applied Fields

**Fig. 3:**
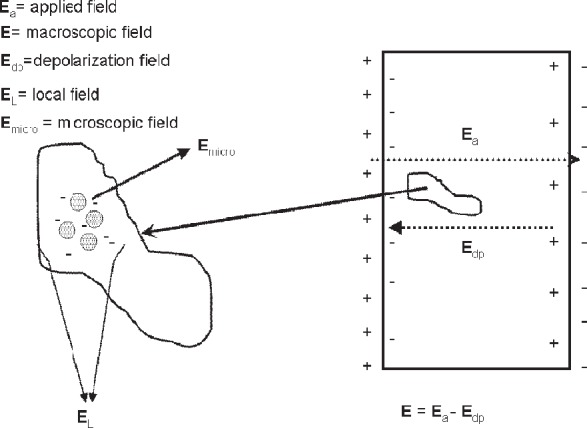
Fields in materials.

When a field is suddenly applied to a material, the charges, spins, currents, and dipoles in a medium respond to the local fields to form an average field. If an EM field is suddenly applied to a semi-infinite material, the total field will include the effects of both the applied field, transients, and the particle back-reaction fields from charge, spin, and current rearrangement that causes depolarization fields. This will cause the system to be in nonequilibrium for a period of time. For example, as shown in Fig. 3, when an applied EM field interacts with a dielectric material, the dipoles reorient and charge moves, so that the macroscopic and local fields in the material are modified by surface charge dipole depolarization fields that oppose the applied field. The macroscopic field is approximately the applied field minus the depolarization field. Depolarization, demagnetization, thermal expansion, exchange, nonequilibrium, and anisotropy interactions can influence the dipole orientations and therefore the fields and the internal energy. In modeling the constitutive relations in Maxwell’s equations, we must express the material properties in terms of the macroscopic field, not the applied or local fields, and therefore we need to make clear distinctions between the interaction processes [40].

Materials can be studied by the response of frequency-domain or time-domain fields. When considering time-domain pulses rather than time-harmonic fields, this interaction is more complex. The use of time-domain pulses have the advantage of sampling a reflected pulse as a function of time, which allows a determination of the spatial location of the various reflections.

Time-harmonic fields are often used to study material properties. These have a specific frequency from time minus infinity to plus infinity, without transients; that is, fields with a *e^i^*^ω^*^t^* time dependence. As a consequence, in the frequency domain, materials can be studied through the reaction to periodic signals. The measured response relates to how the dipoles and charge respond to the time-harmonic signal at each frequency. If the frequency information is broad enough, a Fourier transform can be used to study the corresponding time-domain signal.

The relationships between the applied, macroscopic, local, and the microscopic fields are important for constitutive modeling (Fig. 3). The applied field originates from external charges, whereas the macroscopic fields are averaged quantities in the medium. The displacement and inductive (or magnetic) macroscopic fields in Maxwell’s equations are implicitly defined through the constitutive relationships and boundary conditions. The local field is the averaged EM field at a particle site due to both the applied field and the fields from all of the other sources, such as dipoles, currents, charge, and spin [47]. The microscopic field represents the atomic-level EM field, where particles interact with the field from discrete charges. Particles interact with the local EM field that is formed from the applied field and the microscopic field. At the next level of homogenization, groups of particles interact with the macroscopic field. The spatial and temporal resolution contained in the macroscopic variables are directly related to the spatial and temporal detail incorporated in the constitutive material parameters. Constitutive relations can be exact as in [40] and Eqs. (34) and (38), but usually, to be useful, are approximate.

Plane waves are a useful approximation in many applications. Time-harmonic EM plane waves in materials can be treated either as traveling without attenuation, propagating with attenuation, or evanescent. Plane waves may propagate in the form of a propagating wave *e ^i^*^(ω^*^t^*^−β^*^z^*^)^, or a damped propagating wave *e ^i^*^(ω^*^t^*^−β^*^z^*^) −α^*^z^*, or an evanescent wave *e^i^*^ω^*^t^*^−α^*^z^*. Evanescent fields are exponentially damped waves. In a waveguide, this occurs for frequencies below any transverse resonance frequencies [24, 48], when $k^{2}-k_{c}^{2}<0$, where *k_c_* is the cutoff wave number calculated from the transverse geometry and $k=\omega \sqrt{\varepsilon \mu }=(\omega /c)\sqrt{\varepsilon_{r}^{\prime }\mu_{r}^{\prime }}$. Evanescent and near field EM fields occur at apertures and in the vicinity of antennas. Evanescent fields can be detected when they are perturbed and converted into propagating waves or transformed by dielectric loss. Electromagnetic waves may convert from near field to propagating. For example, in coupling to dielectric resonators the near field at the coupling loops produce propagating or standing waves in a cavity or dielectric resonator. Evanescent and near fields in dielectric measurements are very important. These fields do not propagate and are used in near-field microwave probes to measure or image materials at dimensions much less than λ/2 [49, 50] (see Fig. 17). The term near field usually refers to the waves close to an waveguide, antenna, or probe and is not necessarily an exponentially damped plane wave. In near-field problems the goal is to model the reactive region. Near fields in the reactive region, (*L* < λ/2π), contain stored energy and there is no net energy transport over a cycle unless there are losses in the medium. By analogy, the far field relates to radiation. These remove energy from the transmitter whether they are immediately absorbed or not. There is a transition region called the radiative near field.

Because electrical measurements can now be performed at very small spatial resolutions, and the elements of electrical circuits are approaching the molecular level, we require good models of the macroscopic and local fields. This is particularly important, because we know that the Lorentz theory of the local field is not always adequate for predicting polarizabilities [51, 52]. Also, when solving Maxwell’s equations at the molecular level, definitions of the macroscopic field and constituative relationships are important. A theoretical analysis of the local EM field is important in dielectric modeling of single-molecule measurements and thin films. The effective EM fields at this level are local, but not atomic-scale, fields.

The formation of the local field is a very complex process whereby the applied electric field polarizes dipoles in a molecules or lattices and the applied magnetic field causes current and precession of spins. Then, the molecule’s dipole field modifies the dipole orientations of other molecules in close proximity, which then reacts back to produce a correction to the molecule’s field in the given region. This process gets more complicated for behavior that depends on time. We define the local EM field as the effective, averaged field at a specific point in a material, not including the field of the particle itself. This field is a function of both the applied and multipole fields in the media. The local field is related to the average macroscopic and microscopic EM fields in that it is a sum of the macroscopic field and the effects of the near-field. In ferroelectric materials, the local electric field can become very large and hence there is a need for comprehensive local field models. In the literature on dielectric materials, a number of specific fields have been introduced to analyze polarization phenomena. The electric field acting on a nonpolar dielectric is commonly called the internal field, whereas the field acting on a permanent dipole moment is called the directing field. The difference between the internal field and directing fields is the average reaction field. The reaction field is the result of a dipole polarizing its environment [53].

Nearly exact classical theories have been developed for the static local field. Mandel and Mazur developed a static theory for the local field in terms of the polarization response of a many-body system by use of the T-matrix formalism [54]. Gubernatis extended the T-matrix formalism [55]. However, the T-matrix contributions are difficult to calculate. Keller’s review article [56] on the local field uses an EM propagator approach. Kubo’s linear-response theory and other theories have also been used for EM correlation studies [40, 53, 57].

If the applied field has a wavelength that is not much longer than the typical particle size in a material, an effective permittivity and permeability is commonly assigned. The terms effective permittivity and permeability are commonly used in the literature for studies of composite media. The assumption is that the properties are “effective” if in some sense they do not adhere to the definitions of the intrinsic material properties. An effective permittivity is obtained by taking a ratio of some averaged displacement field to an averaged electric field. The effective permeability is obtained by taking a ratio of some averaged induction field to an averaged magnetic field. This approach is commonly used in modeling negative-index material properties when scatterers are designed in such a manner such that the scatterers themselves resonate. In these situations the wavelength may approach the dimensions of the inclusions.

### 4.3 Macroscopic and Local Electromagnetic Fields in Materials

The mesoscopic description of the EM fields in a material is complicated. As a field is applied to a material, charges reorient to form new fields that oppose the applied field. In addition, a dipole tends to polarize its immediate environment, which modifies the field the dipole experiences. The field that polarizes a molecule is the local field **E***_l_* and the induced dipole moment is $p=\overset{↔}{\alpha }\cdot E_{l}$, where $\overset{↔}{\alpha }$ is the polarizability. In order to use this expression in Maxwell’s equations, the local field needs to be expressed in terms of the macroscopic field. Calculation of this relationship is not always simple.

To first approximation, the macroscopic field is related to the external or applied field (**E***_a_*), and the depolarization field by

(39) $$E=E_{a}-\frac{1}{3\varepsilon_{0}}P.$$

The local field is composed of the macroscopic field and a material-related field. In the literature, the effective local field is commonly modeled by the Lorentz field, which is defined as the field in a small cavity that is carved out of a material around a specific site, but excludes the field of the observation dipole. A well-known example of the relationship between the applied, macroscopic, and local fields is given by an analysis of the Lorentz spherical cavity in a static electric field. For a Lorentz sphere the local field is the sum of applied, depolarization, Lorentz, and atomic fields [4, 56, 58]:

(40) $$E_{t}=E_{a}+E_{depol}+E_{Lorentz}+E_{atom}.$$

For cubic lattices in a spherical cavity, the Lorentz local field is related to the macroscopic field and polarization by

(41) $$E_{l}=E+\frac{1}{3\varepsilon_{0}}P.$$

In the case of a sphere, the local field in Eq. (39) equals the applied field.

For induced dipoles,

(42) $$P=N\alpha E_{l},$$

where *N* is the density of dipoles, and Eq. (41) yields **E**_l_ = **E**/(1 − *N*α/3ε_0_) = **P**/Nα.

Onsager [53] generalized the Lorentz theory by distinguishing between the internal field that acts on induced dipoles and the directing field that acts on permanent dipoles. If we use **P** = ε_0_ (ε*_r_* − 1)**E** in Eq. (41), we find **E***_l_* = ((ε*_r_* + 2)/3)**E**. Therefore, for normal materials the Lorentz field exceeds the macroscopic field. For a material where the permittivity is negative we can have **E***_l_* ≤ **E**. In principle, we can null out the Lorentz field when ε*_r_* = − 2. Some of the essential problems encountered in microscopic constitutive theory center around the local field. Note that for some materials, recent research indicates that the Lorentz local field does not always lead to the correct polarizabilities [51]. We expect the Lorentz local field expression to break down near interfaces. For nanoparticles, a more complicated theory needs to be used for the local field.

A rigorous expression for the static local field created by a group of induced dipoles can be obtained by an iterative procedure [53, 59] using **p***_i_* = α*_i_***E***_l_*(**r***_i_*) and

(43) $$E_{i}(r_{j})=E_{a}+\sum_{i=l,i\neq j}^{n}E_{ij}(r_{j}),$$

where

(44) $$E_{ij}(r_{j})\approx \frac{1}{4\pi \varepsilon_{0}},\left[\frac{3(r_{j}-r_{i})p(r_{i})}{{\left|r_{j}-r_{i}\right|}^{5}}-\frac{p(r_{i})}{{\left|r_{j}-r_{i}\right|}^{3}}\right].$$

If there are also permanent dipoles, they need to be included as **p**(**r***_i_*) = **p***_perm_*(**r***_i_*) + α*_i_***E***_l_*(**r***_i_*).

### 4.4 Overview of Linear-Response Theory

Models of relaxation that are based on statistical mechanics can be developed from linear-response theory. Linear-response theory uses an approximate solution of Liouville’s equation and a Hamiltonian that contains a time-dependent relationship of the field parameters based on a perturbation expansion. This approach shows how the response functions and relaxation are related to time dependent polarization correlation functions. The polarization **P**(*t*) is related to the response dyadic $\overset{↔}{\phi }(t)$ and the driving field **E**(*t*) by [53, 60]

(45) $$P(t)=\int_{-\infty }^{t}\overset{↔}{\phi }(t-\tau )\cdot E(\tau )d\tau ,$$

where $\overset{↔}{\phi }(\tau -\tau )=0$ for *t* − τ < 0. The susceptibility is defined as

(46) $$\overset{↔}{\chi }(\omega )=\int_{0}^{\infty }\overset{↔}{\phi }(\tau )e^{-i\omega \tau }d\tau ={\overset{↔}{\chi }}^{\prime }(\omega )-i{\overset{↔}{\chi }}^{\prime \prime }(\omega ),$$

where the response in volume *V* is related to the correlation function for stationary processes in terms of the microscopic polarization

(47) $$\overset{↔}{\phi }(\tau )=-V\frac{d}{d\tau }\frac{<p(0)p(\tau ){>}_{0}}{k_{B}T},$$

and therefore for microscopic polarizations

(48) $$\begin{array}{l} \overset{↔}{\chi }(\omega )=\int_{0}^{\infty }e^{-i\omega \tau }\overset{↔}{\phi }(\tau )d\tau =\\ \;-V\int_{0}^{\infty }e^{-i\omega \tau }\frac{d}{d\tau }\frac{<p(0)p(t){>}_{0}}{k_{B}T}d\tau \\ \;=\frac{V\omega }{k_{B}T}[\int_{0}^{\infty }<p(0)p(\tau ){>}_{0}\sin(\omega \tau )d\tau -\\ \;i\int_{0}^{\infty }<p(0)p(\tau ){>}_{0}\cos(\omega \tau )d\tau ]. \end{array}$$

Once the correlation functions are determined then the susceptibility can be found. An approach that models relaxation beyond linear response is given in [40, 43, 44, 61]. The method of linear response has exceeded expectations and has been a cornerstone of statistical mechanics.

### 4.5 Averaging to Obtain Macroscopic Field

If we consider modeling of EM wave propagation from macroscopic through molecular and sub-molecular to atomic scales, the effective response at each level is related to different degrees of homogenization. At wavelengths short relative to particle size the EM propagation is dominated by scattering, whereas at long wavelengths it is dominated by traveling waves. In microelectrodynamics, there have been many types of ensemble and volumetric averaging methods used to define the macroscopic fields obtained from the microscopic fields [27, 29, 30, 40, 54]. For example, in the most commonly used theory of microelectromagnetics, materials are averaged at a molecular level to produce effective molecular dipole moments. The microscopic EM theories developed by Jackson, Mazur, and Robinson [27, 29, 30] average multipoles at a molecular level and replace the molecular multipoles, with averaged point multipoles usually located at the center-of-mass position. This approach works well down to near molecular level, but breaks down below the molecular to submolecular level.

In the various approaches, the homogenization of the fields are formed in different ways. The averaging is always volumetric rather than a time average. Jackson uses a truncated averaging test function to proceed from microscale to the macroscale fields [27]. Robinson and Mazur use ensemble averaging [29, 30] and statistical mechanics. Ensemble averaging assumes there is a distribution of states. In the volumetric averaging approach, the averaging function is not explicitly determined, but the function is assumed to be such that the averaged quantities vary in a manner smooth enough to allow a Taylor-series expansion to be performed. In the approach of Mazur, Robinson, and Jackson [27, 29, 30] the charge density is expanded in a Taylor series and the multipole moments are identified as in Eq. (49). The microscopic charge density can be related to the macroscopic charge density, polarization, and quadrupole density by a Taylor-series expansion [27]

(49) $$<\rho_{micro}(r,t)>\approx \rho_{macro}(r,t)-\nabla \cdot P(r,t)-\nabla \cdot (\nabla \cdot \overset{↔}{Q})(r,t),$$

where $\overset{↔}{Q}(r,t)$ is the quadrupole tensor. In this interpretation, the concepts of **P** and ρ*_macro_* are valid at length scales where a Taylor-series expansion is valid. These moments are calculated about each molecular center of mass and are treated as point multipoles. However, this type of molecular averaging limits the scales of the theory to larger than the molecular level and limits the modeling of induced-dipole molecular moments [40]. Usually, the averaging approach uses a test function *f_a_* and microscopic field **e** given by

(50) $$E=\int dr^{\prime }e(r-r^{\prime })f_{a}(r^{\prime }).$$

However, the distribution function is seldom explicitly needed or determined in the analysis. The macroscopic magnetic polarization is found through an analogous expansion of the microscopic current density.

In NIM materials, effective properties are obtained by use of electric and magnetic resonances of embedded structures that produce negative effective ε′*_eff_* [62]. In Sec. 4.6 the issue of whether this response can be summarized in terms of material parameters is discussed. Defining permittivity and permeability on these scales of periodic media can be confusing. The field averaging used in NIM analysis is based on a unit cell consisting of split-ring resonators, wires, and ferrite or dielectric spheres [62, 63].

In order to obtain a negative effective permeability in NIM applications, researchers have used circuits that are resonant, which can be achieved by the introduction of a capacitance into an inductive system. Pendry et al. [63–65] obtained the required capacitance through gaps in split-ring resonators. The details of the calculation of effective permeability are discussed in Reference [63]. Many passive and/or active microwave resonant devices can be used as sources of effective permeability in the periodic structure designed for NIM applications [66]. We should note that the composite materials used in NIM are usually anisotropic. Also, the use of resonances in NIM applications produce effective material parameters that are spatially varying and frequency dispersive.

### 4.6 Averaging to Obtain Permittivity and Permeability in Materials

The goal of this section is to study the electrical permittivity and permeability in materials starting from microscopic concepts and then progressing to macroscopic concepts. We will study the limitations of the concept of permittivity in describing material behavior when wavelengths of the applied field approach the dimensions of the spaces between inclusions or inclusion sizes. When high-frequency fields are used in the measurement of composite and artificial structures, these length-scale constraints are important. We will also examine alternative quantities, such as dipole moment and polarizability, that characterize dielectric and magnetic interactions of molecules, atoms, and that are still valid even when the concepts of permittivity and permeability are fuzzy.

The concepts of polarizability and dipole moment **p** in **p** = α**E***_l_* are valid down to the atomic and molecular levels. Permittivity and permeability are frequency-domain concepts that result from the microscopic time-harmonic form of Maxwell’s equations averaged over a unit cell. They are also related to the Fourier transform of the impulse-response function. The most common way to define $\overset{↔}{\varepsilon }$ is through the impulse-response function ${\overset{↔}{f}}_{p}(t)$.

Statistical mechanics yields an expression for the impulse-response function in terms of correlation functions of the microscopic polarizations **p**. For linear response [53]

(51) $$\frac{dP}{dt}=\frac{V}{k_{B}T}\int_{0}^{t}<\dot{p}e^{i{ℒ}_{0}(t-\tau )}\dot{p}{>}_{0}\cdot Ep(\tau )d\tau ,$$

where *V* is the volume, ℒ_0_ is Liouville’s operator, $\dot{p}$ denotes *i*ℒ_0_**p**, and < >0 denotes averaging over phase. From this equation, we can identify the impulse-response dyadic ${\overset{↔}{f}}_{p}$ from $P(t)=V\int_{0}^{\infty }<P(t)\dot{P}(\tau ){>}_{0}\cdot E(\tau )d\tau /k_{B}T$, and for a stationary system, ${\overset{↔}{f}}_{p}(t)=V<P(0)\dot{P}(-t){>}_{0}/k_{B}T$ [53].

Ensemble and volumetric averaging methods are used to obtain the macroscopic fields from the microscopic fields (see Jackson [27] and the references therein). For example, in the most commonly used theory, materials are averaged at a molecular level to produce effective molecular dipole moments. When deriving the macroscopic Maxwell’s equations from the microscopic equations, the electric and magnetic multipoles within a molecule are replaced with averaged point multipoles usually located at the molecular center-of-mass positions. Then these effective moments are assumed to form a continuum, which then forms the basis of the macroscopic polarizations. The procedure assumes that the wavelength in the material is much larger than the individual particle sizes. As Jackson [27] notes, the macroscopic Maxwell’s equations can model refraction and reflection of visible light, but are not as useful for modeling x-ray diffraction. He states that the length scale *L*_0_ of 10 nanometers is effectively the lower limit for the validity of the macroscopic equations. Of course, this limit can be decreased with improved constitutive relationships.

For macroscopic heterogeneous materials the wavelengths of the applied fields must be much longer than individual particle or molecule dimensions that constitute the material. When this criterion does not hold, then the spatial derivative in the macroscopic Maxwell’s equations, for example, (∇ × H), and the displacement field loses its meaning. Associated with this homogenization process at a given frequency is the number of molecules or inclusions that are required to define a displacement field and thereby the related permittivity.

When the ratio of the dipole length scale to wavelength is not very small, the Taylor’s series expansion is not valid and the homogenization procedure breaks down. When this criteria is not satisfied for metafilms, some researchers use generalized sheet transition conditions (GSTC’s) [67–70] at the material boundaries; however, the concept of permittivity for these structures, at these frequencies, is still in question and is commonly assigned an effective value. Drude and others [67, 68] compensated for this by introducing boundary layers. In such cases, it is not clear whether mapping complicated field behavior onto effective permittivity and permeability is useful, since at these scales, the results can just as well be thought of as scattering behavior.

When modeling the permittivity or permeability in a macroscopic medium in a cavity or transmission line, the artifacts of the measurement fixture must be separated from the material properties by solving a relevant macroscopic boundary-value problem. At microwave and millimeter frequencies a low-loss macroscopic material can be made to resonate as a dielectric resonator. In such cases, if the appropriate boundary-value problem is solved, the intrinsic permittivity and permeability of the material can be extracted because the wavelengths are larger than the constituent molecule sizes, and as a result, the polarization vector is well defined. However, many modern applications are based on artificial structures that produce an EM response where the wavelength in the material is only slightly larger than the feature or inclusion size. In such cases, mapping the EM response onto a permittivity and permeability must be scrutinized. In general, the permittivity is well defined in materials where wave propagation through the material is not dominated by multiple scattering events.

## 5. Overview of the Dielectric Response to Applied Fields

### 5.1 Modeling Dielectric Response Upon Application of an External Field

Dielectric parameters play a critical role in many technological areas. These areas include electronics, microelectronics, remote sensing, radiometry, dielectric heating, and EM-assisted chemistry [20]. At RF frequencies dielectrics exhibit behavior that metals cannot achieve because dielectrics allow field penetration and can have low-to-medium loss characteristics.

Using dielectric spectroscopy as functions of both frequency and temperature we can obtain some, but not all of the information on a material’s molecular or lattice structure. For example, measurements of the polarization and conductivity indicate the polarizability and free charge of a material and polymer mobility of side chains can be studied with dielectric spectroscopy. Also, when a polymer approaches a glass transition temperature the relaxation times change abruptly. This is observable with dielectric spectroscopy. In addition, the loss peaks of many liquids change with temperature.

When an EM field is applied to a material, the atoms, molecules, free charge, and defects adjust positions. If the applied field is static, then the system will eventually reach an equilibrium state. However, if the applied field is time dependent then the material will continuously relax in the applied field, but with a time lag. The time lag is due to screening, coupling, friction, and inertia. An abundance of processes are occurring during relaxation, such as heat conversion processes, lattice-phonon, and photon phonon coupling. Dielectric relaxation can be a result of dipolar and induced polarization, lattice-phonon interactions, defect diffusion, higher multipole interactions, or the motion of free charges. Time-dependent fields produce nonequilibrium behavior in the materials due both to the heat generated in the process and the constant response to the applied field. However, for linear materials and time-harmonic fields, when the response is averaged over a cycle, if heating is appreciable, nonequilibrium effects such as entropy production relate more to temperature effects than the driving field stimulus. The dynamic readjustment of the molecules in response to the field is called *relaxation* and is distinct from resonance. For example, if a dc electric field is applied to a polarizable dielectric and then the field is suddenly turned off, then the dipoles will relax over a characteristic relaxation time into a more random state.

**Fig. 4:**
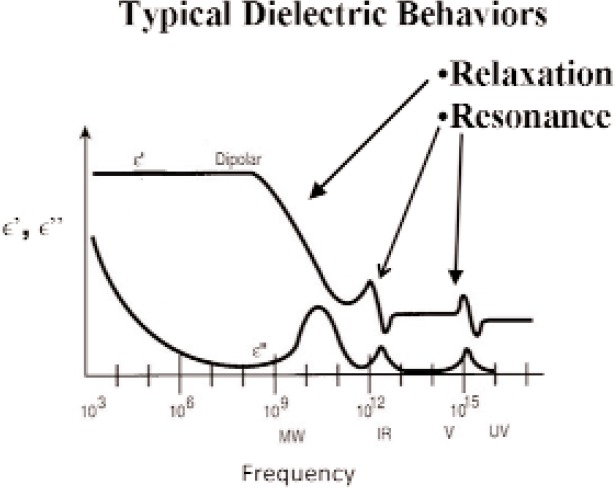
Broadband permittivity variation for materials [71].

**Fig. 5:**
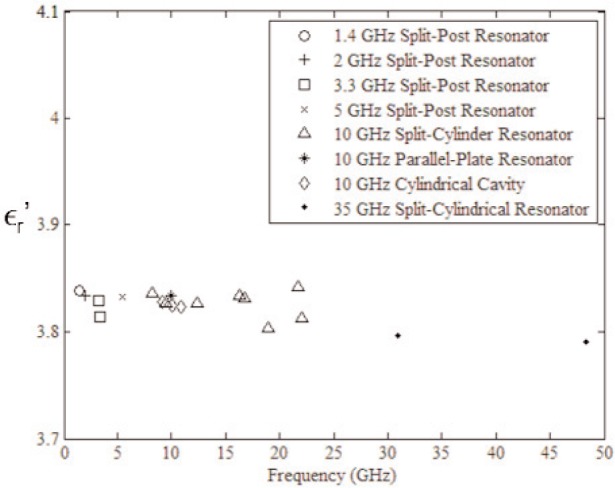
Typical frequency dependence of ε′*_r_* of low-loss fused silica as measured by many methods.

**Fig. 6:**
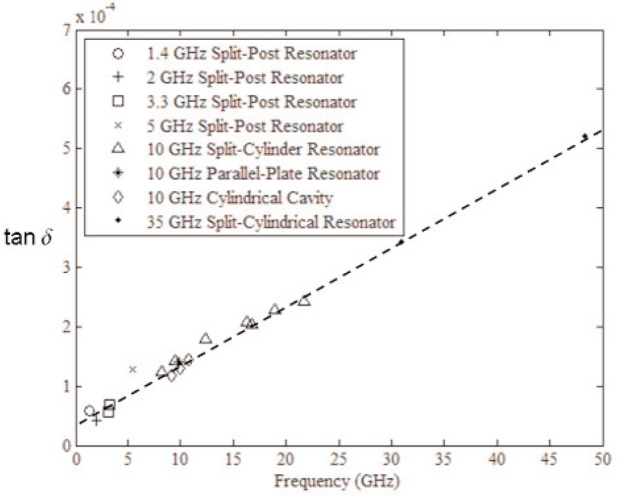
Typical frequency dependence of the loss tangent in low-loss materials such as fused silica.

The response of materials depends strongly on material composition and lattice structure. In many solids, such as solid polyethylene, the molecules are not able to appreciably rotate or polarize in response to applied fields, indicating a low permittivity and small dispersion. The degree of crystallinity, existence of permanent dipoles, dipole-constraining forces, mobility of free charge, and defects all contribute to dielectric response. Typical responses for high-loss and low-loss dielectrics are shown in Figs. 4, 5, and 6.

A material does not respond instantaneously to an applied field. As shown in Fig. 4, the real part of the permittivity is a monotonically decreasing function of frequency in the relaxation part of the spectrum, far away from intrinsic resonances. At low frequencies, the dipoles generally follow the field, but thermal agitation also tends to randomize the dipoles. As the frequency increases to the MMW band, the response to the driving field generally becomes more incoherent. At higher frequencies, in the terahertz or infrared spectrum, the dipoles may resonate, and therefore the permittivity rises until it becomes out of phase with the field and then drops. At RF frequencies, materials with low loss respond differently from materials with high loss (compare Fig. 4 for a high-loss material versus a low-loss material in Figs. 5 and 6). For some materials, at frequencies at the low to middle part of the THz band, $\varepsilon_{r}^{\prime }$ may start to contain some of the effects of resonances that occur at higher frequencies, and may start to slowly increase with frequency, until resonance, and then decreases again.

The local and applied fields in a dielectric are usually not the same. As the applied field interacts with a material it is modified by the fields of the molecules in the substance. Due to screening, the local electric field differs from the applied field and therefore theories of relaxation must model the local field (see Sec. 4.3).

Over the years, many models of polar and nonpolar-materials have been developed that use different approximations to the local field. The Clausius-Mossotti equation was developed for noninteracting, nonpolar molecules governed by the Lorentz equation for the internal field. This equation works well for nonpolar gases and liquids. Debye introduced a generalization of the Clausius-Mossotti equation for the case of polar molecules. Onsager developed an extension of Debye’s theory by including the reaction field and a more comprehensive local field expression [53]. For a dielectric composed of permanent dipoles, the polarization is written in terms of the local field as Eq. (42)

There are electronic, ionic, and permanent dipole polarizability contributions, so that ${\vec{\mu }}_{d}=(\alpha_{el}+\alpha_{ion}+\alpha_{perm})E_{l}$, α*_el_* = 4πε_0_*R*^3^/3, α*_ion_* = *e*^2^/*Yd*_0_. Here, *Y* is Young’s modulus, *R* is the radius of the ions, *d*_0_ is the equilibrium separation of the ions, and $\alpha_{perm}={\left|{\vec{\mu }}_{e}\right|}^{2}/3k_{B}T$, where ${\vec{\mu }}_{e}$ is the permanent dipole moment. There may also be a contribution to the polarizability due to excess charge at microscopic interfaces. Using the Lorentz expression for the local field, the polarization can be written as

(52) $$P=N\alpha \left(E+\frac{P}{3\varepsilon_{0}}\right),$$

or

(53) $$P=\frac{N\alpha E}{1-N\alpha /3\varepsilon_{0}}=\varepsilon_{0}(\varepsilon_{r}-1)E.$$

This is the Clausius-Mossotti relation that is commonly used to estimate the permittivity of nonpolar materials from atomic polarizabilities:

(54) $$\frac{\varepsilon_{r}-1}{\varepsilon_{r}+2}=\frac{N\alpha }{3\varepsilon_{0}},$$

or

(55) $$\varepsilon_{r}=\frac{3\varepsilon_{0}+2N\alpha }{3\varepsilon_{0}-N\alpha }.$$

The Clausius-Mossotti relation relates the permittivity to the polarizability. The polarizability is related to the vector dipole moment ${\vec{\mu }}_{d}$ of a molecule or atom and the local field **E***_l_*, ${\vec{\mu }}_{d}=\alpha E_{l}$. In principle, once the polarizability is determined for a group of molecules, then the permittivity of the ensemble can be calculated with the implicit assumption that there are many molecules located over the distance of a wavelength. Typical polarizabilities of atoms are between 0.1 and 100 Fm^2^ [72]. Polarizabilities of molecules can be higher than for atoms. The local field for a sphere is related to the polarization by Eq. (41).

A generalization of the Clausius-Mossotti equation to include a permanent moment ${\vec{\mu }}_{e}$ is summarized in what is called the Debye equation that is valid for gases and dilute solutions:

(56) $$\frac{\varepsilon_{r}-1}{\varepsilon_{r}+2}=\frac{N}{3\varepsilon_{0}}\left(\alpha +\frac{{\left|{\vec{\mu }}_{e}\right|}^{2}}{3k_{B}T}\right).$$

The Debye equation could be used to estimate the permittivity of a gas if both the polarizability and the dipole moment were known from experiment.

For a specific dipole immersed in an environment of surrounding dipoles, the dipole will tend to polarize the surrounding dipoles and thereby create a reaction field. Onsager included the effects of the reaction field into the local field and obtained the following relationship for the static field that, and unlike the Debye equation, can be used to model the dipole moment of some pure liquids:

(57) $${\left|{\vec{\mu }}_{e}\right|}^{2}=\frac{9k_{B}T\varepsilon_{0}}{N}\frac{\left(\varepsilon_{s}-\varepsilon_{\infty })(2\varepsilon_{s}-\varepsilon_{\infty }\right)}{\varepsilon_{s}{\left(\varepsilon_{\infty }+2\varepsilon_{0}\right)}^{2}},$$

where ε_∞_ is the optical limit of the permittivity. The Onsager equation is often used to calculate dipole moments of gases. Both atoms and molecules can polarize when immersed in a field. Note that Eq. (57) uses the permittivity of the liquid, which is a macroscopic quantity to estimate the microscopic dipole moment.

### 5.2 Dielectric Relaxation and Resonance

#### 5.2.1 Simple Differential Equations for Relaxation and Resonance

A very general, but simplistic equation, for modeling polarization response that depends on time is given by a harmonic-oscillator relation:

(58) $$\frac{1}{\omega_{0}^{2}}\frac{d^{2}P}{dt^{2}}+\tau \frac{dP}{dt}+P=\chi_{0}E,$$

where **P** is polarization, τ is the relaxation time, ω_0_ is the natural frequency $\left(\omega_{0}=\sqrt{k/m}\right)$, and χ_0_ = ε*_s_* − ε_∞_. Various special cases of Eq. (58) serve as simple, naive models of relaxation, resonance, and plasmonic response. The first term relates to the effects of inertia, the second to dissipation, the third to restoring forces, and the RHS represents the driving forces. A weakness of Eq.(58) is that the simple harmonic oscillator model assumes only a single relaxation time, and resonance frequency. This equation can be generalized to include interactions, (see Eq. (117)). In most materials, the molecules are coupled and have a broad range of relaxation frequencies that widens the dielectric response. For time-harmonic fields Eq. (58) is

(59) $$\tilde{P}(\omega )=\frac{\chi_{0}}{1-\frac{\omega^{2}}{\omega_{0}^{2}}+i\omega \tau }\tilde{E}(\omega ).$$

**Fig. 7:**
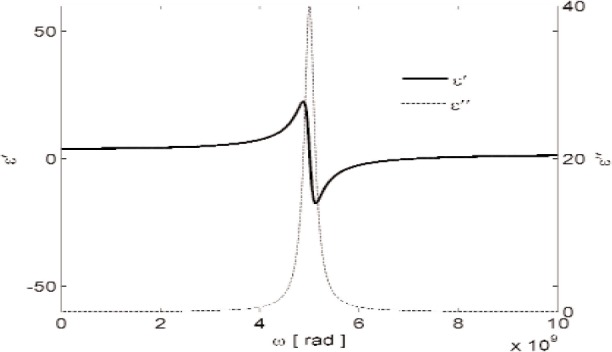
Theoretical resonance in the real part of the permittivity from Eq. (59) and associated loss factor.

A resonance example is shown in Fig. 7. Intrinsic material resonances in ionic solids can occur at high frequencies due to driving at phonon normal-mode frequencies and relate to the mass inertial aspect in $\omega_{0}=\sqrt{k/m}$ of the positive and negative charges of sublattices.

If we eliminate the inertial interaction when $\omega_{0}^{2}\gg \omega^{2}$, we have the time-domain Debye differential equation for pure relaxation:

(60) $$\tau \frac{dP}{dt}+P=\chi_{0}E.$$

For time-harmonic fields, the Debye response is

(61) $$\tilde{P}(\omega )=\frac{\chi_{0}}{1+i\omega \tau }\tilde{E}(\omega ).$$

Except for liquids like water, dielectrics rarely exhibit the response of Eq. (61) since there is no single relaxation time over RF frequencies.

We generally assume that dipoles reorient in an applied field in discrete jumps as the molecule makes transitions from one potential well minimum to another with the accompanied movement of a polaron or defect in the lattice. The Debye model of relaxation assumes that dipoles relax individually with no interaction between dipoles and with no inertia, but includes frictional forces. The real part of the permittivity for dipolar systems generally does not exhibit single-pole Debye response, but rather a power-law dependence. The origin of this difference can be attributed to many-body effects that tend to smear the response over a frequency band.

If we eliminate the restoring force term in Eq. (59), we have an equation of motion for charged plasmas,

(62) $$\frac{1}{\omega_{0}^{2}}\frac{d^{2}P}{dt^{2}}+\tau \frac{dP}{dt}=\chi_{0}E.$$

For time-harmonic fields, this becomes

(63) $$\tilde{P}(\omega )=\frac{\chi_{0}}{-\frac{\omega^{2}}{\omega_{0}^{2}}+i\omega \tau }\tilde{E}(\omega ).$$

#### 5.2.2 Modeling Relaxation in Dielectrics

The polarization of a material in an applied field depends on the permanent and induced dipole moments, the local field, and their ability to rotate with the field. Dielectric loss in polar materials is due primarily to the friction caused by rotation, free charge movement, and out-of-phase dipole coupling. Losses in nonpolar materials originate mainly from the interaction with neighboring permanent and induced dipoles, intrinsic photon-phonon interactions with the EM field, and extrinsic loss mechanisms caused by defects, dislocations, and grain structure. Loss in many high-purity crystals is primarily intrinsic in that a crystal will vibrate nearly harmonically; however, anharmonic coupling to the electric field and the presence of defects modifies this behavior. The anharmonic interaction allows photon-phonon interaction and thereby introduces loss [73]. High-purity centro-symmetric dielectric crystals, that is, crystals with reflection symmetry, such as crystalline sapphire, strontium titanate, or quartz, have generally been found to have lower loss than crystals with noncentrosymmetry [74].

A transient current may be induced if an electric field is applied, removed, or heated. This can be related to the dielectric response. The depolarization current for many lossy disordered solids is nonexponential and, at time scales short relative to the relaxation time of the media, can satisfy a power law of the form [75, 76]

(64) $$I(t)\propto t^{-n},$$

and satisfy a power law at long times of the form

(65) $$I(t)\propto t^{-(1+m)},$$

where 0 < *n*, *m* < 1. In this model, a short time scale corresponds to frequencies in the microwave region (τ ∝ 1/*f* < 1 × 10^−9^s) and long relaxation times refer to frequencies less than 10 kHz (τ ∝ 1/*f* < 1 × 10^−4^s). In order to satisfy theoretical constraints at very short periods the current must depart from Eq. (64). There are exceptions to the behavior given in Eqs. (64) and (65) in dipolar glasses, polycrystalline materials, and other materials [77]. The susceptibility of many lossy disordered solids typically behave at high frequencies as a power law

(66) $$\chi^{\prime }(\omega )\propto \chi^{\prime \prime }(\omega )\propto \omega^{\left(n-1\right)}.$$

This implies χ″/χ′ is independent of frequency. On the other hand, measurements of many ceramics, glasses, and polymers exhibit a loss tangent that increases approximately linearly with frequency as shown in Fig. 6.

Dissado and Hill conclude that nonexponential relaxation is related to cluster response [75]. In their model, molecules within a correlated region react to the applied field with a time delay. The crux of this approach is that in most condensed-matter systems the relaxation is due not to independently relaxing dipoles, but rather that the relaxation of a single dipole depends on the state of other dipoles in a cluster. Therefore their model includes dipole-dipole coupling. This theory of disordered solids is based on charge hopping and dipolar transitions within regions surrounding a defect and between clusters [75]. The effect is to spread out the response over time and therefore to produce nonexponential behavior. Dissado and Hill developed a representation of a correlation function that includes cluster interaction. According to this theory, the time-domain response for short time scales is Gaussian $e^{-t^{2}/\tau^{2}}$.

At longer periods there are intra-cluster transitions that follow a power law of the form *t*^−^*^n^*. At still longer periods there are inter-cluster transitions with a Debye-type response *e*^−^*^t/τ^*, and finally at very long periods there is response of the form *t*^−^*^m^*^−1^ [75].

Jonscher, Dissado, and Hill have developed theories of relaxation based on fractal self-similarity [78, 79]. Jonscher’s approach is based on a screened-hopping model where response is modified due to many-body charge screening [80]. In the limit of weak screening, the Debye model is recovered.

Nonexponential response has been obtained with many models. In any materials where the dipoles do not rotate independently, the relaxation is nonexponential. Nonexponential response has also been reproduced in computer simulations for chains of dipoles by means of a correlation-function approach with coupled rate Eqs. [81–83].

Note that nonexponential time-domain response is actually required for over some bands in order to have a causal-function response over all frequencies. This is a consequence of the Paley-Wiener theorem [84]. According to this theorem, the correlation or decay function cannot be a purely damped exponential function for large times. If *C*(*t*) is the decay function then

(67) $$\int_{-\infty }^{\infty }d\tau \frac{|\log|C(\tau )∥}{1+\tau^{2}},$$

must be finite. This requires the decay function to vanish less fast than a pure exponential at large times, *C*(*t*) ≈ exp (− *ct^q^*) where *q* < 1 and *c* is a constant. We can show that at short times, decay occurs faster than exponential [85].

Nigmatullian et al. [86, 87] used the Mori-Zwanzig formalism to express the permittivity in a very general form:

(68) $$\varepsilon (i\omega )=\varepsilon_{\infty }+\frac{\varepsilon_{s}-\varepsilon_{\infty }}{1+R_{\pm }(i\omega )},$$

and concluded that for most disordered materials, the response is similar to that of a distributed circuit with $R_{\pm }(i\omega )={\left[{\left(i\omega \tau_{1}\right)}^{\pm \nu_{1}}+{\left(i\omega \tau_{2}\right)}^{\pm \nu_{2}}\right]}^{\pm }$, where ν*_i_* are constants determined by numerical fits. In the formulation of Baker-Jarvis et al. [88], *R*_±_ corresponds to the complex relaxation times τ(ω) as *R*_+_(*i*ω) = *i*ωτ(ω) (see Sec. 11). A (*i*ωτ)^(^*^n^*^−1)^ frequency dependence of the complex relaxation periods corresponds to a impulse-response function of the form *t*^−^*^n^*.

In addition, in analyzing dielectric data the electric modulus approach is sometimes used where $M(\omega )=M^{\prime }(\omega )+iM^{\prime \prime }(\omega )=1/\varepsilon_{r}=\varepsilon_{r}^{\prime }/(\varepsilon_{r}^{\prime }{}^{2}+\varepsilon_{r}^{\prime \prime }{}^{2})+i\varepsilon_{r}^{\prime \prime }/(\varepsilon_{r}^{\prime }{}^{2}+\varepsilon_{r}^{\prime \prime }{}^{2})$.

Dielectric relaxation has also been described by Kubo’s linear-response theory that is based on correlation functions. This is an example of a relaxation theory derived from Liouville’s equation. The main difficulty with these approaches is that the correlation functions are difficult to approximate to highlight the essential physics, and gross approximations are usually made in numerical calculations. The linear expansion of the probability-density function in Kubo’s theory also limits its usefulness for highly nonequilibrium problems. Baker-Jarvis et al. have recently used a statistical-mechanical projection-operator method developed by Zwanzig and Robertson [89] to model dielectric and magnetic relaxation response and the associated entropy production [19, 40, 41, 43, 44].

## 6. The Distribution of Relaxation Times (DRT) Model for Homogeneous Materials

There are many models used to fit measured frequency-dependent dielectric relaxation data for homogeneous materials. These models are usually general enough to fit many types of response. When dealing with heterogeneous materials, mixture equations are commonly used (Sec. 22). The DRT model is restricted to relaxation, and it assumes there is a probability distribution *y*(*t*) that underpins the relaxation response with a relaxation time τ. In this model, the permittivity can be written as

(69) $$\varepsilon (\omega )=\varepsilon_{\infty }+(\varepsilon_{s}-\varepsilon_{\infty })\int_{0}^{\infty }\frac{y(\tau )}{1+(i\omega \tau )}d\tau ,$$

where

(70) $$\int_{0}^{\infty }y(\tau )d\tau =1.$$

Note that DRT is a single-pole model and cannot be used for resonances. We see that in the DRT, Debye relaxations are weighted by a probability-density function. Equation (69) can be inverted by the Laplace transform as shown in the Appendix of Böttcher [53].

The DRT approach is sufficiently general that most causal, relaxation dielectric-response phenomena can be described by the model for Debye and power-law response. In the DRT the slope of $\varepsilon_{r}^{\prime }(\omega )$ is always negative [90]. This is consistent with causality. It also indicates that the model is only valid for relaxation and not resonance. Around resonance $\varepsilon_{r}^{\prime }(\omega )$ can increase with frequency and become negative as indicated in Fig. 7.

Equation (69) can fit the relaxation response of many dielectrics because the Debye equation originates from a rate equation based on thermodynamics containing the essential physics, and Eq. (69) is a distribution of Debye relaxations. The DRT then extends this into a multi-relaxation period rate equation. We consider various special cases of Eq. (69) below. For other special cases please see Böttcher [53]. In any complex dielectric material, we would expect there would be a broadening of relaxation times due to heterogeneity of the molecular response, and in this context the DRT model makes sense. This approach is often criticized, because it is not always possible obtain a physical interpretation of the distribution function [75].

### 6.1 Debye Model

The simplest case of the distribution function in Eq. (69) is an uncorrelated approximation where

(71) y(t)=δ(t−τ),

which yields the Debye response

(72) $$\varepsilon =\varepsilon_{\infty }+\frac{\left(\varepsilon_{s}-\varepsilon_{\infty }\right)}{1+i\omega \tau }.$$

In this case, the pulse response function is

(73) $$f_{p}(t)=\exp(-t/\tau )/\tau .$$

In terms of components,

(74) $$\varepsilon_{r}^{\prime }(\omega )=\varepsilon_{\infty }+\frac{\varepsilon_{s}-\varepsilon_{\infty }}{1+\omega^{2}\tau^{2}},$$

and

(75) $$\varepsilon_{r}^{\prime \prime }(\omega )=\frac{(\varepsilon_{s}-\varepsilon_{\infty })\omega \tau }{1+\omega^{2}\tau^{2}}.$$

If ωτ is eliminated in the Debye model, and the equations for $\varepsilon_{r}^{\prime }(\omega )$ and $\varepsilon_{r}^{\prime \prime }(\omega )$ are plotted against each other, we obtain the equation for a circle:

(76) $${\left[\varepsilon^{\prime }(\omega )-\frac{\varepsilon_{s}-\varepsilon_{\infty }}{2}\right]}^{2}+{\varepsilon^{\prime \prime }}^{2}(\omega )={\left[\frac{\varepsilon_{s}-\varepsilon_{\infty }}{2}\right]}^{2}.$$

The center of the circle is on the horizontal axis.

The reasons why the Debye equation is a paradigm in dielectric relaxation theory is because it is simple and contains the essential physics and thermodynamics in relaxation. That is, it models idealized relaxation, and it yields predictions on the temperature dependence of the relaxation time τ = *A* exp (*E_a_*/*RT*), where *E_a_* is the activation energy.

### 6.2 Cole-Cole Model

The Cole-Cole model has been found useful for modeling many liquids, semisolids, and other materials [53]. In this case,

(77) $$y(\tau )=\frac{1}{2\pi }\frac{\sin\pi \alpha }{\cosh[(1-\alpha )\text{In}\;\tau /\tau_{0}-\cos\;\pi \alpha ]}$$

and

(78) $$\varepsilon (\omega )=\varepsilon_{\infty }+\frac{\varepsilon_{s}-\varepsilon_{\infty }}{1+{\left(i\omega \tau_{0}\right)}^{1-\alpha }},$$

where α < 1. The pulse response function is $f_{p}(t)=(1/\tau_{0}){\sum_{m=0}^{\infty }{\left(-1\right)}^{m}/\Gamma ((m+1)(1-\alpha ))\left(t/\tau_{0}\right)}^{m(1-\alpha )-\alpha }$. The real and imaginary parts of the permittivity can be separated into

(79) $$\varepsilon_{r}^{\prime }(\omega )=\varepsilon_{\infty }+(\varepsilon_{s}-\varepsilon_{\infty })\frac{1+{\left(\omega \tau_{0}\right)}^{1-\alpha }\sin(\pi \alpha /2)}{1+{\left(\omega \tau_{0}\right)}^{2(1-\alpha )}+2{\left(\omega \tau_{0}\right)}^{1-\alpha }\sin(\pi \alpha /2)},$$

(80) $$\varepsilon_{r}^{\prime \prime }(\omega )=(\varepsilon_{s}-\varepsilon_{\infty })\frac{{\left(\omega \tau_{0}\right)}^{1-\alpha }\cos(\pi \alpha /2)}{1+{\left(\omega \tau_{0}\right)}^{2(1-\alpha )}+2{\left(\omega \tau_{0}\right)}^{1-\alpha }\sin(\pi \alpha /2)}.$$

A plot of $\varepsilon_{r}^{\prime }$ versus $\varepsilon_{r}^{\prime \prime }(\omega )$ yields a circle, where the center is below the vertical axis.

### 6.3 Cole-Davidson Model

The Cole-Davidson model has also been found useful for modeling many liquids, semisolids, and other materials [53]. If we consider the case τ ≤ τ_0_:

(81) $$y(\tau )=\frac{1}{\pi }{\left[\frac{\tau }{\tau_{0}-\tau }\right]}^{\beta }\sin\pi \beta$$

and zero otherwise. The permittivity is

(82) $$\varepsilon_{r}(\omega )=\varepsilon_{\infty }+\frac{\left(\varepsilon_{s}-\varepsilon_{\infty }\right)}{{\left(1+i\omega \tau_{0}\right)}^{\beta }},$$

where β < 1. The pulse response function is

(83) $$f_{p}(t)=(1/\tau_{0}\Gamma (\beta )){\left(t/\tau_{0}\right)}^{\beta -1}\exp(-t/\tau_{0}).$$

The real and imaginary parts of the permittivity can be separated into

(84) $$\varepsilon_{r}^{\prime }(\omega )=\varepsilon_{\infty }+(\varepsilon_{s}-\varepsilon_{\infty }){\left(1+\omega^{2}\tau_{0}^{2}\right)}^{-\beta /2}\cos(\beta Arg[1+i\omega \tau_{0}]),$$

(85) $$\varepsilon_{r}^{\prime \prime }(\omega )=(\varepsilon_{s}-\varepsilon_{\infty }){\left(1+\omega^{2}\tau_{0}^{2}\right)}^{-\beta /2}\sin(\beta Arg[1+i\omega \tau_{0}]).$$

The plot of $\varepsilon_{r}^{\prime }(\omega )$ versus $\varepsilon_{r}^{\prime \prime }(\omega )$ maps out a skewed arc rather than a circle.

### 6.4 Havrilak-Negami Model

The Havrilak-Negami distribution has two parameters to fit data and is very general. It can be used to fit the response of many liquids and non-Debye solid materials [53]. In the special case α = 0 it reverts to the Cole-Davidson model. The distribution function is

(86) $$y(\tau )=\frac{1}{\pi }\frac{{\left(\frac{\tau }{\tau_{0}}\right)}^{\beta (1-\alpha )}\sin\beta \theta }{{\left[\left(\frac{\tau }{\tau_{0}}\right)\right]}^{2(1-\alpha )}+2{\left(\frac{\tau }{\tau_{0}}\right)}^{\left(1-\alpha \right)}{\cos(\pi (1-\alpha ))+1]}^{\beta /2}},$$

and θ = tan^−1^{sin π(1 − α)/(τ/τ_0_ + cos π(1 – α))}

(87) $$\varepsilon_{r}(\omega )=\varepsilon_{\infty }+\frac{\left(\varepsilon_{s}-\varepsilon_{\infty }\right)}{{\left(1+{\left(i\omega \tau_{0}\right)}^{1-\alpha }\right)}^{\beta }},$$

where 0 < α ≤ 1 and 0 < β ≤ 1, and

(88) $$\begin{array}{l} \varepsilon_{r}^{\prime }(\omega )=\varepsilon_{\infty }+(\varepsilon_{s}-\varepsilon_{\infty })\\ \;\times \frac{\cos\beta \theta }{{\left[1+2{\left(\omega \tau_{0}\right)}^{\left(1-\alpha \right)}\sin(\frac{1}{2}\pi \alpha )+{\left(\omega \tau_{0}\right)}^{2(1-\alpha )})\right]}^{\beta /2}}, \end{array}$$

(89) $$\begin{array}{l} \varepsilon_{r}^{\prime \prime }(\omega )=\varepsilon_{\infty }+(\varepsilon_{s}-\varepsilon_{\infty })\\ \;\times \frac{\sin\beta \theta }{{\left[1+2{\left(\omega \tau_{0}\right)}^{\left(1-\alpha \right)}\sin(\frac{1}{2}\pi \alpha )+{\left(\omega \tau_{0}\right)}^{2(1-\alpha )})\right]}^{\beta /2}}. \end{array}$$

## 7. Loss and Conductivity

Loss originates from the conversion of EM field energy into heat and radiation through photon-phonon interactions. In dielectrics the heating is caused by the transformation of electromagnetic energy into lattice kinetic energy, which is seen as frictional forces on dipoles and the motion and resulting friction of free charges in materials. Major mechanisms of conduction in dielectrics in the RF band are ionic or electrolytic migration of free ions, impurities or vacancies, electrophoretic migration of charged molecules, and electronic conduction of semi-free electrons that originate from jump processes of polarons. At low frequencies, dipoles can respond to the changes in the applied field, so dielectric losses usually are low and the stored energy is high, but as the frequency increases, the dipole response tends to fall behind the applied field and, therefore, the loss usually increases and the stored energy decreases. This is related to the phasing between the current and voltage waves, in analogy to the heating an electric motor encounters when the phase between the voltage and current changes.

Ionic conduction in insulating dielectrics is due to the migration of charged ions. The migration takes place through tunneling or jumps induced by the applied field, or by slow migration under the applied field. In solid polymers it may proceed by jumps from one vacancy to another or by electronic conduction. In oxide glasses it is the movement of positively charged alkali ions in the applied field. In many materials, the dielectric losses originate in vacancy-vacancy and vacancy-impurity relaxations.

At high frequencies, lossy semiconductors, superconductors, and metals have a complex free-charge ac conductivity that is explained by the Drude model. This can cause the effective permittivity to become negative [27]. To understand this, consider Maxwell’s equation,

(90) $$\frac{\partial D}{\partial t}+J=\nabla \times H.$$

We can define an effective charge current as

(91) $$J_{eff}(t)=\int_{-\infty }^{t}\sigma (t-\tau )E(\tau )d\tau +\frac{\partial D}{\partial t},$$

or for time-harmonic fields

(92) $${\tilde{J}}_{eff}(\omega )=\sigma (\omega )\tilde{E}(\omega )+i\omega \tilde{D}(\omega ).$$

Combining ac **J** with the displacement field produces an effective real part of the permittivity that can be negative over a region of frequencies. For example in plasmas and superconductors, the effective conductivity satisfies $i\omega \tilde{D}\;(\omega )+\tilde{J}\;(\omega )=[i\omega (\varepsilon^{\prime }(\omega )-i\varepsilon^{\prime \prime }(\omega ))+\sigma^{\prime }(\omega )-i\sigma^{\prime \prime }(\omega )]\tilde{E}$, yielding

(93) $$\varepsilon_{eff(r)}(\omega )=\varepsilon_{r}^{\prime }(\omega )-\frac{\sigma^{\prime \prime }(\omega )}{\varepsilon_{0}\omega }-i\left(\varepsilon_{r}^{\prime \prime }(\omega )+\frac{\sigma^{\prime }(\omega )}{\varepsilon_{0}\omega }\right),$$

where σ′ ≈ σ*_dc_* and σ″ relates to the reactive part of the surface impedance. A large σ″ can produce a negative real part of the total permittivity such as what occurs in superconductors [91].

A total conductivity has been used in the literature to model either the ac effects of the free charge and partially bound free charge in hopping and tunneling conduction, or as another way of re-expressing the complex permittivity. Because some charge is only partially bound, the distinction between conductivity and permittivity can, at times, get blurred. This blurring points out the mesoscopic property of the permittivity. Most models of ac conductivity are based on charged particles in potential wells where energy fluctuations determine whether the particle can surmount a potential barrier and thereby contribute to the conductivity. In conducting liquids, human tissue, and water-based semisolids the conductivity is generally flat with increasing frequency until megahertz frequencies, and then it increases, often in a nearly linear fashion.

There are a number of distinct models for σ*_tot_*. The Drude model of the complex conductivity of electrons or ions in a metal is approximately modeled as

(94) $$\sigma_{tot}=Ne^{2}/(m(\gamma_{0}+i\omega ))=\sigma^{\prime }-i\sigma^{\prime \prime },$$

where γ_0_ is the collision frequency, *N* is the electron density, *m* is the ion mass, and *e* is the electronic charge [27]. Note that the dc conductivity is σ*_dc_* = *Ne*^2^/*m*γ_0_. The net dielectric response is a sum of the dipolar contribution and that due to the ions, where $\varepsilon_{eff}^{\prime }=\varepsilon_{d}^{\prime }-Ne^{2}/m(\gamma_{0}^{2}+\omega^{2})$ and $\varepsilon^{\prime \prime }(\omega )=Ne^{2}\gamma_{0}/m\omega (\gamma_{0}^{2}+\omega^{2})+\varepsilon_{d}^{\prime \prime }(\omega )$. Therefore, for metals, the real part of the permittivity is negative for frequencies near the plasma frequency, $\omega_{p}=\sqrt{Ne^{2}/\varepsilon_{0}m}$. The plasma frequency in metals is usually well above 100 GHz. The conductivity is thermally active and can be modeled for some ionic materials as [92]

(95) $$\sigma_{dc}=\frac{n_{c}e^{2}b^{2}\nu_{0}}{k_{B}T}\exp(-\frac{\Delta G}{k_{B}T}),$$

where *n_c_* is the ion vacancy, *b* is the ion jump distance, ν_0_ is a characteristic ion frequency, and Δ*G* is the Gibb’s free energy.

For plasmas at high frequencies

(96) $$\varepsilon^{\prime }(\omega )\to \varepsilon_{0}\left(1-\frac{\omega_{p}^{2}}{\gamma_{0}^{2}+\omega^{2}}\right).$$

For disordered solids, where hopping and tunneling conduction takes place with a relaxation time τ*_e_*, the ac conductivity can be expressed as [93, 94]

(97) $$\begin{array}{l} \sigma_{tot}(\omega )=\sigma_{0}i\omega \tau_{e}/\ln(1+i\omega \tau_{e})\\ \;=\sigma_{0}[\frac{\omega \tau_{e}\arctan(\omega t_{e})}{\frac{1}{4}\ln^{2}(1+\omega^{2}\tau_{e}^{2})+\arctan^{2}(\omega \tau_{e})}\\ \;-i\frac{\omega \tau_{e}\ln\left(1+{\left(\omega \tau_{e}\right)}^{2}\right)}{\frac{1}{2}\ln^{2}(1+\omega^{2}\tau_{e}^{2})+2\arctan^{2}(\omega \tau_{e})}]. \end{array}$$

## 8. Double Layers and Conducting Materials Near Metal Interfaces

**Fig. 8:**
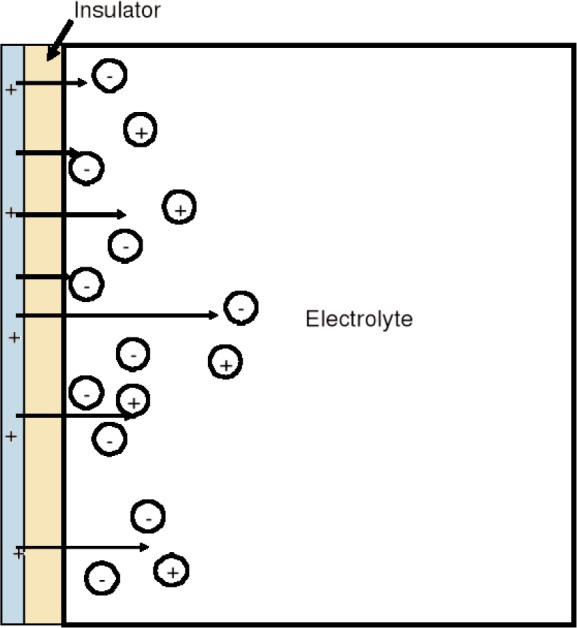
Electrolyte charges near an electrode.

Conducting and semiconducting dielectric materials at interfaces or metallic contacts can be influenced by the effects of double layers. Measurements on conducting liquids are complicated by the effects of electrode polarization, which are the direct result of the double layers [95]. Double layers and electrode polarization are due to the build up of anions and cations at the interface of electrodes and conducting materials, as shown in Fig. 8. Modeling ionic solutions near electrodes is complicated, because the charge is mobile and depends on the potential.

Two conducting dissimilar materials can have different electronic affinities. When these dissimilar materials are in contact, a potential gradient frequently develops between the materials. As a result an electrical double layer forms at a material interface. This interface could be between liquid and metal electrodes or the layer between a biomolecule and a liquid. The potential difference will attract ions of opposite charge to the surface and repel like charges. For a double layer, the charge density depends nonlinearly on the applied potential and is modeled at low frequencies by the Poisson-Boltzmann equation for the potential [96, 97] (∇^2^ψ = −ρ(ψ)/ε). The potential decreases roughly exponentially from the surface as ψ(*x*) = ψ_0_ exp (–*x*/λ*_D_*), where λ*_D_* is the Debye screening length or skin depth. The region near the electrode consists of the Stern layer and a diffuse region beyond the Stern layer where the potential decays less rapidly. It is known that the Poisson-Boltzmann equation is of limited use for calculating the potential around many biomolecules due to molecular interactions and the effects of excluded volume [97].

At the interface of conductive materials and electrodes, electrode polarization produces a capacitive double-layer region in series with the specimen under test. The presence of electrode polarization results in $\varepsilon_{eff}^{\prime }$ being much greater than the value for the liquid by itself. Because the electrode capacitance is not a property of the material under test, but rather the interface, it can be treated as a systematic uncertainty and methods to remove it from the measurement can be applied. Double layers also form at the metal interface with semiconducting materials where the conductivity is a function of applied voltage.

The effects of electrode polarization can strongly affect dielectric measurements up to around 1 MHz, but the effects can be measurable up into the low gigahertz frequencies. Any electrode influencing the calculated permittivity should be treated as a systematic source of uncertainty. Alternatively, the permittivity with the electrode effects could be called the effective permittivity.

The effects of electrode polarization capacitance as commonly analyzed with the following model [98]

(98) $$C=C_{s}+\frac{1}{\omega^{2}R^{2}C_{p}},$$

(99) $$R=R_{s}(1+\omega^{2}R^{2}C^{2})+R_{p},$$

where *C* and *R* are the measured capacitance and resistance, *C_p_* and *R_p_* are the electrode double-layer capacitance and resistance, and *C_s_* and *R_s_* are the specimen capacitance and resistance. A way to partially eliminate electrode polarization is to measure the capacitances *C*_1_ and *C*_2_ and resistances *R*_1_ and *R*_2_ at two separations *d*_1_ and *d*_2_. Because *C_p_* is the same for each measurement and *C_s_* can be scaled as *C_s_*_2_ = (*d*_1_/*d*_2_)*C_s_*_1_, we can obtain the specimen capacitance. Another way of minimizing the effects of electrode polarization is to coat the capacitor plates with platinum black [99]. This lessens the influence of electrode polarization by decreasing the second term on the right hand side of Eq. (98). However, both the coating and two-distance methods schemes do not completely solve this problem. For biological liquids, often the buffer solution is first measured by itself and then again with the added biological material and the difference between the measurements is reported.

**Fig. 9:**
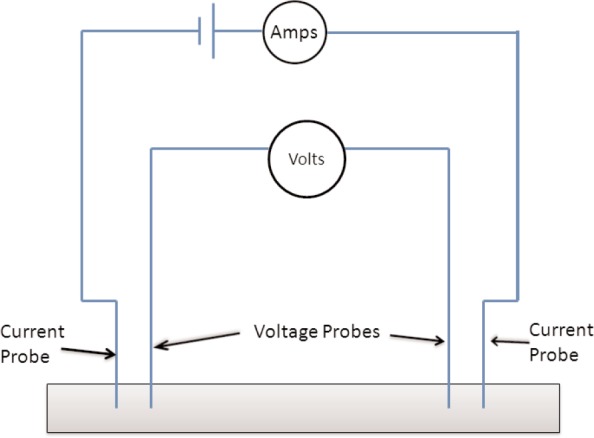
Four probe measurement.

For dielectric measurements, probably the best approach is to bypass much of the electrode-polarization problem altogether and use a four-probe capacitor system as shown in Fig. 9. The four-probe capacitance technique overcomes electrode problems by measuring the voltage drop away from the plates and thereby avoiding the double layer [100].

## 9. Relationships of the Permittivity Components: Causality and Kramers-Kronig Equations

Kramers-Kronig relations relate the real and imaginary parts of the permittivity. These equations are a result of causality and analytic functions. There are many forms of the Kramers-Kronig conditions [101], below are standard relationships

(100) $$\varepsilon_{r}^{\prime }(\omega_{0})-\varepsilon_{\infty }=\frac{2}{\pi }\int_{0}^{\infty }\frac{\varepsilon_{r}^{\prime \prime }(\omega )\omega -\varepsilon_{r}^{\prime \prime }(\omega_{0})\omega_{0}}{\omega^{2}-\omega_{0}^{2}}d\omega ,$$

(101) $$\varepsilon_{r}^{\prime \prime }(\omega_{0})=\frac{2\omega_{0}}{\pi }\int_{0}^{\infty }\frac{\left[\varepsilon_{r}^{\prime }(\omega )-\varepsilon^{\prime }(\omega_{0})\right]}{\omega^{2}-\omega_{0}^{2}}d\omega .$$

For example, if we neglect any dc conductivity, the dc permittivity must satisfy

(102) $$\varepsilon_{s}-\varepsilon_{\infty }=\frac{2}{\pi }\int_{0}^{\infty }\frac{\varepsilon^{\prime \prime }(x)}{x}dx.$$

We should note that σ*_dc_* is not causally related to the permittivity and, therefore, before Kramers-Kronig analysis is performed, the contribution of conductivity to the loss should be subtracted.

As a consequence of causality, the permittivity satisfies the condition ε^*^(ω) = ε(−ω). Causality and second law of thermodynamics requires that when the response is averaged over a cycle, for a passive system ε″(ω) > 0 and *μ*″(ω) > 0. However, ε′(ω) or *μ′*(ω) can be greater or less than zero. Also, the real part of the characteristic impedance must be greater than zero.

## 10. Static and Dipolar Polarization

### 10.1 Static Polarization

The total kinetic energy (*K*) plus potential energy of a dipole in a static applied field is approximately

(103) $$U=K-{\vec{\mu }}_{d}\cdot E.$$

The probability that a dipole is aligned at angle θ to the directing electric field is

(104) $$p(\theta )=A\exp(\frac{{\vec{\mu }}_{e}\cdot E}{3k_{B}T}).$$

The average moment for *N* dipoles is therefore

(105) $$P=N|{\vec{\mu }}_{e}|\frac{\int_{0}^{\pi }p(\theta )\sin\theta \cos\theta d\theta }{\int_{0}^{\pi }p(\theta )\sin\theta d\theta },$$

or

(106) $$P=N|{\vec{\mu }}_{e}|\left[\coth\frac{\left|{\vec{\mu }}_{e}||E\right|}{k_{B}T}-\frac{k_{B}T}{\left|{\vec{\mu }}_{e}||E\right|}\right]=N|{\vec{\mu }}_{e}|L\left(\frac{\left|{\vec{\mu }}_{e}||E\right|}{k_{B}T}\right),$$

where *L*(x) = coth(*x*) − 1/x ≈ *x*/3 − *x*^3^/45 + *x*^5^/945… is the Langevin function. At high temperatures or weak fields, the Langevin function is approximated as

(107) $$P=N|{\vec{\mu }}_{e}|L\left(\frac{\left|{\vec{\mu }}_{e}||E\right|}{k_{B}T}\right)\approx N\frac{|\mu_{e}^{2}|E}{3k_{B}T},$$

and in the approximation we assume $|{\vec{\mu }}_{e}||E|/k_{B}T<0.1$. Note that the model shows that the polarizing effect of the applied field affects < cosθ >, and there is a lesser effect on the direction of the individual dipole moments. At room temperature this corresponds to an electric field of about 3 × 10^7^(V/m), which is a very strong field. In intense fields or low temperatures, higher-order terms in the Langevin function must be included [53].

Using a similar analysis, the magnetic moment for noninteracting paramagnetic materials has the same form as Eq. (107)

(108) $$<M_{h}>=N\frac{|\mu_{h}^{2}|\mu_{0}H}{3k_{B}T}.$$

### 10.2 Deriving Relaxation Equations by Analyzing Dipolar Orientation in an Applied Field

Upon application of an electric field, dipole moments, impurities, and vacancies can change positions in the lattice potential wells. This is the origin of rotation, conduction, and jump reorientation [53, 102].

Consider the density of *N±* molecules where there are *N*_±_ dipole moments that are aligned either parallel (+) or antiparallel (−) to the applied field. The time evolution of the numbers of dipoles is described by the number of dipoles flipping one direction minus the number flipping the other direction characterized by the transition rates ν_±_, where ν_+_ denotes the rate of going from a + state to a − state

(109) $$\frac{dN_{\pm }}{dt}=\nu_{\mp }N_{\mp }-\nu_{\pm }N_{\pm }.$$

In equilibrium and in the absence of an electric field, the number of transitions in either direction is the same so that ν_+_*N*_+_ = ν_−_*N*_−_, where *N*_+_ + *N*_−_ = *N*. In an electric field, the transition rates are given by

(110) $$\nu_{\pm }=\nu_{\infty }\exp(-(U_{0}/k_{B}T\pm N\mu_{e}E/3k_{B}T)),$$

where ν_∞_ is the maximum transition rate and the factor 3 is related to isotropic polarization $pE\to |{\vec{\mu }}_{e}\cdot E|/3$. At high temperatures and ν_0_ = ν_∞_ exp (− *U*_0_/*k_B_T*)

(111) $$\nu_{\pm }\approx \nu_{0}\left(1\mp \frac{\mu_{e}E}{3k_{B}T}\right).$$

Therefore, for molecules that each have a permanent electric dipole moment ${\vec{\mu }}_{e}$, the net polarization is $P(t)=|{\vec{\mu }}_{e}|(N_{+}-N_{-})=|{\vec{\mu }}_{e}|(2N_{+}-N)$, and

(112) $$\frac{dP}{dt}+2\nu_{0}P=\frac{2\nu_{0}{\left|{\vec{\mu }}_{e}\right|}^{2}NE}{3k_{B}T}.$$

The relaxation time is τ = 1/2ν_0_ = (1/2ν_∞_) exp (*U*_0_/*k_B_T*). In this model the susceptibility is

(113) $$\chi_{s}=\frac{N\mu_{e}^{2}}{3k_{B}T}.$$

Therefore Eq. (112) reduces to the Debye equation

(114) $$\frac{dP}{dt}+P/\tau =\chi_{s}E/\tau .$$

Note that such a simple model can describe to a remarkable degree the polarization and yields a relaxation time with a reasonable dependence on temperature. This indicates the basic physics is correct.

## 11. Relaxation Times

### 11.1 Background

When a field is applied to a material, the material responds by re-arranging charge, causing spin precession, and currents. The characteristic time it takes for the response is called a relaxation time. Relaxation times are parameters used to characterize both dielectric and magnetic materials. Dielectric relaxation times are correlated with mechanical relaxation times [103]. Magnetic relaxation in NMR and ESR is modeled by spin-spin (*T*_2_) and spin-lattice (*T*_1_) relaxation times.

In the literature, dielectric relaxation times have been identified for molecules and bulk materials. The first is a single molecule relaxation time τ*_s_* and the other is a Debye mesoscopic relaxation time τ*_D_*. For magnetic nanoparticles in a fluid, where the magnetic moment is locked in place in the lattice, the Brownian time constant is defined as τ*_B_* = 3ν*V_H_*/*k_B_T*, where ν is the fluid viscosity and *V_H_* is the hydrodynamic volume of the particle [104]. The Neel relaxation time is for crystals where the magnetic moment is free to rotate in the field. Dielectric relaxation times are related to how the dipole moments and charge are constrained by the surrounding material. The characteristic relaxation time for a polarized material that was in an applied field at *t* = 0 to decay to a steady state is related to the coupling between dipoles and details of the lattice. At high frequencies, the electric response of a material lags behind the applied field when the field changes faster than the relaxation response of the molecules. This lag is due to long and short-range forces and inertia. The characteristic Debye relaxation time τ*_D_* can be obtained from the maximum of the loss peak in Eq. (61). Relaxation times are usually defined through the decay of the impulse-response function that is approximated by a Debye response exp (−*t*/τ). Debye used Onsager’s cavity model to show that τ*_D_*/τ*_s_* = (ε*_s_* + 2) / (ε_∞_ + 2) [105, 106]. Arkhipov and Agmon [105] showed that $\tau_{D}/\tau_{s}=(3k_{B}T/\mu_{d}^{2}\rho_{c})(\varepsilon_{s}-\varepsilon_{\infty })(2\varepsilon_{s}+\varepsilon_{\infty })/\varepsilon_{s}$, where ρ*_c_* is the density of molecules, and *μ_d_* is the dipole moment. In their review, Arkhipov and Agmon also discuss the relationship between macroscopic and microscopic relaxation times from various perspectives [105]. This theory predicts that the macroscopic and microscopic relaxation times are related by τ*_D_*/τ*_s_* ≈ (2ε*_s_* + ε_∞_) / 3ε*_s_*. Debye showed that the microscopic relaxation time for molecules of radius *a* is related to the viscosity η and the friction constant ζ by τ*_s_* = 4π*a*^3^η/*k_B_T* = ζ/2*k_B_T*. The Arrenhius relaxation rate is modeled as τ = τ_0_ exp (*U*/*k_B_T*). The Vogel-Fulcher relaxation time is used to model relaxation near polymer glass transition temperatures as τ = τ_0_ exp (*U*/*k_B_*(*T – T*_0_)). The relaxation time can also be related to changes in the activation entropy Δ*S*, Helmholtz energy of activation Δ*H*, and free energy Δ*F* as τ = (*ħ*/*k_B_T*) exp (Δ*F*/*RT*), and the entropy of activation is related by Δ*S* = (Δ*H –* Δ*F*)/*T*. Therefore, we have τ = (*h*/*k_B_T*) exp (Δ*H*/*RT* – Δ*S*/*R*). So, by fitting the relaxation times obtained by dielectric measurements as a function of temperature we can extract changes in the entropy Δ*S* and the Helmholtz free energy Δ*H* for an activation process.

The typical relaxation time *T*_1_ in NMR experiments is longer than in EPR [107]. In EPR experiments, relaxation times are generally less than milliseconds. In dielectrics, the relaxation times of liquids can be picoseconds, as indicated in Table 2, but in some glasses they can be seconds and longer. The characteristic relaxation times have been found to change with the frequency of the applied field [88]. This is due to the restoring and frictional forces acting differently under different field conditions. In the past researchers have realized this and resorted to using phenomenological DRT models as in Eq. (69).

### 11.2 Relaxation Time Based Model in Fields of Varying Frequency

A very general approach to modeling the susceptibility can be obtained by the Laplace transform of the time-invariant approximation to Eq. (38). This yields a permittivity in terms of complex relaxation times τ(ω) = τ′(ω) − *i*τ″(ω) [46]:

(115) $$\begin{array}{l} \varepsilon_{r}^{\prime }(\omega )=\varepsilon_{r\infty }+\\ \;(\varepsilon_{rs}-\varepsilon_{r\infty })\frac{1-\omega t^{\prime \prime }(\omega )}{{\left(\omega \tau^{\prime }(\omega )\right)}^{2}+{\left(1-\omega \tau^{\prime \prime }(\omega )\right)}^{2}}, \end{array}$$

(116) $$\begin{array}{l} \varepsilon_{r}^{\prime \prime }(\omega )=(\varepsilon_{rs}-\varepsilon_{r\infty })\\ \;\times \frac{\omega \tau^{\prime }(\omega )}{{\left(\omega \tau^{\prime }(\omega )\right)}^{2}+{\left(1-\omega \tau^{\prime \prime }(\omega )\right)}^{2}}+\frac{\sigma_{s}}{\varepsilon_{0}\omega }. \end{array}$$

The assumption of this model is that at RF frequencies the relaxation has a dependence on the frequency of the driving field. This frequency dependence originates from the applied field acting on the molecules in the material that keeps the molecules in a nonequilibrium electromagnetic state. Equations (115) and (116) have the same form as the Laplace transform of a linear harmonic oscillator equation of motion. However, this model contains additional information through the frequency dependence of the relaxation times. For a real, frequency-independent relaxation time (τ′ constant and τ″ = 0), Eq. (38) is the Debye equation. In the special case where τ′ is constant, the ensemble response function is of the form exp (−t/τ′) and we have classical Debye relaxation. This can be traced to the fact that the Debye model assumes there is no inertia, and therefore, a purely damped motion of dipoles. Performing the inverse Laplace transform of the time-invariant approximation to Eq. (38) we obtain another form for the polarization equation,

(117) $$\int_{0}^{t}\bar{\tau }(t-\theta )\frac{dP(\theta )}{d\theta }d\theta +P(t)=\chi_{s}E(t).$$

Equation (117) highlights the physics of the interaction with materials and is useful in determining the underlying differential equation related to phenomenological models. For this equation the Debye model is obtained if $\bar{\tau }(t)=\tau_{0}\delta (t)$. Relaxation phenomenological models such as Cole-Davidson can be related τ(ω). Therefore the underlying differential equations can be cast into the form of Eq. (117). Because they are complex pairs, it is not possible to extract the time-domain functions of τ′(ω) and τ″(ω) independently.

It is important to study the origin of the frequency-domain components. Whereas τ′(ω) models the out-of-phase behavior and loss, τ″(ω) models the effects of the local field on the restoring forces. If τ″(ω) is positive it is related to inertial effects. If τ″(ω) is negative, it is related to the local field interaction that tends to decrease the polarization through depolarization. The relaxation times are

(118) $$\tau^{\prime }(\omega )\omega =(\varepsilon_{r}^{\prime \prime }(\omega )-\frac{\sigma_{s}}{\varepsilon_{0}\omega })\frac{\left(\varepsilon_{s}-\varepsilon_{r\infty }\right)}{{\left(\varepsilon_{r}^{\prime }(\omega )-\varepsilon_{r\infty }\right)}^{2}+{\left(\varepsilon_{r}^{\prime \prime }(\omega )-\frac{\sigma_{s}}{\varepsilon_{0}\omega }\right)}^{2},}$$

(119) $$\tau^{\prime \prime }(\omega )\omega =-\frac{(\varepsilon_{r}^{\prime }(\omega )-\varepsilon_{r\infty })(\varepsilon_{s}-\varepsilon_{r}^{\prime }(\omega ))-{\left(\varepsilon_{r}^{\prime \prime }(\omega )-\frac{\sigma_{s}}{\varepsilon_{0}\omega }\right)}^{2}}{{\left(\varepsilon_{r}^{\prime }(\omega )-\varepsilon_{r\infty }\right)}^{2}+{\left(\varepsilon_{r}^{\prime \prime }(\omega )-\frac{\sigma_{s}}{\varepsilon_{0}\omega }\right)}^{2}}.$$

**Fig. 10:**
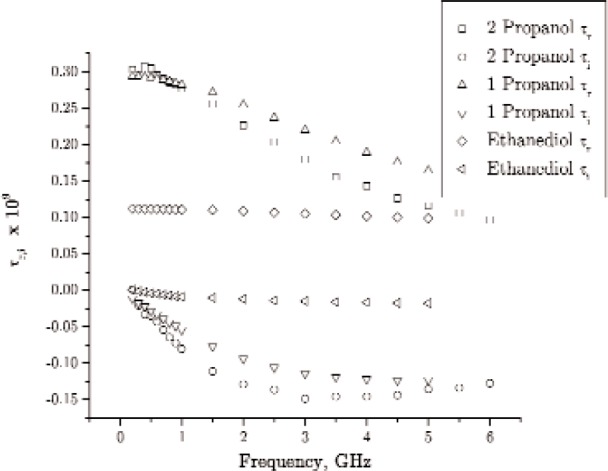
The real (τ*_r_*) and imaginary component (τ*_i_*) parts of the relaxation times for various alcohols.

In Fig. 10 we plot the relaxation times extracted from dielectric measurements as well as measurements given in Reference [108]. We see that the measured τ″(ω) values are all negative. We see that for ethanediol, τ″ is very small and τ′ is nearly frequency-independent. Therefore ethanediol, is well modeled by the Debye equation. The physical significance of τ′(ω) relates to the effective time for the material to respond to an applied electric field. τ″(ω) > 0 at resonance corresponds to an effective ensemble period of oscillation and τ″(ω) < 0 corresponds to a characteristic time scale for charge depolarization and screening effects. An interpretation is that in relaxation the effects of the local field on the short-range restoring forces and screening may have a frequency dependence. This frequency dependence can manifest itself as the commonly observed frequency shift in the loss peak relative to the Debye model. We also see that τ″ < 0 can be interpreted as the effects of the local field on the short-range electric restoring forces, which tend to reduce the permittivity and modify the position of the maximum in the loss curve relative to the Debye maximum condition (ωτ′ = 1). The behavior for τ″(ω) < 0 is analogous to what is seen in longitudinal optical-phonon behavior that yields a local field that tends to reduce polarization. Over frequencies where mass-related inertial interactions are important, τ″(ω) > 0. This occurs in polaritonic resonances at terahertz to infrared frequencies and in negative-index materials. In this case the local field tends to enhance the polarization through the effects of inertia that counteract restoring forces [5]. When τ″ω = 1, the real part of the susceptibility goes to zero, indicating the system is going through resonance. In general, just as in the Debye and other phenomenological models, the relaxation times can depend on temperature (*A* exp (*U*_0_/*k_B_T*)).

### 11.3 Surface Waves

Electromagnetic surface waves occur in many applications. Surface waves can be supported at the interface between dielectrics and conductors. These waves travel on the interface, but decay approximately exponentially away from the surface. There are many types of surface waves, including ground waves and surface plasmons polaritons (SPP) that travel at the interface between a dielectric and conductor, surface plasmons on metals, and Sommerfeld and Goubau waves that travel on coated or uncoated wires. SPP’s require the real part of the permittivity of the metal to be negative [109]. A Goubau line guides a surface wave and consists of a single conductor coated with dielectric material [110]. A Sommerfeld surface wave propagates as a TM mode around a finitely conductive single bare conductor. Plasmonic-like surface waves can form from incident microwave electromagnetic energy on subwavelength holes in metal plates. We will examine plasmonic surface waves in Sec. 14.2.

### 11.4 Electromagnetic Radiation

Classical electrodynamics predicts that accelerated charged particles generate EM waves. This occurs in antennas where charged particles oscillate to produce radiation. Linearly or elliptically polarized radiation waves are determined by the type of acceleration the source charged particles undergo. If the charge particle undergoes oscillation from a nonlinear restoring force, the emitted radiation may not be monochromatic.

### 11.5 Thermal Noise and Blackbody Fields

Due to the continual Brownian motion of microscopic charges, thermal Johnson noise fields are produced over a broad distribution of frequencies [111, 112]. There are also many other sources of noise such as phase noise and shot noise. Thermal movement and blackbody radiation are a source of electrical noise and was described theoretically by Nyquist [112]. This theory was expanded by Callen [113]. A blackbody has an emissivity of near unity and is an excellent absorber and emitter of radiation. The spectral distribution of blackbody radiation follows the Planck distribution for the energy density *u* (*T*, *f*) = (8π*h f*^3^/c^3^) / (exp (*h f*/*k_B_T* – 1)). Examples of blackbody radiation include radiation from intergalactic space, as well as black cavities with an aperture. Typical blackbody materials have some free electrons and a distribution of molecular resonant frequencies and, as a result, are useful in converting optical energy into heat energy. They are also good radiators of infrared thermal energy. Most materials only partially reflect any incident energy. Therefore, they do not radiate as much power as a blackbody at the same temperature. The ratio of the energy radiated by a material relative to that of an ideal black body is the emissivity. In a frequency band Δ*f*, the emissivity is defined as *e* = *P*/(*k_B_T*Δ*f*). The emissivity satisfies 0 ≤ *e* ≤ 1. The brightness temperature is *T_B_* = *eT*, where *T* is the physical temperature. Nyquist/Johnson noise in the RF band has only a weak frequency dependence. It is modeled for voltage fluctuations in a transmission line terminated by resistors *R* over a frequency band Δ*f* by <ν^2^>/*R* = 4*k_B_T*Δ*f* [112].

Radiometers in the RF band are usually receiving antennas that collect noise power from the direction they are pointed and infer the brightness temperature. The goal of radiometry is to infer information about the remote source of noise from the brightness temperature [111].

Quantum-field theory models the vacuum as filled by quantum fluctuations that contain a spectrum of frequencies having energy (1/2) *ħ*ω. In this model, fluctuations give rise to virtual photons and spontaneous emission of short-lived particles. Virtual photons and short-lived particles are allowed by the uncertainty principle between energy and time: Δ*E*Δ*t* ≈ *ħ*.

Vacuum fluctuations can produce attractive forces between nanometer-spaced parallel electrodes. This Casimir effect is commonly explained classically by the cutoff of EM modes between the plates so that the external radiation pressure exceeds the pressure between the plates [114]. A more complete and satisfactory description can be derived with quantum mechanics. The force is extremely short range. It has also been shown that the force can be made repulsive by changing one of the plates from a metal to a dielectric such as silica [115]. In addition, there has been speculative research where NIM materials are used for the microscopic plates to produce levitation of nanoparticles [116]. Casimir effects may play a role in future modeling of microelectronics because the electrode separations are close to where these effects become important.

## 12. Magnetic Response

### 12.1 Overview of Magnetism

In this section, we will very briefly overview the basic elements of magnetic phenomena needed in our applications to RF interactions. Magnetism has a quantum-mechanical origin intimately related to the spin and angular momentum and currents of electrons, nuclei, and other particles. Stern and Gerlach [4] proved the existence of discrete magnetic moments by observing the quantized deflection of silver atoms passing through a spatially varying magnetic field. Electrons orbiting a nucleus form a magnetic moment as well as the intrinsic spin of the electron. Magnetic moments are caused either by intrinsic quantum-mechanical spin or by currents flowing in closed loops **m** ∝ (current)(area).

Spins react to a magnetic field by precessing around the applied field with damping [117]. For spins of the nucleus, this precession forms the study of nuclear magnetic resonance (NMR); for paramagnetic materials it is called electron-spin or ESR or electron-paramagnetic resonance or EPR; and for ferromagnetic materials it is called ferromagnetic resonance or FMR. The dynamics in spin systems are tied phenomena such as spin precession, relaxation, eddy currents, spin waves, and voltages induced by domain-wall movements [7–9, 118].

**Fig. 11:**
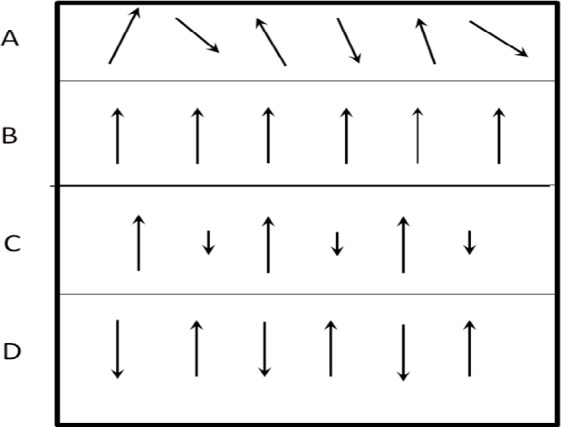
Simplistic summary of spin orientations for A) paramagnetic B) ferromagnetic C) ferrimagnetic D) antiferromagnetic materials.

Paramagnetism originates from spin alignment in an applied magnetic field and relates to the competition between thermal versus magnetic energy (**m**·**B**/*k_B_T*) (see A in Fig. 11). Paramagnets do not retain significant magnetization in the absence of an applied magnetic field, since thermal motion tend to randomize the spin orientations.

The origin of diamagnetism in materials is the orbital angular momentum of the electrons in applied fields. Diamagnetic materials usually do not have a strong magnetic response, although there are exceptions. In ferromagnetic materials, exchange coupling allows regions of aligned spins to be formed [119]. Ferromagnetic and ferrimagnetic materials may have spin resonances in microwave to millimeter wave frequencies [120]. Ferrimagnetic materials consist of two overlapping lattices whose spins are oppositely directed, but with a larger magnetic moment in one lattice than the other. Antiferromagnetism is a property of many transition elements and some metals. In these materials the atoms form an array with alternating spin moments, so the average spin and magnetic moment are zero. Antiferromagnetic materials are composed of two interpenetrating lattices. Each lattice has all spins more or less aligned, but the lattices, as a whole, are inverse. Resonances in antiferromagnetic materials may occur at millimeter wave frequencies and above. Antiferromagnetic materials are paramagnetic above the Neel temperature.

#### 12.1.1 Two-State Spin System

In order to contrast decoupled spin response with dielectric dipole response in Sec. 10.2, we will develop the well-known statistical approach of noninteracting paramagnetism. In a paramagnetic material, the net magnetic moment is the sum of individual moments in an applied field. If the spin moments are σ_±_ = ±*μ* and the probability density of the spin being up or down in an applied field is *p_i_*, then the net magnetic moment is [4]

(120) $$<m>=-\sum_{i}\sigma_{i}p_{i}(\sigma ),$$

where the probabilities of being in the low energy (−) or high (+) energy states are

(121) $$p-=\frac{e^{\vec{\mu }\cdot B/k_{B}T}}{e^{\vec{\mu }\cdot B/k_{B}T}+e^{-\vec{\mu }\cdot B/k_{B}T}},$$

(122) $$p+=\frac{e^{\vec{\mu }\cdot B/k_{B}T}}{e^{\vec{\mu }\cdot B/k_{B}T}+e^{-\vec{\mu }\cdot B/k_{B}T}},$$

Therefore, for *N* spins and when $\vec{\mu }\cdot B/k_{B}T\ll 1$

(123) $$<m>=N\mu \frac{e^{\vec{\mu }\cdot B/k_{B}T}-e^{-\vec{\mu }\cdot B/k_{B}T}}{e^{\vec{\mu }\cdot B/k_{B}T}+e^{-\vec{\mu }\cdot B/k_{B}T}}=N\mu \tanh\frac{\vec{\mu }\cdot B}{k_{B}T}\approx N\mu \frac{\vec{\mu }\cdot B}{k_{B}T}.$$

In the case of isotropy < *m* > = *Nμ*^2^*B*/3*k_B_T*. So we obtain the same form as in the case of noninteracting dielectrics in Eq. (107).

#### 12.1.2 Paramagnetic Response With Angular Momentum *J*

For atoms with angular momentum *J* with 2*J* + 1 discrete energy levels, the average magnetization can be expressed in terms of the Brillioun equation *B_J_* [4]

(124) $$<m>=NgJ\mu_{B}B_{J}(x),$$

where *x* = *gJμ_B_B* / *k_B_T* and *B_J_* (*x*) = (2*J* + 1) / 2*J* coth((2*J* + 1)*x*/2*J*) − (1/2*J*) coth(*x*/2*J*) and *g* is the *g*-factor given by the Landé equation.

### 12.2 Magneto-Dielectric Response: Magneto-Electric, Ferroelectric, Ferroic, and Chiral Response

Researchers have found that in magneto-electric, ferroic, and chiral materials the application of magnetic fields can produce a dielectric response and the application of an electric field can produce a magnetic response (see for example [121]). These cross coupling behaviors can be found to occur in specific material lattices, layered thin films, or by constructing composite materials. An origin of the intrinsic magneto-electric effect is from the strain-induced distortion of the spin lattice upon the application of an electric field. When a strong electric field is applied to a magneto-electric material such as chromium oxide, the lattice is slightly distorted, which changes the magnetic moment and therefore the magnetic response. Extrinsic effects can be produced by layering appropriate magnetic, ferroelectric, and dielectric materials in such a way that an applied electric field modifies the magnetic response and a magnetic field modifies the electric response. Chiral materials can be constructed by embedding conducting spirals into a dielectric matrix. In artificial magneto-electric materials the calculated permittivity and permeability may be effective rather than intrinsic properties. The constitutive relations for the induction and displacement fields are not always simple and can contain cross coupling between fields. For example, $\tilde{D}(\omega )={\overset{↔}{\alpha }}_{1}\cdot \tilde{E}(\omega )+{\overset{↔}{\alpha }}_{2}\cdot \tilde{B}(\omega )$, where ${\overset{↔}{\alpha }}_{i}$ are constitutive parameters.

## 13. Electromagnetic-Driven Material Resonances in Materials at RF Frequencies

At the relatively longer wavelengths of RF frequencies, (1 × 10^4^ m to 1 mm), only a few classes of intrinsic resonances can be observed. Bulk geometric resonances, standing waves, and higher-mode resonances can occur at any frequency when an inclusion has a dimension that is approximately equal to an integral multiple of one-half wavelength in the material. These geometrical resonances are sometimes misinterpreted as intrinsic material resonances. Most of the intrinsic resonant behavior in the microwave through millimeter frequency bands are due to cooperative ferromagnetic and ferrite spin-related resonances, antiferromagnetic resonances, microwave atomic transitions, plasmons and plasmon-like resonances, and polaritons at metal-dielectric interfaces. Atoms such as cesium have transition resonances in the microwave band. Large molecules can also be made to resonate under the application of high RF frequencies and THz frequencies. NIM commonly use non-intrinsic split-ring structure resonances together with plasma resonances to achieve unique electromagnetic response. At optical frequencies, individual molecules or nanoparticles can sometimes be resonated directly or through the use of plasmons.

Water has a strong relaxation in the gigahertz frequency range and water vapor has an absorption peak in the gigahertz range, liquid water has no dielectric resonances in the microwave range. The resonances of the water molecule occur at infrared frequencies at a wavelength around 9 μm. In magnetic materials, ferromagnetic spin resonances occur in the megahertz to gigahertz to yielding MMW bands. Antiferromagnetic resonances can occur at millimeter frequencies. Gases such as oxygen with a permanent magnetic moment can absorb millimeter waves [122]. In the frequency region from 22 to 180 GHz, water-vapor absorption is caused by the weak electric dipole rotational transition at 22 GHz, and a stronger transition occurs at around 183 GHz [123].

If high-frequency fields are applied to ferrite materials, there are relaxations in the megahertz frequencies, and in the megahertz to MMW frequencies there are spin resonances [119, 121, 124, 125].

## 14. Artificial Materials: Plasmons, Super-Lensing, NIM, and Cloaking Response

The term metamaterial refers to artificial structures that can achieve behaviors not observed in nature. NIMs are a class of metamaterials where there are simultaneous resonances in the permittivity and permeability. Many artificial materials are formed from arrays of periodic unit cells formed from dielectric, magnetic, and metal components, and when subjected to applied fields, achieve interesting EM response. Examples of periodic structures are NIM that utilize simultaneous electric and magnetic resonances [126]. Metafilms, band filters, cloaking devices, and photonic structures all use artificial materials. Artificial materials are also used to obtain enhanced lensing and anomalous refraction and other behaviors [65, 126–131]. A very good overview is given in [128]. In the literature NIM materials are commonly assumed to possess an intrinsic negative permittivity and permeability. However, the resonator dimensions and relevant length scales used to achieve this behavior may not be very much smaller than a wavelength of the applied field [132]. Therefore, the continuous media requirement for defining the permittivity and permeability becomes blurred. The mapping of continuous media properties onto metamaterial behavior can at times cause paradoxes and inconsistencies [69, 133–137]. However, the measured EM scattering response in NIM is achieved, whether or not an effective permittivity and permeability can be consistently defined. Because of the inhomogeneity in the media, the permittivity and permeability in some of these applications are effective parameters and spatially dispersive and not the intrinsic properties that Veselago assumed for a material [26, 138]. In some metamaterials and metafilms where the ratio of the particle size to the wavelength is not small, boundary transition layers are typically included in the model so that the terminology of effective permittivity and permeability can be used. In Sec. 4.6, we described the criterion of defining a polarization by a Taylor series expansion of the charge density. The problem of whether these composite materials can be described in terms of a negative index is complicated by the issues described above. The measured permittivity tensor is an intrinsic property and should not depend on the field application or the sample boundaries, if the electrodynamic problem is modeled correctly.

Pendry [127] introduced the idea of constructing a lens from metamaterials that could achieve enhanced imaging that is not constrained by the diffraction limit. It should be noted that microwave near-field probes also have the capability of subwavelength imaging by using the near field around a probe tip (see Sec. 16).

### 14.1 Veselago’s Argument for NIM Materials With Both ε′*_r_*(*eff*) < 0 and μ′*_r_*(*eff*) < 0

In this section we overview the theory behind NIM [26]. The real parts of the permittivity and permeability can be negative over a band of frequencies during resonances. Of course, to maintain energy conservation in any passive material, the loss-factor part of the permittivity and permeability must always be positive. This behavior has only been recently exploited to achieve complex field behavior [26, 62, 67, 88, 127, 139].

Polarization resonance is usually modeled by a damped harmonic-oscillator equation. The simple harmonic-oscillator equation for the polarization $\tilde{P}(\omega )$ for single-pole relaxation can be written as Eq. (58). For a time-harmonic-field approximation, the effective dielectric susceptibility has the form

(125) $$\chi_{d}(\omega )=\frac{\chi_{0}}{-\omega^{2}/\omega_{0}^{2}+i\omega \tau +1}.$$

The real part of the susceptibility can be negative around the resonance frequency (see Fig. 7). A similar equation can apply for a resonance in a split-ring or other resonator to obtain a negative real part of the permeability.

In most electromagnetic material applications the plane-wave propagation vector and group velocities are in the same direction. Backward waves are formed when the group velocity and phase velocity are in opposite directions. This can be produced when the real parts of the permittivity and permeability are simultaneously negative. When this occurs, the refractive index is negative, $n=\sqrt{-\left|\varepsilon_{r}^{\prime }\right|}\sqrt{-\left|\mu_{r}^{\prime }\right|}=i\sqrt{\left|\varepsilon_{r}^{\prime }\right|}i\sqrt{\left|\mu_{r}^{\prime }\right|}=-\sqrt{\left|\varepsilon_{r}^{\prime }\right|\left|\mu_{r}^{\prime }\right|}$. Because of this result, researchers have argued that this accounts for the anomalous refraction of waves through NIMs, reverse Cherenkov radiation, and reverse Doppler effect, etc.

Snell’s law for the reflection of an interface between a normal dielectric and an NIM satisfies θ*_inc_* = θ*_reflection_*, but the refracted angle in NIM is θ*_trans_* = *sgn*(*n_NIM_*) $\sin^{-1}\left(\frac{n_{norm}}{n_{NIM}}\sin\theta_{inc}\right)$ [140]. In addition, the TEM wave impedance of plane waves for NIM is $Z=\sqrt{\frac{\mu_{0}}{\varepsilon_{0}}}\sqrt{\frac{-\left|\mu_{r}^{\prime }\right|-\left|i\mu_{r}^{\prime \prime }\right|}{-\left|\varepsilon_{r}^{\prime }\right|-i\left|\varepsilon_{r}^{\prime \prime }\right|}}$. If only the real part of the the permittivity or the permeability are negative, then damped field behavior is attained.

These periodic artificial materials do produce interesting and potentially useful scattering behavior; however since they often involve resonances in structures that contain metals, they are lossy [62]. There has been debate in the literature over how to interpret the observed NIM behavior, and some researchers believe the results can be explained in terms of surface waves rather than invoking NIM concepts [137].

The approach used to realize a negative effective magnetic permeability is different from that for obtaining a negative effective ε*_r_*′. Generally, split-ring resonators are used to obtain negative μ*_r_*′, but recently there has been research into the use of TM and TE resonant modes in dielectric cubes [69] or ferrite spheres to achieve negative properties [62, 141]. Dielectric, metallic, ferrite, or layered dielectric-metallic inclusions such as spheres can be used to achieve geometric or coupled resonances and therefore simultaneous negative effective ε′ and μ′ [62]. A commonly used approach to obtain a negative permittivity is to drive the charges in a wire or free charge in a semiconductor or plasma near resonance. Dielectric resonance response occurs in semiconductors in the terahertz to infrared range and in superconductors in the millimeter range. The real part of the permittivity for a plasma, according to the high-frequency Drude model, can be negative $\left(\varepsilon =\varepsilon_{0}\left(1-\omega_{p}^{2}/\omega^{2}\right)\right)$.

There are a number of metrology issues related to NIM. These include the problem of whether the field behavior should be modeled as the result of negative intrinsic permittivity and permeability and negative index or instead be treated as a scattering problem. This problem is related to the wave length of the applied fields versus the parameters of the embedded resonators. Although the scatterers are generally smaller than a wavelength of the applied field, they are not always significantly smaller. When the lattice spacing *a* between particles satisfies [142] $0\leq \left|\sqrt{\varepsilon_{r}^{\prime }\mu_{r}^{\prime }}\right|\omega a/c\leq 1$, then effective properties can be defined [62]. Even within these bounds the properties are not intrinsic permittivity and permeability as defined previously and are spatially dispersive. A second issue is the determination of the NIM specimen length and boundaries to be used to model the array of macroscopic scatterers (see [69] and references therein for an analysis of this problem). Another area of debate is where in the resonance region is a permittivity and permeability well defined.

### 14.2 Plasmonic Behavior

**Fig. 13:**
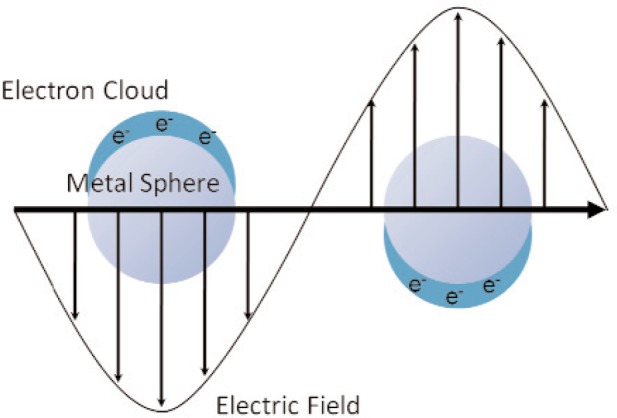
Plasmon resonance.

At the interface between a dielectric and metal an EM wave can excite a quasiparticle called a surface polariton (see Fig. 13). Plasmons are charge-density waves of electron gases in plasmas, metals, or semiconductors. Surface polariton plasmons travel on the interface between a dielectric and a conductor, analogous to the propagation of the Sommerfeld surface wave on a conductor/dielectric interface. Plasma polaritons decay exponentially away from the surface. The effective wavelengths of plasmons are much shorter than that of the incident EM field and therefore plasmons can propagate through structures where the incident radiation could not propagate through. This effect has been used in photonics and in microwave circuits through the use of metamaterials. For example, thin metal films can be embedded in dielectrics to form dielectric waveguides. Plasmonics is commonly used for imaging where the fields are used to obtain a sub-wavelength increase in resolution of 10 to 100 times. Colors in stained glass and metals are related to the plasma resonance frequency, due to the preferred reflection and absorption of specific wavelengths. High-temperature superconductors also have plasmonic behavior and a negative ε′*_r_* due to the complex conductivity [91]. If small metallic particles are subjected to EM radiation of the proper wavelength, they can confine EM energy and resonate as surface plasma resonators. Plasmonic resonances have also been used to clean carbon nanotubes and enhance other chemical reactions by thermal or nonthermal activation. Plasmons have been excited in metamaterials by use of a negative permeability rather than negative permittivity [143].

#### Bulk Plasmons

Maxwell’s equations with no source-current densities can be used to obtain

(126) $$-\mu_{0}\frac{\partial^{2}D}{\partial t^{2}}=\nabla \times \nabla \times E.$$

If **E** ∝ *e*^iω^*^t^*^−ikz^, the dispersion relation is

(127) $$k(k\cdot E)-k^{2}E=-\varepsilon_{r}(k\cdot \omega )\frac{\omega^{2}}{c^{2}}E.$$

For transverse plane waves **k**·**E** = 0, and therefore *k*^2^ = ε*_r_* (**k**, ω)ω^2^/*c*^2^. For longitudinal waves ε*_r_*(**k**, ω) = 0 [144] (this condition ε(ω) = 0) also implies the Lyddane-Sachs-Teller relation [102] for the ratio of the longitudinal to transverse phonon frequencies that satisfies $\frac{\omega_{L}^{2}}{\omega_{T}^{2}}=\frac{\varepsilon_{s}}{\varepsilon_{\infty }}$*L*.

From Eq. (62), in the time domain for the case of no loss, and **P**(*t*) = − *Ne****x***(*t*), where *N* is the density of electrons, we obtain the equation for a harmonic oscillator for bulk longitudinal plasmon oscillations, $d^{2}x/dt^{2}=-\omega_{p}^{2}x$. The permittivity of a plasmon can be modeled as

(128) $$\varepsilon (\omega )=\varepsilon_{0}\left(1-\frac{\omega_{p}^{2}}{\omega^{2}}\right).$$

Below the plasmon frequency $\omega_{p}=\sqrt{Ne^{2}/\varepsilon_{0}m}$, the plasma is attenuative and follows the skin-depth formulas of a metal.

Above the plasma frequency, the real part of the permittivity becomes negative.

#### Surface Plasmons

Surface plasmon polaritons [144] can travel at the interface of a metal and dielectric to produce surface wave guiding. Plasmonic surface waves have fields that decay rapidly from the surface interface. For example, for a 1 μm excitation wavelength, the waves can travel over 1 cm, leading to the possibility of applications in microelectronics. Surface plasmonic EM waves can be squeezed into regions much smaller than allowed by the diffraction limit. Obtaining the negative effective ε′*_rp_* for plasmons in the megahertz through MMW range would require the use of NIM. Some applications of plasmonic behavior can also be tuned by a dc external magnetic field, and the applied magnetic field produces a plasmon with a tensorial permittivity.

For surface plasmons, the effective wavelengths of the plasmons can be much less than that of the exciting EM fields due to the difference in sign of the permittivities in a metal and dielectric. For example, for a plasmon at an interface between a metal and a dielectric substrate, if the permittivity of the plasmon is ε′*_rp_* and that of the substrate is ε′*_rd_*, then the dispersion relation is $k=2\pi /\lambda =(\omega /c)\sqrt{\varepsilon_{rd}\varepsilon_{rp}/(\varepsilon_{rd}+\varepsilon_{rp})}$ [144]. When $ℛ(\varepsilon_{rp}^{\prime })<0$ and $\left|ℛ(\varepsilon_{rp}^{\prime })\right|$ is slightly larger than ε′*_rd_*, then we see that the wavelength becomes very short in comparison to that of the applied field. This is also attained by application of laser light to nanoparticles to obtain a resonant state. However, this can also happen in coupled microwave resonant structures.

### 14.3 Transmission Through Subwavelength Apertures

Under certain conditions, electromagnetic radiation has been observed to pass through subwavelength apertures [145–147]. In extraordinary optical (EOT) or millimeter wave (EMT) transmission, free-space EM waves impinging on a metal plate with small holes transmits more energy than would be expected by a traditional analysis [148]. At optical frequencies, this transmission is mediated by surface plasmons. At MW and MMW frequencies, plasmons are not formed on homogeneous conducting metal plates. However, plasmon-like behavior can be formed by an appropriate selection of holes, metal plate thickness, or corrugations to produce a behavior that simulates surface plasmons. These plasmons-like features that are sometimes referred by the jargon “spoof plasmons”, can be the origin of extraordinary transmission through the holes in metal plates at MW to MMW frequencies.

### 14.4 Behaviors in Structures Where ε′*_r_*_(_*_eff_*_)_ → 0

There are applications where a material is constructed in such a way so that the real part of the “effective” permittivity is close to 0 (ENZ) (see Fig. 7 as ε′*_r_* → 0). This is closely related to plasmon-like behavior. In this case, the EM behavior simulates static behavior in that ∇ × **H** = 0 and ∇ × **E** = *i*ω*μ***H**, which implies ∇^2^**E** = 0. In this case, the phase velocity approaches infinity and the guided wavelength becomes infinite, which is analogous to cutoff in a waveguide (λ*_c_*) [47]. This type of behavior can be achieved for a waveguide near cutoff. The equation for the guided wavelength in a waveguide is

(129) $$\lambda_{g}=\frac{2\pi }{\sqrt{\varepsilon_{r}\mu_{r}\frac{\omega^{2}}{c^{2}}-{\left(\frac{2\pi }{\lambda_{c}}\right)}^{2}}}=\frac{\lambda }{\sqrt{1-{\left(\frac{\lambda }{\lambda_{c}}\right)}^{2}}},$$

where λ*_c_* is the cutoff wavelength of the guide. Due to the long effective wavelength near cutoff, the phase of the wavefront changes minimally. Because the effective permittivity goes through zero near resonance, we can think of ENZ as a resonance condition similar to the propagation cutoff in a waveguide when there is resonance in the transverse plane. This type of behavior is achieved, for example, if we have a low-loss dielectric of length *L* that completely fills the cross section of a waveguide (see Fig. 24). Near the cutoff frequency the material could be thought of as having an effective permittivity ε′*_r_*_(_*_eff_*_)_ ≈ 0. This same behavior is reminiscent of a cavity, because as the transmission attains a maximum, reflection is a minimum, and the reactance goes to 0 near resonance. The ε′_(_*_eff_*_)_ in this model violates the condition for an intrinsic permittivity since the applied field wavelength $\left(\lambda =1/f\surd \bar{\varepsilon \mu }\right)$, must be much larger than the feature size. It has been argued that in ENZ, unlike in a normal wire, the displacement current dominates over the charge current in transporting the EM waves [146]. There could be analogous effective permeability going to zero *μ*′*_r_*_(_*_eff_*_)_ → 0 (MNZ) behavior.

### 14.5 Modeling Electrical Properties to Produce Cloaking Behavior

Recently, there have been many research papers that examine the possibility of using the electrical properties of artificial materials to control the scattering from an object in such a way as to make the object appear invisible to the applied EM field [129, 130, 149]. This is distinct from radar-absorbing materials, where the applied field is absorbed by ferrites or layered, lossy materials. Research in this area uses the method of transformation optics [149, 150] to determine the material properties that produce the desired field behavior. In order to exhibit a typical cloaking property, Shivola [151] derived simple equations for a dielectric-layered sphere that are assigned permittivities to produce a nearly zero effective polarizability. Recently, complex arrangements of non-resonant metamaterials have been designed by inverse optical modeling to fabricate broadband electromagnetic cloaks [129, 152].

## 15. Macroscopic to Mesoscopic Heating and Electromagnetic-Assisted Reactions

### 15.1 Overview of EM Heating

#### 15.1.1 Dielectric and Magnetic Heating

In EM wave interactions with materials, some of the applied energy is converted into heat. The heating that takes place with the application of high-frequency fields is due to photon-phonon processes modeled by the friction caused by particle collisions and resistance to dipole rotation. Over the RF spectrum, heating may be volumetric at low frequencies and confined to surfaces at high frequencies. Volumetric heating is due to the field that penetrates into the material producing dissipation through the movement of free ions and the rotation of dipolar molecules. Nanocomposites can be heated volumetrically by RF EM fields, lasers, and terahertz applicators. Since the skin depth is long at low frequencies, the heating of nanoparticles is not efficient. In the microwave band the heating of very small particles in a host material is limited by the loss and density of particles in the material, the power level of the source, and the diffusion of heat to the surroundings. Plasmon resonances in the infrared to visible frequencies can be used to locally heat particles [153]. At high frequencies, heat may be absorbed locally in particles in slow modes where there may be a time lag for heat to dissipate into the phonon bath when the fields are removed.

The history of practical RF heating started in the era when radar was being developed. There are stories of where engineers sometimes heated their coffee by placing it near antennas. Also there are reports a researcher working on a magnetron that noticed that the candy bar in his pocket had melted when he was near the high-frequency source.

In a microwave oven, water and bound water are heated by the movement of free charge and non-resonant rotation [154]. Because the water molecules at these frequencies cannot react in concert with the field, energy is transferred from the field energy into kinetic energy of the molecules in the material. In dielectric materials at low frequencies, as frequencies increase into the HF band, the rotations of the molecules tend to lag the electric field, and this causes the electric field to have a component in phase with the current. This is especially true in liquids with hydrogen bonding, where the rotational motion of the bonding is retarded by the interconnections to other molecules. This causes energy in the electric and magnetic fields to be converted into thermal energy [155]. Some polymer molecules that have low friction, such as glycerol in solution, tend to rotate without significant molecule-molecule interactions and therefore produce little thermal energy.

The power dissipated in a bulk lossy material in a time-harmonic field is

(130) $$P(\omega ,T)=\frac{1}{2}\omega \int \left(\varepsilon^{\prime \prime }(\omega ,Τ){\left|\tilde{E}(\omega )\right|}^{2}+\mu^{\prime \prime }(\omega ,T){\left|\tilde{H}(\omega )\right|}^{2}\right)dV.$$

The total entropy produced per unit time at a temperature *T* is *P*(ω, *T*)/*T*. Equation (130) is modified for very frequency-dispersive materials [116]. Dielectric losses in ohmic conduction and Joule heating originate in the frictional energy created by charges and dipoles that are doing work against nonconservative restoring forces. Magnetic losses include eddy currents, hysteresis losses, and spin-lattice relaxation. Some of the allocated heating frequencies are given in Table 3.

Heating originates from dielectric and magnetic loss and the strength of the fields. For magnetic materials the losses relate to *μ*″(ω) and σ*_dc_*. In high-frequency fields, magnetic materials will be heated by both dielectric and magnetic mechanisms [104, 156]. If applicators are designed to subject the material to only magnetic or electric fields, then the heating will be related only to magnetic or dielectric effects, respectively.

When studying dielectric heating we need to also model the heat transport during the heating process. This is accomplished by use of the power dissipated as a source in the heat equation [157]. The transport of heat through a material is modeled by the thermal diffusivity α*_h_* = κ/ρ*_d_c_p_*, where ρ*_d_* is the density and κ is the thermal conductivity. In order to model localized heating, it is necessary to solve the Fourier heat equation and Maxwell’s equations with appropriate boundary conditions. The macroscopic heat transfer equation is

(131) $$\rho_{d}c_{p}\frac{\partial T}{\partial t}=\nabla \cdot \overset{↔}{\kappa }\cdot \nabla T+\frac{1}{2}\omega \varepsilon^{\prime \prime }{\left|E\right|}^{2},$$

where $\overset{↔}{\kappa }$ is the thermal conductivity dyadic. The mass density is ρ*_d_* and the specific heat is *c_p_*. For nanosystems, the heat transfer is more complicated and may require modeling phonon interactions. Also, the above heat transfer expression is only approximate for nanoscale materials. The temperature rise obtained by application of EM energy to a material can be estimated by use of the power dissipation relation in Eq. (130). When the temperature is changed by Δ*T*, the thermal energy-density increase is *Q_h_* = ρ*_d_c_p_*Δ*T*. The power dissipated per unit volume by an electric field interacting with a lossy dielectric material is *P_d_* = (1/2)σ|**E**|^2^, where σ is the conductivity. Therefore, the temperature rise in a specimen with density ρ*_m_* through heating with a power *P_d_* for a time Δ*t* is

(132) $$\Delta T=\frac{\sigma {\left|E\right|}^{2}\Delta t}{2\rho_{m}c_{p}}.$$

The heating rate is determined by the field strength, frequency, and the loss factor. From the equations for the skin depth $\delta_{s}\approx (1/\omega )\sqrt{\mu^{\prime }\varepsilon^{\prime }/2}\sqrt{\sqrt{\left(1+\tan^{2}\delta \right)}-1}$, we see that fields at lower frequencies will penetrate more deeply $\left(\delta_{s}\to 2_{c}\sqrt{\varepsilon^{\prime }}/\omega \sqrt{\mu_{r}^{\prime }}\varepsilon_{r}^{\prime \prime }\right)$. In order to obtain the same dissipative power densities as those at higher frequencies, the electric field strength at a lower frequency would have to increase. For example, to obtain the same power densities at two different frequencies we must have $\left(\varepsilon_{1}^{\prime \prime }(\omega_{1})\omega_{1}/\varepsilon_{2}^{\prime \prime }(\omega_{2})\omega_{2}={\left|E_{2}\right|}^{2}/{\left|E_{1}\right|}^{2}\right)$.

The unique volumetric heating capability by EM fields over broader ranges of frequencies should stimulate further applications in areas such as recycling, enhanced oil recovery, and as an aid to reactions.

#### 15.1.2 Electromagnetic-Assisted Reactions

When RF waves are applied to assist a chemical reaction, or polymer curing, the observed rate enhancement is due primarily to the effects of microscopic and volumetric heating. Because chemical reaction rates proceed in an Arrenhius form τ ∝ exp (*E*/*k_B_T*), small temperature increases can produce large reductions in reaction times. The kinetics of chemical reaction rates is commonly modeled by the Eyring equation,

(133) $$k_{eyr}=\frac{k_{B}T}{h}e^{-\Delta G/RT},$$

where *h* is Planck’s constant, Δ*G* = Δ*H* − *T*Δ*S* is the Gibb’s free energy, *H* is Helmholtz’s free energy, Δ*S* denotes changes in entropy, and *R* is the gas constant. A plot of ln (*k_eyr_*/*T*) = − Δ*H*/*RT* + ΔS/*R* + ln (*k_B_*/*h*) versus 1/*T* can yield Δ*S*, Δ*H*, and possibly *k_B_*/*h*.

One would expect that heat transfer by conduction would have the same effect on reactions as microwave heating, but this is not always found to be true. Part of the reason for this is that thermal conduction requires strong temperature gradients, whereas volumetric heating does not require temperature gradients. Because it does not depend on thermal conduction, an entire volume can obtain nearly the same temperature simultaneously without appreciable temperature gradients. In addition, some researchers speculate on non-thermal microwave effects that are due to the electric field interacting with molecules in specific ways that modify the activation energy through changes in the entropy [158, 159]. Avenues that have been proposed for nonthermal reactions may be related to dielectric breakdown that causes plasma of photons to be emitted, causing photo-reactions. Another avenue is related to the intense local fields that can develop near corners or sharp bends in materials or molecules that cause dielectric breakdown.

Typical energies of microwave through x-ray photons are summarized in Table 4. Covalent bonds such as C-C and C-O bonds have activation energies of nearly 360 kJ/mol, C-C and O-H bonds are in the vicinity of 400 kJ/mol, and hydrogen bonds are around 4 to 42 kJ/mol. Microwaves are from 300 MHz to 30 GHz and have photon energies from 0.0001 to 0.11 kJ/mol. Therefore, microwave photon bond-breaking events are rare. Nonthermal microwave effects, therefore, are not likely due to the direct interaction of microwave photons with molecules and, if they occur at all, and must have secondary origins such as the generation of intense local fields that produce localized dielectric breakdown or possibly EM-induced changes in the entropy. Most of the effects seen in microwave heating are thermal effects due to the volumetric heating of high-frequency fields [160].

Microwave heating can result in superheating where the liquid can become heated above the typical boiling point. For example, in microwave heating, water can be heated above its boiling temperature. This is due to the fact that in traditional heating, bubbles form to produce boiling, whereas in microwave heating the water may become superheated before it boils.

### 15.2 Heat Transfer in Nanoscale Circuits

In microelectronic circuits, higher current densities can cause phonon heating of thin interconnects that can cause circuit failure. This heating is related to both the broad phonon thermal bath and possibly slow thermal modes where thermal energy can be localized to nanoscale regions [161, 162]. New transistors will have an increased surface-to-volume ratio and, therefore, the power densities could increase. This, combined with the reduced thermal conductance of the low conductivity materials and thermal contact resistance at material interfaces, could lead to heat transport limitations [162, 163].

### 15.3 Heating of Nanoparticles

When a large number of metallic, dielectric, or magnetic micrometer or nanometer particles in a host media are subjected to high-strength RF EM fields, energy is dissipated. This type of EM heating has been utilized in applications that use small metallic particles, carbon black, or palladium dispersed in a material to act as chemical-reaction initiators and for selective heating in enhanced drug delivery or tumor suppression [164, 165]. Understanding the total heat-transfer process in the EM heating of microscopic particles is important. A number of researchers have found that, due to the thermal conduction of heat from nanoparticles and the small volumes involved and the large skin depths of RF fields, the nanoparticles rapidly thermalize with the phonon bath and do not achieve temperatures that deviate drastically from the rest of the medium [166]. Only when there is an appropriate density of particles, is heating enhanced. There have recently been reports that thermal energy can accumulate in nanoscale to molecular regions in slow modes, and it can take seconds to thermalize with the surrounding heat bath [166–169]. In such situations, regions may be unevenly heated by field application. However, thermal conduction will tend to smooth the temperature profile within a characteristic relaxation time. Lasers can selectively heat micrometer-size particles and by use of plasmonics lasers can heat conducting nanoscale particles.

### 15.4 Macroscopic and Microscopic High-Frequency Thermal Run-Away

The dielectric loss and thermal conductivity of a material may possess a temperature dependence so that the loss increases as temperature increases [170]. This is due to material decomposition that produces ions as the temperature increases and results in more loss. Thermal run away can lead quickly to intense heating of materials and dielectric breakdown. The temperature dependence of thermal run away has been modeled with the dielectric loss factor as ε*_r_*″ = α_0_ + α_1_ (*T* − *T*_0_)+ α_2_(*T* − *T*_0_)^2^, where *T*_0_ is a reference temperature and α*_i_* are constants [171].

## 16. Overview of High-Frequency Nanoscale Measurement Methods

In the past few decades, a number of methods have been developed to manipulate single molecules and dipoles. Methods have been implemented to move, orient, and manipulate nanowires, viruses, and proteins that are several orders of magnitude smaller than cells. These methods allow the researcher to study the electrical and mechanical properties of biological components in isolation. Molecules and cells can be manipulated and measured in applied fields using dielectrophoresis, microwave scanning probes, atomic force microscopy, acoustic devices, and optical and magnetic tweezers. Some of the methods use magnetic or electric fields or acoustic fields, others use the EM field radiation pressure, and others use electrostatic and van der Waals forces of attraction [139, 172]. Microfluidic cells together with dc to terahertz EM fields are commonly used to study microliter to picoliter volumes of fluids that contain nanoparticles [173–175]. Surface acoustic waves (SAW) and bulk acoustic waves (BAW) can be used to drive and enhance microfluidic processes. Since there is a difference of wave velocities in a SAW substrate and the fluid, acoustic waves can be transferred into the fluid, to obtain high fluid velocities for separation, pumping, and mixing.

**Fig. 15:**
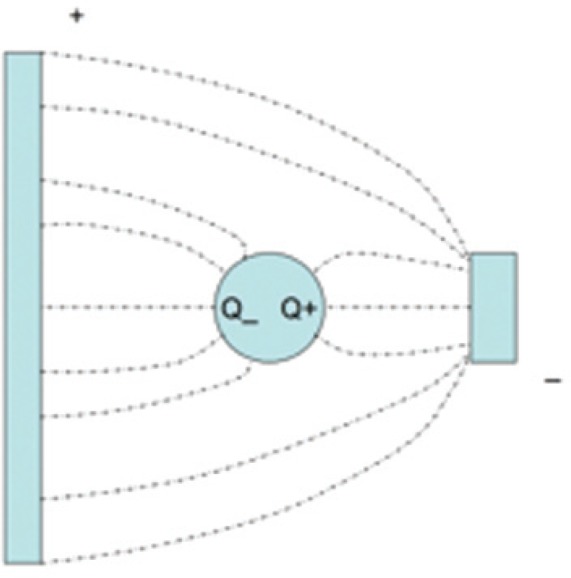
Dielectrophoretic Force.

Due to symmetry and charge neutrality, a polarizable particle in a uniform electric field will experience no net force. If a material with a permanent or induced dipole is immersed in an electric field gradient, then a dielectrophoretic force on the dipole is formed, as indicated in Fig. 15 [176]. In a nonuniformelectric field, the force on a dipole moment **p** is **F** = (**p**·∇)**E**. From this the following equation for the dielectrophoresis force on a small sphere of radius *r* of permittivity ε*_p_* in a background with permittivity ε*_m_* has be derived [177, 178]

(134) $$F_{DEP}=2\pi \varepsilon_{m}r^{3}ℛ\left(\frac{\varepsilon_{p}-\varepsilon_{m}}{\varepsilon_{p}+2\varepsilon_{m}}\right)\nabla {\left|E\right|}^{2}.$$

This force tends to align the molecule along the field gradient. The force is positive if ε*_p_* > ε*_m_*. For dispersive materials, the attraction or repulsive force can be varied by the frequency. Dielectrophoresis is commonly used to stretch, align, move, and determine force constants of biomolecules such as single-stranded and double-stranded DNA and proteins [179]. Dielectrophoresis can also be used to separate cells or molecules in a stream of particles in solution. Usually, dielectrophoretic manipulation is achieved through microfabricated electrodes deposited on chips. For dispersive materials, where the permittivity changes over the frequency band of interest, there is a cross-over frequency where there is no force on the molecule. The approximate force, due to diffusion forces from particle gradients on a particle with a dimension *d*, is *F_b_* = *k_B_T*/*d*. For micrometer particles, the dielectrophoretic field gradients required to overcome this force is not large. However, for nanoscale particles this field gradient is much larger.

**Fig. 16:**
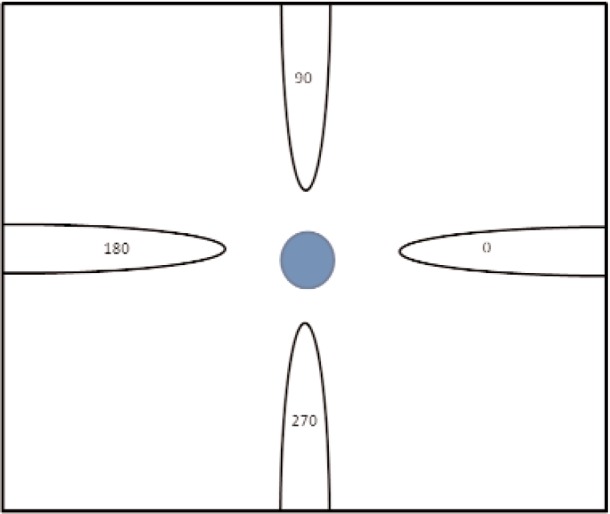
Electrorotation with probes 90° out of phase.

Spherical particles can be made to rotate through electrorotation methods [177]. This motion is produced by a rotating electric field phase around a particle. The dipole induced in the particle experiences a net torque due to the dielectric loss that allows the dipole formation to lag the rotating field, as shown in Fig. 16. The net torque is given by **N** = **p** × **E**. For particles ε*_p_* in a matrix ε*_m_* the torque is [177]

(135) $$N=4\pi \varepsilon_{rm}^{\prime }r^{3}ℛ\left(\frac{\varepsilon_{p}-\varepsilon_{m}}{\varepsilon_{p}+2\varepsilon_{m}}E(\omega )\times E*(\omega )\right).$$

Optical tweezing originates from the EM field gradient obtained from a laser source that produces a field differential and results in a force on particles. This effect is similar to dielectrophoresis. The strength of the radiation pressure on particles is a function of the size of the particles and the wavelength of the laser light [180]. Molecules can also be studied by magnetic tweezers with magnetic-field gradients. By attaching magnetic particles to molecules it is possible to stretch molecules and determine force constants. Opto-plasmonic tweezers use radiation from resonant electrons to create patterned electric fields that can be used through dielectrophoresis to orient nanoscale objects.

Atomic force microscopy (AFM) is based on cantilevers. In AFM the force between the probe tip and the specimen is used to measure forces in the micronewton range. An AFM probe typically has cantilever lengths of 0.2 mm and a width of around 50 μm. An AFM can operate in the contact mode, noncontact, or tapping mode. Force information of the interaction of the tip with a material is obtained by means of cantilever bending, twisting, and, in the noncontact mode, by resonance of the cantilever.

**Fig. 17:**
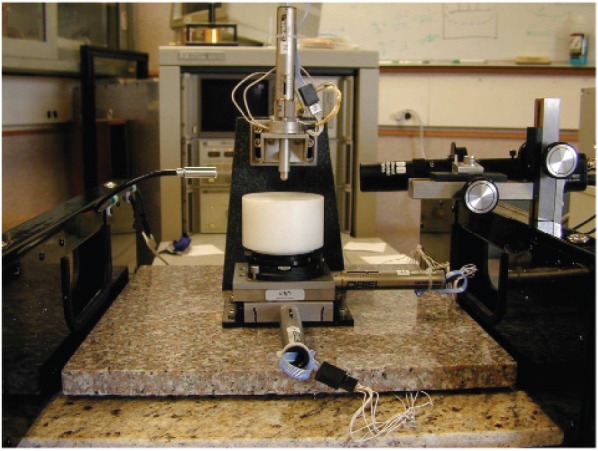
Microwave scanning probe system.

In the microwave range, near-field microwave scanning probes are commonly used. These probes have proved valuable to measure the permittivity and imaging on a surface of a thin film at subwavelength resolution. These needle probes usually use near-field microwaves that are created by a resonator above the probe, as shown in Fig. 17. A shift in resonance frequency is then related to the material properties under test through software based on a theoretical model. Therefore, most of these probes are limited to resonant frequencies of the cavity. Continuous-wave methods based on microstrip tips have also been applied.

### 16.1 Properties and Measurement of Dielectric Nanomaterials

Nanomaterials could consist of composites of nanoparticles dispersed in a matrix or isolated particles. A mixture of conducting nanoparticles dispersed into a matrix sometimes yields interesting dielectric behavior [23, 181]. Lewis has noted that the interface between the nanoparticle and matrix produces unique properties in nanocomposites [23]. Interfaces and surface charges are a dominant parameter governing the permittivity and loss in nanocomposites [23, 181, 182]. Double layers (Sec. 8) near the particle surface can strongly influence the properties [23]. In addition, conductivity in some nanoparticles can achieve ballistic transport.

In order to model a single dielectric nanoparticle in an applied field the local field can be calculated, as summarized in Sec. 4.3. Kühn et al. [59] studied the local field around nanoparticles, and they found that use of the macroscopic field for modeling of a sphere containing nanoparticles was not valid at below 100 nm. In order to model small groups of nanoparticles, they found that the effects of the interface required the use of local fields rather than the macroscopic field.

When individual nanoparticles are subjected to EM fields, the question arises of whether it is possible to define a permittivity of the nanoparticle or whether an ensemble of particles is required. Whether permittivity of a nanoparticle is well defined depends on the number of dipole moments within the particle. If we use the analogy of a gas, we assume that the large number of gas molecules together with the vacuum around the particles constitutes a bulk permittivity. This permittivity does not apply to the individual gas molecules, but rather to the bulk volume. When individual nanoparticles contain thousands of dipoles, according to criteria of permittivity developed in Sec. 4.6, long-wavelength fields would allow defining a permittivity of the particle and a macroscopic field. However, such a permittivity would be spatially varying due to interfacial effects, and the definition would break down when there are insufficient particles to perform an ensemble average [59].

### 16.2 Electrical Properties and the Measurement of Nanowires

Nanowires are effectively one-dimensional entities that consist of a string of atoms or molecules with a diameter of approximately 10^–9^ meters. Nanowires may be made of TiO_2_, SiO_2_, platinum, semiconducting compounds such as gallium nitride and silicon, single (SWNT) or multi-wall (MWNT) carbon nanotubes, and inorganic and organic strings of molecules such as DNA [183–188]. Because they are effectively ordered in one dimension, they can form a variety of structures such as rigid lines, spirals, or zigzag pattern. Carbon nanotubes that have lengths in the millimeters have been constructed [189].

At these dimensions, quantum-mechanical effects cannot be totally neglected. For example, the electrons are confined laterally, which influences the available energy states like a particle in a one-dimensional box. This causes the electron transport to be quantized and therefore the conductance is also quantized (2*e*^2^/*h*). The impedance of nanoconductors is on the order of the quantum resistance *h*/*e*^2^, which is 25 kΩ. For SWNTs, due to band-structure degeneracy and spin, this is reduced to 6 kΩ. The ratio of the free-space impedance to the quantum impedance is two times the fine structure constant 2α. This high impedance is difficult to probe with 50 Ω systems [190], and depositing a number of them in parallel has been used to minimize the mismatch [191].

The resistance of a SWNT depends on the diameter and chirality. The chirality is related to the tube having either metallic or semiconducting properties. For device applications such as nanotransistors, the nanowires need to be either doped or intrinsic semiconductors. Semiconducting nanowires can be connected to form p-*n* junctions and transistors [192].

Many nanowires have a permanent dipole moment. Due to the torque in an electric field, the dipole will tend to align with the field, particularly for metallic and semiconducting nanotubes [193].

### 16.3 Charge Transport and Length Scales

Electrical conduction through nanowires is strongly influenced by their small diameter. This constriction limits the mean free path of conduction electrons [88, 194]. For example in bulk copper the mean free path is 40 nm, but nanowires may be only 1 to 10 nm in diameter, which is much less than a mean free path and results in constriction of the current flow.

Carbon nanotubes can obtain ballistic charge transport. Ballistic transport is associated with carrier flow without scattering. This occurs in metallic nanowires when the diameter becomes close to the Fermi wavelength in the metal. The electron mean-free path for a relaxation time τ*_e_* is *l_e_* = ντ*_e_*, and if *l_e_* is much larger than the length of the wire, then it is said to exhibit ballistic transport. Carbon nanotubes can act as antennas and can have plasmonic resonances in the low terahertz range.

The Landauer-Buttiker model of ballistic transport was developed for one-dimensional conduction of spinless/noninteracting electrons [195, 196]. This model has been applied to nanowires.

Graphene has shown promise for construction of transistors due to its high conductivity, but is hampered by defects. The very high carrier mobility of graphene makes it a candidate for very high speed radiofrequency electronics [197].

### 16.4 Distributed Parameters and Quantized Aspects

A high-frequency nanocircuit model may need to include the quantum capacitance and kinetic and magnetic inductance in addition to the classical parameters. The magnetic inductance per unit length for a nanowire of permeability *μ* of diameter *d* and a distance *s* over a ground plane is given by [189] ${ℒ}_{M}=\frac{\mu }{2\pi }\cosh^{-1}(2s/d)\approx \frac{\mu }{2\pi }\ln\frac{s}{d}$, typically1(pH/*μ*m). The kinetic inductance due to quantum effects is is related to the Fermi velocity *ν_F_*, ${ℒ}_{K}=\frac{h}{2e^{2}\nu_{F}}$, typically, 16 (nH/*μ*m). At gigahertz frequencies, the kinetic inductance is not a dominant contribution to the transmission line properties [189]. The electrostatic capacitance between a wire and ground plane in a medium with permittivity ε is $C_{ES}=\frac{2\pi \varepsilon }{\cosh^{-1}(2s/d)}$, typically, 50 (aF/*μ*m)*.* The quantum capacitance is $C_{Q}=\frac{8e^{2}}{h\nu_{F}}$, typically, 400 (aF/*μ*m). The electrostatic capacitance is found to dominate over the quantum capacitance at gigahertz frequencies. At terahertz frequencies and above they are of the same order of magnitude, and both should be included in calculations for nanowires. Burke notes that the resistance and classical capacitance dominates over the quantum inductance and capacitance and are not important contributions at gigahertz frequencies, but may be important at terahertz frequencies [189]. The wave velocity in nanowires is approximated by

(136) $$\nu_{F}\approx \frac{1}{\sqrt{{ℒ}_{K}C_{Q}}}.$$

The quantum characteristic impedance is

(137) $$Z=\sqrt{\frac{ℒ}{C}}=\frac{h}{2e^{2}}.$$

If the noninteracting electrostatic and quantum impedance are combined, we have

(138) $$Z=\sqrt{({ℒ}_{K}+{ℒ}_{M})\left(\frac{1}{C_{Q}}+\frac{1}{C_{ES}}\right)}.$$

Whereas the free-space impedance is 377 Ω, the quantum capacitance and inductance of carbon nanotubes yields an impedance of approximately 12.5 kΩ.

The resistivity of nanowires and copper are generally of the same order of magnitude. The ballistic transport properties at small scales represents an advantage; however, the resistance is still quite high. Copper interconnects have less resistance until the conductor sizes drop below about 100 nm; currently the microelectronic industry uses conductors of smaller size. This is an origin of heating [14, 198]. Because the classical resistance is calculated from *R*/*L* = ρ/*A*, where ρ is resistivity, *L* is length, and *A* is the cross-sectional area, the small area of a SWNT limits the current and increases the resistance per unit length and the impedance. Due to the high impedance of nanowires, single nanowires have distinct disadvantages; for example, carbon nanotubes may have impedances on the order of 10^4^ Ω. Bundles of parallel nanowires could form an interconnect [191]. Tselev et al. [191] performed measurements on bundles of carbon nanotubes that were attached to sharp metal tips by dielectrophoresis on silicon substrates. Electron-beam lithography was used to attach conductors to the tubes. High-frequency inductance measurements from 10 MHz to 67 GHz showed that the inductance was nearly independent of frequency. In modeling nanoscale antennas made from nanowires, the skin depth as well as the resistance are important parameters [189].

## 17. Random Fields, Noise, and Fluctuation-Dissipation Relations

### 17.1 Electric Polarization and Thermal Fluctuations

As transmission lines approach dimensions of tens of nanometers with smaller currents, thermal fluctuations in charge motion can produce small voltages that can become a significant source of noise [199]. The random components of charge currents, due to brownian motion of charges, produce persistent weak random EM fields in materials and produces a flow of noise power in transmission lines. These fields contribute to the field felt by the device. Random fields also are important in radiative transfer in blackbody and non-blackbody processes.

Thermal fluctuations in the dipole moments in dielectric and magnetic materials influence the polarization and are summarized in the well known fluctuation-dissipation relationships. These relationships are satisfied for equilibrium situations. Equilibrium is a state where the entropy is a maximum and macroscopic quantities such as temperature, pressure, and local fields are well defined. Fluctuation-dissipation relationships can be obtained from the linear-response formalism (Sec. 4.4) that yields the susceptibility in terms of the Fourier transform of the associated correlation functions. By use of Eq. (30), an expression can be written for the susceptibility in terms of the polarization

(139) $$\begin{array}{l} {\overset{↔}{\chi }}_{e}^{\prime \prime }(\omega )=\int_{0}^{\infty }{\overset{↔}{f}}_{e}(t)\sin(\omega t)dt\\ \;=-\frac{V}{k_{B}T}\int_{0}^{\infty }\frac{d}{dt}(<P(0)P(t)>)\sin(\omega t)dt\\ \;=\frac{\omega V}{2k_{B}T}\int_{-\infty }^{\infty }<P(0)P(t)>\cos(\omega t)dt. \end{array}$$

Equation (139) is a fluctuation-dissipation relationship that is independent of the applied field. In this approach, if the correlation function is known, then the material properties can be calculated. However, in practice most material properties are measured through applied fields. The interpretation of this relationship is that the random microscopic electric fields in a polarizable lossy medium produce fluctuations in the polarization and thereby induces loss in the decay to equilibrium. These fluctuations can be related to entropy production [44, 61]. We can obtain an analogous relation for the real part of the susceptibility by use of Eq. (29). This relation relates the real part of the susceptibility to fluctuations

(140) $$\begin{array}{l} \overset{↔}{\chi_{e}^{\prime }}(\omega )=\int_{0}^{\infty }\overset{↔}{f_{e}}(t)\cos(\omega t)dt\\ \;=\frac{V}{k_{B}T}\int_{0}^{\infty }\frac{d}{dt}(<P(0)P(t)>)\cos(\omega t)dt\\ \;=\frac{\omega V}{k_{B}T}\int_{-\infty }^{\infty }<P(0)P(t)>\sin(\omega t)dt. \end{array}$$

### 17.2 Magnetic Moment Thermal Fluctuations

Magnetic-moment fluctuations with respect to signal-to-noise limitations are important to magnetic-storage technology [200]. This noise can also be modeled by fluctuation-dissipation relations for magnetic response. The linear fluctuation-dissipation relation for the magnetic loss component can be derived in a way similar to the electric response:

(141) $$\begin{array}{l} \overset{↔}{\chi_{m}^{\prime \prime }}(\omega )=\int_{0}^{\infty }\overset{↔}{f_{m}}(t)\sin(\omega t)dt\\ \;=-\frac{V\mu_{0}}{k_{B}T}\int_{0}^{\infty }\frac{d}{dt}(<M(0)M(t)>)\sin(\omega t)dt\\ \;=\frac{\omega V\mu_{0}}{2k_{B}T}\int_{-\infty }^{\infty }<M(0)M(t)>\cos(\omega t)dt. \end{array}$$

### 17.3 Thermal Fields and Noise

Due to thermal fluctuations, brownian motion of charges produce random EM fields and noise. In noise processes the induced current density can be related to microscopic displacement $\vec{D}$ and induction fields $\vec{ℬ}$.

The cross-spectral density of random fields is defined as [18]

(142) $$S_{E_{kl}}(r,r^{\prime },\omega )=\int_{-\infty }^{\infty }<E_{k}(r,t)E_{l}(r^{\prime },t^{\prime })>e^{-i\omega (t-t^{\prime })}d(t-t^{\prime }).$$

The relationship to the time-harmonic correlation function for the field components is

(143) $$<E_{k}(r,\omega )E_{l}^{*}(r^{\prime },\omega^{\prime })>=2\pi S_{E_{kl}}(r,r^{\prime },\omega )\delta (\omega -\omega^{\prime }).$$

Thermally induced fields can be spatially correlated [17] and can be modeled to first order as

(144) $$<\vec{D}(\omega ,r){\vec{D}}^{*}(\omega ,r^{\prime })>=\frac{2i\Theta (\omega ,T)}{\omega }(\overset{↔}{\varepsilon }-{\overset{↔}{\varepsilon }}^{*})\delta (r-r^{\prime }),$$

(145) $$<\vec{ℬ}(\omega ,r){\vec{ℬ}}^{*}(\omega ,r^{\prime })>=\frac{2i\Theta (\omega ,T)}{\omega }(\overset{↔}{\mu }-{\overset{↔}{\mu }}^{*})\delta (r-r^{\prime }),$$

(146) $$<\vec{ℬ}(\omega ,r){\vec{D}}^{*}(\omega ,r^{\prime })>=0,$$

where Θ(ω, *Τ*) = (*ħ*ω/2)coth(*ħ*ω/2*k_B_T*). Θ → *k_B_T* for *k_B_T* ≫ *ħ*ω.

The voltage *V* and current *I* in a microscopic transmission line with distributed noise sources ν*_n_* and *i_n_* that are caused by random fields can be modeled by coupled differential equations as shown in [199].

A special case of Eq. (144) is the well-known Nyquist noise relation for voltage fluctuations from a resistance *R* over a bandwidth Δ*f* is

(147) $$<\nu^{2}>=4k_{B}TR\Delta f.$$

### 17.4 Fluctuations and Entropy

#### 17.4.1 Fluctuations

In thermal equilibrium macroscopic objects have a well-defined temperature, but in addition there are equilibrium temperature fluctuations. When the particle numbers in a system decrease, the thermodynamic quantities such as temperature and internal energy, have a less precise meaning than in a large-scale system [61, 201]. In nanosystems, fluctuations in particle energy, momentum, and local EM fields can be large enough to affect measurements. These fluctuations translate into fluctuations in the measured EM fields, internal energy, temperature, and heat transfer. A system that is far from thermal equilibrium or very small may not have a well-defined temperature, macroscopic internal energy, or specific heat [199, 202, 203]. When the applied driving fields are removed, some polymers and some spin systems have relaxation times of seconds to hours until they decay from a nonequilibrium state to an equilibrium state. In these types of nonequilibrium relaxation processes, equilibrium parameters such as temperature have only a fuzzy meaning. Fluctuation-dissipation relations that are used to define transport coefficients in equilibrium do not apply out of equilibrium.

Nanosystems operate in the region between quantum-mechanical and macroscopic description and between equilibrium and nonequilibrium states. Whereas Johnson noise is related to fluctuations in equilibrium voltages, there is a need for theoretical work that yields results that compare well to measurements in this transition region. As an example, Hanggai et al. showed that the theoretical bulk definitions for specific heat and entropy in some nanosystems break down in the high or low temperature limits [204]. Noise also occurs in non-equilibrium systems and the theoretical foundations are not as well developed as in thermal equilibrium.

#### 17.4.2 Fluctuations and Entropy Production

For reliable operation, microelectronic interconnects require a stable thermal environment because thermal fluctuations could potentially damage an interconnect or nano-transistor [205]. An understanding of thermodynamics at the nanoscale and the merging of electromagnetism and non-equilibrium thermodynamics is important for modeling small systems of molecules. Modeling of thermal fluctuations can be achieved by relating Nyquist noise to fluctuations in thermal energy. Another approach away from equilibrium is to use the concept of entropy production [44]. Entropy can be increased either by adding heat to a material, Δ*S* = Δ*Q_h_*/*T*, or by spontaneous processes in the relaxation of a system from nonequilibrium to an equilibrium state. In EM interaction with materials, we can produce entropy either through the dissipation of the fields in the material or by relaxation processes. Relaxation processes are usually spontaneous processes from nonequilibrium into an equilibrium state.

The entropy is defined as *S* = *k_B_* ln(*W*), where *W* is the number of accessible states. Entropy is a cornerstone of thermodynamics and non-equilibrium thermodynamics. In thermodynamics the free energy is defined in terms of the internal energy *U* as ℱ = − *k_B_T* ln*Z*, where *Z* is the partition function. The entropy is also defined in terms of the free energy as

(148) $$S(T)=-\frac{\partial ℱ}{\partial T}.$$

In thermodynamics, temperature is defined as

(149) $$\frac{\delta S(T)}{\delta U}=\frac{1}{T}.$$

A very general evolution relation for the macroscopic entropy production rate Σ(*t*) in terms of microscopic entropy production rate $\dot{s}(t)$ was derived from first principles by use of a statistical-mechanical theory [19, 44, 61, 89, 206]:

(150) $$\Sigma (t)=\frac{1}{k_{B}}\int_{0}^{1}<\dot{s}(t)T(t,\tau )(1-P(\tau ))\dot{s}()>d\tau ,$$

where $\dot{s}$(*t*) satisfies $\left(<\dot{s}(t)>=0\right)$, Σ(*t*) is the net macroscopic entropy production in the system, and *T* and *P* are evolution operators and projection operators, respectively. The Johnson noise formula is a special case of Eq. (150) near equilibrium, when Σ(*t*) = *I*^2^*R*/*T* and $\dot{s}(t)=(1/2)I\nu (t)/T$, (with < ν(*t*) > = 0) is a fluctuating voltage variable, and *I* is a bias current.

## 18. Dielectric Response of Crystalline, Semiconductors, and Polymer Materials

### 18.1 Losses in Classes of Single Crystals and Amorphous Materials

A class of dielectric single-crystal materials have very low loss, especially at low temperatures. The low loss is related to the crystal order, lack of free charge, and the low number of defects. Anomalously low values of the dielectric loss in single-crystal alumina at low temperatures were reported in 1981 [14, 207]. In this study, dielectric resonators were used to measure the loss tangent because cavity resonators do not have the required precision for very-low-loss materials. Since then, there has been a large body of research [208, 209] performed with dielectric resonators that supports these results. Braginsky et al. [207] showed that the upper bound for loss in high-quality sapphire was 1.5 × 10^−9^ at 3 GHz and at *T* = 2 K. These reports were supported by Strayer et al. [210]. These results are also consistent with the measurements by Krupka et al. [209], who used a whispering-gallery mode device to measure losses. Very low loss is obtained in sapphire, diamond, single-crystal quartz, MGO, and silicon. Low loss resonators have been studied at candidates for frequency standards.

The whispering-gallery mode technique is a particularly accurate way of measuring the loss tangent of materials with low loss [14]. These researchers claim that the loss tangent for many crystals follows roughly a *f*^2^ dependence at low temperatures.

In nonpolar materials, dielectric loss originates from the interaction of phonons or crystal oscillations with the applied electric field. In the absence of an applied electric field, the lattice vibrates nearly harmonically and there is little phonon-phonon interaction. The electric-field interaction modifies the harmonic elastic constant and thereby introduces an anharmonic potential term. The anharmonic interaction allows phonon-phonon interaction and thereby introduces loss [73]. Some of the scattering of phonons by other phonons is manifested as loss.

The loss in many crystals is due to photon quanta of the electric field interacting with phonons vibrating in the lattice, thereby creating a phonon in another branch. Dielectric losses originate from the electric field interaction with phonons together with two-, three-, and four-phonon scattering and Umklapp process [73]. The three- and four-quantum loss corresponds to transitions between states of the different branches. Crystals with a center of symmetry have been found to generally have lower loss than ones with noncentrosymmetry. The temperature dependence also depends on the crystal symmetry. For example, a symmetric molecule such as sapphire has much lower loss than noncentro-symmetric ferroelectric crystals such as strontium barium titanate. Quasi-Debye losses correspond to transitions, which take place between the same branch. In centro-symmetric crystals three- and four-quantum processes are dominant. In noncentro-symmetric crystals the three-quantum and quasi-Debye processes dominate.

Gurevich and Tagantsev [73] studied the loss tangent for cubic and rhombohedric symmetries for temperatures far below the Debye temperature *T_D_* = 1047 K. For these materials, the loss tangent can be modeled as

(151) $$\tan\delta =\frac{\omega^{2}{\left(k_{B}T\right)}^{4}}{\varepsilon \rho \nu^{5}\hbar {\left(k_{B}T_{D}\right)}^{2}},$$

where ε is permittivity, ρ is density, ν is speed of sound in air. For hexagonal crystals, without a center of symmetry,

(152) $$\tan\delta =\frac{\omega {\left(k_{B}T\right)}^{3}}{\varepsilon \mu \nu^{5}\hbar },$$

and with symmetry,

(153) $$\tan\delta =\frac{\omega {\left(k_{B}T\right)}^{5}}{\varepsilon \mu \nu^{5}\hbar^{2}{\left(k_{B}T_{D}\right)}^{2}}.$$

For many dielectric materials with low loss, Gurevich showed that there is a universal frequency response of the form tan δ ∝ ω.

The loss tangent in the microwave band of many low-loss ceramics, fused silica, and many plastics and some glasses increases nearly linearly as frequency increases [211]. For materials where the loss tangent increases linearly with frequency, we can interpolate and possibly extrapolate microwave loss-tangent measurement data from one frequency range to another (Fig. 6). This approach is, of course, limited. This behavior can be understood in terms of Gurevich’s relaxation models [73] or by the moment expansion in [212].

This behavior is in contrast to the model of Jonscher [213] who has stated that χ″/χ′ is nearly constant with frequency in many disordered solids.

### 18.2 Electric Properties of Semiconductors

**Fig. 18:**
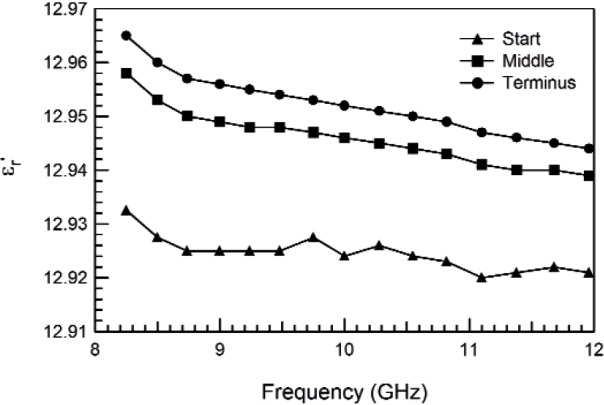
Relative permittivity $\varepsilon_{r}^{\prime }$ of gallium arsenide measured by an X-band cavity [218]. Start, middle, and terminus refer to different specimens taken from the same boule. For these measurements the Type B expanded relative uncertainty at 10 GHz in $\varepsilon_{r}^{\prime }$ was *U* = *ku_c_* = 0.02 (*k* = 2), where *k* is coverage factor.

Excellent reviews of the dielectric properties of semiconductors in the microwave range have been given by Jonscher and others [14, 213–217]. The dc conductivities of semiconductors are related to holes and free charge. In the gigahertz region, the total loss in most semiconductors decreases significantly since the effects of the dc conductivity decreases; however, the dielectric component of loss increases. For gallium arsenide and gallium nitride the conductivity is relatively low. Figure 18 shows measurement results on the permittivity of high-resistivity gallium arsenide as a function of frequency. These measurements were made by a mode-filtered TE_01_ X-band cavity. Silicon semiconductors can exhibit low to high loss depending on the level of dopants in the material. There are Schottky barriers at the interface between semiconductors and metals and at p-n junctions that produce losses.

The conductivities of semiconductors at low frequencies fall between those of metals and dielectrics. The theory of conductivity of semiconductors begins with an examination of the phenomena in intrinsic (undoped) samples. At temperatures above 0 K, the kinetic (thermal) energy becomes sufficient to excite valence band electrons into the conduction band, where an applied field can act upon them to produce a current. As these electrons move into the conduction band, holes are created in the valence band that effectively become another source of current. The total expression for the conductivity includes contributions from both electrons and holes and is given by σ*_dc_* = *q* (*nμ_n_* + *pμ_p_*), where *q* is charge, *n* is the electron density, *p* is the hole density, and *μ_n_* and *μ_p_* are the electron mobility and hole mobility, respectively.

In intrinsic semiconductors, the number of charge carriers produced through thermal excitation is relatively small, but σ*_dc_* can be significantly increased by doping the material with small amounts of impurity atoms. These additional carriers require much less thermal energy in order to contribute to σ*_dc_*. This results in more carriers becoming available as the temperature increases, until ionization of all the impurity atoms is complete.

For temperatures above the full ionization range of the dopants, σ*_dc_* is increasingly dominated by *μ_n_* and *μ_p_*. In semiconductors such as silicon, the mobility of the charge carriers decreases as the temperature increases, due primarily to the incoherent scattering of the carriers with the vibrating lattice. At a temperature *T_i_*, intrinsic effects begin to contribute additional charge carriers beyond the maximum contributions of the impurity atoms, and σ*_dc_* begins to increase again [215, 216, 219–222].

## 19. Overview of the Interaction of RF Fields With BiologicalMaterials

### 19.1 RF Electrical Properties of Cells, Amino Acids, Peptides, and Proteins

In this section, we will overview the dielectric relaxation of cells, membranes, proteins, amino acids, and peptides [97, 223–229]. This research area is very large and we summarize only the most basic concepts as they relate to RF fields.

Dielectric response of biological tissues to applied RF fields is related to membrane and cell boundaries, molecular dipoles, together with associated ionic fluids and counterions [230]. The ionic solution produces low-frequency losses that are very high. As a consequence of these mobile charge carriers, counterions adhere to molecular surfaces, interface charge causes Maxwell-Wagner capacitances, and electrode polarization is formed at electrode interfaces. All of these processes can yield a very high effective $\varepsilon_{r}^{\prime }$ at low frequencies. Some of the effects of the electrodes can be corrected for by use of standard techniques [230, 231] (Sec. 8).

Some biological tissues exhibit an α relaxation in the 100 Hz to 1 kHz region due to dipoles and Maxwell-Wagner interface polarization, another β relaxation in the megahertz region due to bound water, and γ relaxation in the microwave region due to the relaxation of water and water that is weakly bond.

Amino acids contain carboxyl (COOH) groups, amide (NH_2_) groups, and side groups. The side groups and the dipole moment of the amino and carboxyl groups determine most of the low-frequency dielectric properties of the acid. Some of the side groups are polar, while others are nonpolar. When ionized, the amino and carboxyl groups have positive and negative charges, respectively. This charge separation forms a permanent dipole (Fig. 5). α amino acids have an amino group and carboxyl group on the same carbon denoted *C*_α_ and α-amino acids have a dipole moment of 15 to 17 debyes (D) (1 debye equals 3.33 × 10^−30^ coulomb-meter). β amino acids have a *CH*_2_ group between the amino and carboxyl groups, which produces a large charge separation and therefore a dipole moment on the order of 20 D. For a very good overview see Pethig [223]. Peptides are formed from condensed amino acids. A peptide consists of a collection of amino acids connected by peptide bonds. Peptide bonds provide connections to amino acids through the CO-NH bond by means of the water molecule as a bridge. The peptide unit has a dipole moment on the order of 3.7 D. Chains of amino acids are called polyamino acids or polypeptides. These are terminated by an amide group on one end and a carboxyl group on the other side. Typical dipole moments for polypeptides are on the order of 1000 D.

Polyamino acids can be either in the helical or random-coil phase. In the helical state, C = O bonds are linked by hydrogen bonds to NH groups. The helix can either be right-handed or left-handed; however, the right-handed helix is more stable. Generally, polyamino acids have permanent dipole moments and dielectric relaxation frequencies in the kilohertz region [232].

The origin of relaxation in proteins has been debated over the years. Proteins are known to be composed of polyamino acids with permanent dipole moments, but they also have free and loosely bound protons. These protons bind loosely to the carboxyl and amino groups. Kirkwood et al. hypothesized that much of the observed relaxation behavior of proteins is due to movement of these nearly free protons in the applied field or the polarization of counterion sheaths around molecules [233]. Strong protonic conductivity has also been observed in DNA. At present, the consensus is that polar side chains and both permanent dipoles and the proton-induced polarization contribute to dielectric relaxation of proteins.

In the literature three dielectric relaxations in proteins have been identified [231]. These are similar to that in DNA. The first is the α relaxation in the 10 kHz to 1 MHz region and is due to rotation of the protein side chains. The second minor β relaxation occurs in the 100 MHz to 5 GHz range and is thought to be due to bound water. The third γ relaxation is around 5 GHz to 25 GHz and is due to semi-free water.

Nucleic acids are high-molecular mass polymers formed of pyrimidine and purine bases, a sugar, and phosphoric-acid backbone. Nucleic acids are built up of nucleotide units, which are composed of sugar, base, and phosphate groups in helical conformation. Nucleotides are linked by three phosphates groups, which are designated α, β, and γ. The phosphate groups are linked through the pyrophosphate bond. The individual nucleotides are joined together by groups of phosphates that form the phosphodiester bond between the 3′ and 5′ carbon atoms of sugars. These phosphate groups are acidic. Polynucleotides have a hydroxyl group at one end and a phosphate group on the other end. Nucleosides are subunits of nucleotides and contain a base and a sugar. The bond between the sugar and base is called the glycosidic bond. The base can rotate only in the possible orientations about the glycosidic bond.

Watson and Crick concluded through x-ray diffraction studies that the structure of DNA is in the form of a double-stranded helix. In addition to x-ray structure experiments on DNA, information has been gleaned through nuclear magnetic resonance (NMR) experiments. Types A and B DNA are in the form of right-handed helices. Type Z DNA is in a left-handed conformation. There is a Type B to Z transition between conformations. A transition from Type A to Type B DNA occurs when DNA is dissolved in a solvent [234]. The Watson-Crick conception of DNA as a uniform helix is an approximation. In reality, DNA exists in many conformations and may contain inhomogeneities such as attached proteins. In general, double-stranded DNA is not a rigid rod, but rather a meandering chain. Once formed, even though the individual bonds composing DNA are weak, the molecule as a whole is very stable. The helical form of the DNA molecule produces major and minor grooves in the outer outer surface of the molecule. There are also boundwater molecules in the grooves. Many interactions between proteins or protons with DNA occur in these grooves.

The helix is formed from two strands. The bases in adjacent strands combine by hydrogen bonding, an electrostatic interaction with a pyrimidine on one side and purine on the other. In DNA, the purine adenine (A) pairs with the pyrimidine thymine (T). The purine guanine (G) pairs with the pyrimidine cytosine (C). A hydrogen bond is formed between a covalently bonded donor hydrogen atom that is positively charged and a negatively charged acceptor atom. The A-T base pair associates by two hydrogen bonds, whereas C-G base pairs associate by three hydrogen bonds. The base-pair sequence is the carrier of genetic information. The genetic code is formed of a sequence of three base pairs, which determine a type of amino acid. For example, the sequence of $\underset{phe}{\underset{︸}{TTT}}\underset{lyso}{\underset{︸}{AAA}}\underset{lys}{\underset{︸}{AAG}}\underset{ala}{\underset{︸}{GCT}}$ determines an amino acid sequence of phenylalanine-lysine-lysine-alanine.

The DNA molecule has a net negative charge due to the phosphate backbone. When dissolved in a cation solution, some of the charge of the molecule is neutralized by cations. The double-stranded DNA molecule is generally thought to have little intrinsic permanent dipole moment. This is because the two strands that compose the helix are oriented so that the dipole moment of one strand cancels the other. However, when DNA is dissolved in a solvent, such as saline solution, an induced dipole moment forms due to reorganization of charge into a layer around the molecule called the counterion sheath.

The interaction of the counterions with biomolecules has been a subject of intensive research over the years. Some of the counterions bind to the phosphate backbone with a weak covalent bond. Other counterions are more loosely bound and some may penetrate into the major and minor grooves of DNA [235]. Ions are assumed to be bound near charges in the DNA molecule, so that a double layer forms. The ions attracted to the charged DNA molecule forms a counterion sheath that shields some of the charge of the DNA. The counterion sheath around a DNA molecule is composed of cations such as Na or Mg, which are attracted to the backbone negative phosphate charges. These charges are somewhat mobile and oscillate about phosphate charge centers in an applied electric field. A portion of these counterions is condensed near the surface of the molecule, whereas the vast majority are diffusely bound. Double-stranded DNA possesses a large induced dipole moment on the order of thousands of debye, due to the counterion atmosphere. This fact is gleaned from dielectric relaxation studies, birefringence, and dichroism experiments [236], and other light-scattering experiments [237]. The induced dipole moment $\vec{\mu }$ in an electric field **E** is defined in terms of the polarizability $\vec{\mu }=\alpha E$.

Because the individual strands of double-stranded DNA are antiparallel and the molecule is symmetrical, the transverse dipole moments should cancel. However, a number of researchers have measured a small permanent dipole moment for DNA [238]. In alternating fields, the symmetry of the molecule may be deformed slightly to produce a small permanent dipole moment [231]. Another origin of the small permanent dipole moment is attached charged ligands such as proteins or multivalent cations [239]. These ligands produce a net dipole moment on the DNA molecule by breaking the symmetry. The question of how much of the relaxation of the DNA molecule is due to induced dipolemoment versus permanent moment has been studied by Hogan et al. [236]. The response of permanent vs. induced dipole moment differs in terms of field strength. The potential energy of a permanent dipole moment at an angle θ to the electric field is *U* = − *μ E* cos θ, whereas the induced dipole moment in the electric field is quadratic, *U* = − (Δα/2)*E*^2^ cos^2^ θ, where Δα is the difference in polarizability along anisotropy axes of the molecule. Experiments indicate that the majority of the moment was induced rather than permanent. Charge transport through DNA can be ballistic.

### 19.2 Dielectric Properties of BoundWater and Polyelectrolytes

Knowledge of the permittivity of the water near the surface of a biomolecule is useful for modeling. The region close to a biomolecule in water has a relatively low real part of permittivity and a fixed charge. The region far from the molecule has a permittivity close to that of water. Lamm and Pack [240] studied the variation of permittivity in the grooves, near the surface, and far away from the DNA molecule. The effective permittivity depends on solvent concentration, distance from the molecule, the effects of the boundary, and dielectric field-saturation. The variation of permittivity with position significantly alters the predictions for the electric potential in the groove regions. Model predictions depend crucially on knowing the dielectric constant of water. Numerical modeling of the DNA molecule depends critically on the permittivity of water. When the permittivity of water varies in space, numerical models indicate that small ions such as hydrogen can penetrate into the minor and major grooves [235, 241]. These predictions are not obtained for models that use spatially independent permittivity for water. From modeling results it was found that the real part of the effective permittivity around the DNA molecule varies as a function of distance from the center of the molecule and as a function of solvent concentration in moles per liter (mol/l) [240].

The molecular structure of water is not simple. Besides the basic H_2_O triad structure of the water molecule, there are also complicated hydrogen-bonded networks created by dipole-dipole interactions that form hydroxyl OH^−^ and hydronium H_3_O^+^ ions. The dielectric constant of water at low frequencies is about 80, whereas biological water contains ions, which affect both the real and imaginary parts of the permittivity. Water bound in proteins and DNA has a decreased permittivity. This is due to constraints on the movement of the molecules when they are attached to biomaterials.

### 19.3 Response of DNA and Other Biomolecules in Electric Driving Fields

**Fig. 19:**
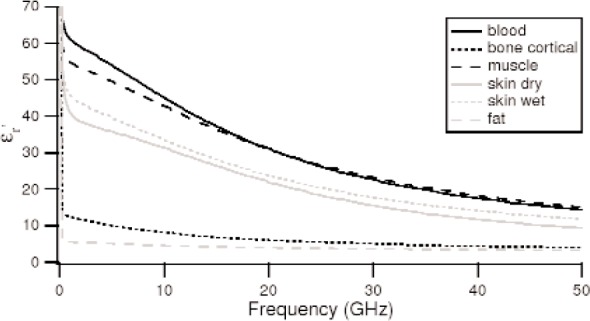
Measurements of the relative permittivity of various body tissues by Gabriel et al. [242] (no uncertainties assigned).

**Fig. 20:**
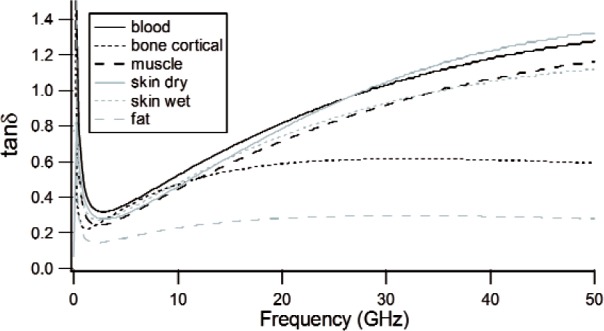
Loss tangent of human tissues by Gabriel et al. [242].

The low-frequency response of DNA is due primarily to longitudinal polarization of the diffuse counterion sheath that surrounds the molecule. This occurs at frequencies in the range of 1 to 100 Hz. Another relaxation occurs in the megahertz region due to movement of condensed counterions bound to individual phosphate groups. Dielectric data on human tissue is given in Figs. 19 and 20. A number of researchers have studied dielectric relaxation of both denatured and helical conformation DNA molecules in electrolyte solutions both as a function of frequency and applied field strength. Single-stranded DNA exhibited less dielectric relaxation than double-stranded DNA [98, 243–246]. Takashima concluded that denatured DNA tended to coil and thereby decrease the effective length and therefore the dipole moment. Furthermore, a high electric field strength affects DNA conductivity in two ways [244]. First, it promotes an increased dissociation of the molecule and thereby increases conductivity. Second, it promotes an orientation field effect where alignment of polyions increases conductivity.

There are many other types of motion of the DNA molecule when subjected to mechanical or millimeter or terahertz electrical driving fields. For example, propeller twist occurs when two adjacent bases in a pair twist in opposite directions. Another motion is the breather mode where two bases oscillate in opposition as hydrogen bonds are compressed and expanded. The Lippincott-Schröder and Lennard-Jones potentials are commonly used for modeling these motions. These modes resonate at wavelengths in the millimeter region; however, relaxation damping prevents direct observation. Other static or dynamic motions of the base pairs of the DNA molecule are roll, twist, and slide.

Single-stranded DNA, in its stretched state, possesses a dipole moment oriented more or less transverse to the axis. The phosphate group produces a permanent transverse dipolemoment of about 20 D per 0.34 nm base-pair section. The Debye (D) is a unit of dipole moment and has a value of 3.336 × 10^−30^ C · m. Because the typical DNA molecule contains thousands of base pairs, the net dipole moment can be significant. However, as the molecule coils or the base pairs twist, the dipole moment decreases. If single strands of DNA were rigid, since there is a transverse dipole moment, and relaxation would occur in the megahertz to gigahertz frequencies.

### 19.4 Dynamics of Polarization Relaxation in Biomaterials

In order to study relaxation of polypeptides and DNA in solution, we first consider the simplest model of a dipolar rigid rod.

The torque on an electric dipole moment **p** is

(154) N=p×E.

For cases where the dipole moment is perpendicular to the rod axis, rotations about the major axis can occur. The longitudinal rotation relaxation time for a molecule of length *L* is given in [247]. The relaxation time varies with the molecule length. Major axis rotation could occur if the molecule had a transverse dipole moment; for example, in a single strand of DNA.

When the dipole moment is parallel to the major axis, end-over-end rotation may occur. This is the type of relaxation at low frequencies that occurs with the induced dipole moment in the counterion sheath or a permanent dipole moment parallel to the longitudinal axis of the molecule. The relaxation time varies as *L*^3^. Because length of the molecule and molecular mass are related, the responses for the two relaxations depend on molecular mass. Also, the model presented in this section assumes the rod is rigid. In reality, DNA is not rigid, so a statistical theory of relaxation needs to be applied [247–249].

Takashima [98] and Sakamoto et al. [243] have derived a more comprehensive theory for counterion relaxation and found that the relaxation time varies in proportion to the square of the length of the molecule [249, 250]. Most experimental evidence indicates a *L*^2^ dependence. This is in contrast to the rigid-rod model where the relaxation time varies as *L*^3^.

### 19.5 Counterion Interaction With DNA and Proteins

The real and imaginary parts of permittivity depend on the concentration and type of cations [250]. As the concentration of the solvent increases, more of the phosphate charge is neutralized and the dielectric increment (difference between the permittivity of the mixture and solvent by itself) decreases.

Many types of cations compounds have been used in DNA solvents; for example, NaCl, LiCl, AgNO_2_, CuCl_2_, MnCl_2_, MgCl_2_, arginines, protamine, dyes, lysine, histones, and divalent metals such as Pb, Cd, Ni, Zn, and Hg [243, 251–253]. The simple inorganic-monovalent cations bind to the DNA molecule near the phosphate backbone to form both a condensed and diffuse sheath. There is evidence that strong concen trations of divalent metal cations destabilize the DNA helix [254]. Sakamoto et al. [252] found that the dielectric increment decreased for divalent cations.

On the other hand, histones and protamines tightly bind in the major groove of the DNA molecule. They produce stability in the double helix by neutralizing some of the phosphate charge. Dyes can attach to DNA, neutralize charge, and thereby decrease dielectric increment.

## 20. Methods for Modeling Electromagnetic Interactions With Biomolecules, Nanoprobes, and Nanowires

Modeling methods for EM interactions with materials include solving mode-match solutions to Maxwell’s equations, finite-element and molecular dynamics simulations, and finite-difference time-domain models. Finite-element modeling software can solve Maxwell’s equations for complicated geometries and small-scale systems.

Traditionally, mode-match solutions to Maxwell’s equations meant solving Maxwell’s equations in each region and then matching the modal field components at the interfaces and requiring, by the boundary conditions, all the tangential electric fields go to zero on conductors. On the nanoscale, the microwave and millimeter wavelengths are much larger than the feature size; the skin depths are usually larger than the device being measured. Therefore modes must be defined both outside the nanowire and inside the wire and matched at the interface. Also, the role of the near field is more important.

The EM model for a specific problem must capture the important physics such as skin depth, ballistic transport, conductor resistance, and quantized capacitance, without including all of the microstructural content. Modeling nanoscale electromagnetics is particularly difficult in that quantum effects cannot always be neglected; however, the EM field in these models is usually treated classically. In the case of near-field probes the skin depths are usually larger than the wire dimensions, and therefore the fields then need to be determined in both the wire and in the space surrounding the wire. Sommerfeld and Goubau surface waves and plasmons propagate at the interface of dielectric and finite conductivity metals and need to be taken into account in modeling probe interactions. The probe-material EM communication is often transmitted by the near field.

Recently, simulators for molecular dynamics have advanced to the stage where bonding, electrostatic interactions, and heat transfer can be modeled, and some now are beginning to include EM interactions.

## 21. Metrology Issues

### 21.1 Effects of Higher Modes in Transmission-Line Measurements

In this section we describe various common difficulties encountered in measurements of permittivity and permeability using transmission lines.

**Fig. 21:**
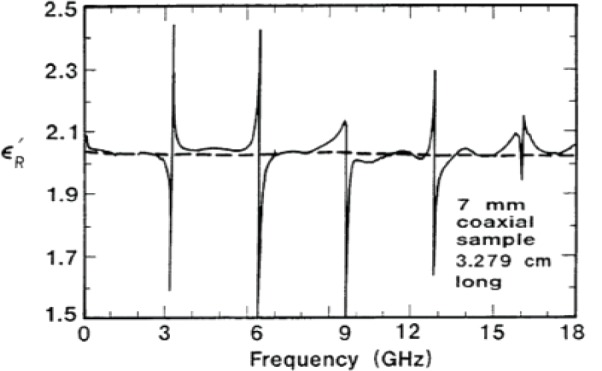
Permittivity calculation on a polytetrafluoroethylene (PTFE) material in a coaxial line that exhibits geometric resonance.

**Fig. 22:**
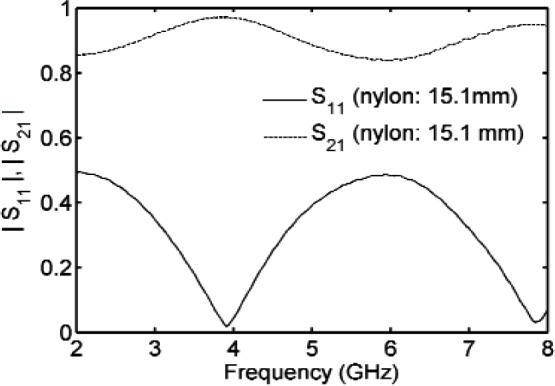
Scattering parameters |S11| and |S21| as a function of frequency for nylon in a coaxial line showing one-half wavelength standing waves.

**Fig. 24:**
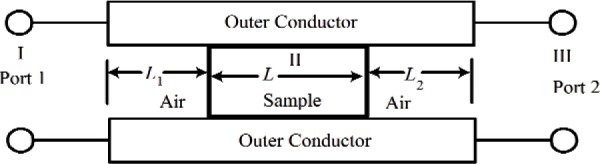
Cross-sectional view of a specimen in a coaxial line.

The definition of dielectric permittivity becomes blurred when the particle size in a material is no longer much smaller than a wavelength. To illustrate this problem, consider the permittivity from a transmission-line measurement of a PTFE specimen, which was reduced using the common Nicolson-Ross method [13] as shown in Fig. 21. Typical scattering parameters are shown in Fig. 22. The permittivity obtained from the scattering data is plotted as a function of frequency. The intrinsic relative permittivity is seen to be roughly 2.05, the commonly accepted value. However, when dimensional or Fabry-Perot resonances (see example in Fig. 22) across the sample occur at multiples of one-half wavelength, the specimen exhibits a geometrical standing-wave behavior at frequencies corresponding to *n*λ/2 across the sample. So if the sample is treated as a single particle at these standing wave frequencies, then the “effective” permittivity from this algorithm is no longer the intrinsic property of the material, but rather an artifact of geometric resonances across the sample. Geometrical resonances are sometimes used by metamaterial researchers to obtain effective negative permittivities and permeabilities that produce negative index response.

Homogeneous solid or liquid dielectric and magnetic materials have few intrinsic material resonances in the RF frequencies. The intrinsic resonances that do occur are primarily antiferromagnetic, ferromagnetic, water vapor and oxygen absorption bands, surface wave and plasma resonances, and atomic transitions. Dielectric resonances or standing waves that occur in solid and liquid dielectrics in RF frequencies are usually either a) geometric resonances of the fundamental mode across the specimen, b) an artifact of a higher mode that resonates across the length of the specimen, c) resonances or standing waves across the measurement fixture, or d) due to surface waves near interfaces between materials.

**Fig. 25:**
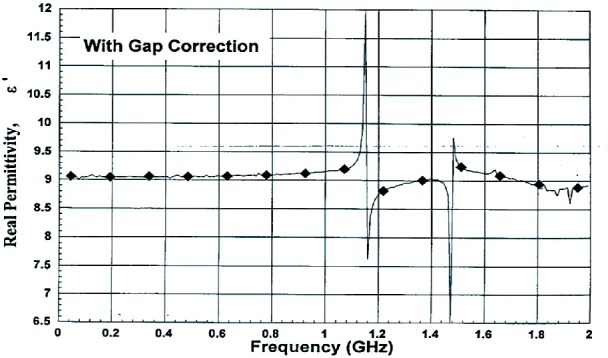
Higher non-TEM resonant modes in a coaxial fixture and anomalous behavior of the permittivity.

In the measurement of inhomogeneous materials in a transmission line or samples with a small air gap between the material and the fixture, higher modes may be produced and resonate across the specimen length in the measurement fixture. For example, in a coaxial line, the TE_0_*_n_* or TE_11_ mode may resonate across the specimen in a coaxial line measurement. These higher modes do not propagate in the air-filled waveguide since they are evanescent, but may propagate in the material-filled guide. Because these modes are not generally included in the field model, they produce a nonphysical geometric-based resonance in the reduced permittivity data, as shown in Fig. 25. These higher modes usually have low power and are caused by slight material or machining inhomogeneities. When these modes do propagate and resonate across the length of the specimen, it may appear as if the molecules in the material are under going intrinsic resonance, but this is not happening. In such cases, if the numerical model used for the data reduction uses only the fundamental mode, then the results obtained do not represent the permittivity of the material, but rather a related fixture specific geometric resonance of a higher mode (Fig. 25). These resonances are distinct from the fundamental-mode resonances obtained when the Nicolson-Ross-Weir reduction method is used [11] in transmission lines for materials at frequencies corresponding to *n*λ*_g_*/2, where *n* is an integer and λ*_g_* is the guided wavelength, as indicated in Fig. 21. The fundamental-mode resonances are modeled in the transmission-line theory and do not produce undue problems. However, in magnetic materials where there are both a permeability and permittivity, half wave geometric resonances and produce instabilities in the reduction algorithms [12].

### 21.2 Behavior of the Real Part of the Permittivity in Relaxation Response

For linear, homogeneous materials that are relaxing at RF frequencies, the permittivity decreases as frequency increases. The permittivity increases only near tails of intrinsic material resonances that only occur for frequencies in the high gigahertz region and above. To show this, we will analyze the prediction of the DRT permittivity model [90, 212].

We know that the behavior of the orientational polarization of most materials in time-dependent fields can, as a good approximation at low frequencies, be characterized with a distribution of relaxation times [53]. Typical numerical values of dielectric relaxation times in liquids are from 0.1 *μ*s to 1 ps.

We consider a description that has a distribution function y(τ), giving the probability distribution of relaxation times in the interval (τ,τ + *d*τ). The DRT model is summarized in Eq. (69). There are fundamental constraints on the distribution y(τ). It is non-negative everywhere, y(τ) ≥ 0 on τ ∈ [0,∞), and it is normalized,

(155) $$\int_{0}^{\infty }y(\tau )d\tau =1.$$

From Eq. (69) we have

(156) $$\frac{d\varepsilon^{\prime }(\omega )}{d\omega }=-2(\varepsilon_{s}-\varepsilon_{\infty })\omega \int_{0}^{\infty }\frac{\tau^{2}y(\tau )}{\left(1+\omega^{2}\tau^{2}\right)}d\tau <0.$$

This shows that ε′ is a decreasing function for all positive ω where the DRT model is valid (low RF frequencies), with a maximum only at ω = 0. The result of Eq. (156) holds for any distribution function y(τ). This model assumes there is only a relaxational response. If resonant behavior occurs at millimeter to terahertz frequencies, then the real part of the permittivity will show a slow increase as it approaches the resonance. In the regions of relaxation response, the real part of the permittivity is a decreasing function of frequency. Therefore, ε′(ω) attains a minimum at some frequency between relaxation and the beginning of resonance.

## 22. Permittivity Mixture Equations

We can readily estimate the permittivity of a mixture of a number of distinct materials. The effective permittivity of a mixture ε*_eff_* of constituents with permittivities ei and volume fractions θ*_i_* can be approximated in various ways. The Bruggeman equation [256] is useful for binary mixtures:

(157) $$\theta_{1}\frac{\varepsilon_{eff}^{\prime }-{\varepsilon^{\prime }}_{1}}{{\varepsilon^{\prime }}_{1}+2\varepsilon_{eff}^{\prime }}=\theta_{2}\frac{{\varepsilon^{\prime }}_{2}-\varepsilon_{eff}^{\prime }}{{\varepsilon^{\prime }}_{2}+2\varepsilon_{eff}^{\prime }},$$

or the Maxwell-Garnett mixture equation [256] can be used:

(158) $$\frac{\varepsilon_{eff}^{\prime }-{\varepsilon^{\prime }}_{2}}{\varepsilon_{eff}^{\prime }+2{\varepsilon^{\prime }}_{2}}=\theta_{1}\frac{{\varepsilon^{\prime }}_{1}-{\varepsilon^{\prime }}_{2}}{{\varepsilon^{\prime }}_{1}+2{\varepsilon^{\prime }}_{2}},$$

where ${\varepsilon^{\prime }}_{1}$ is the permittivity of the matrix and ${\varepsilon^{\prime }}_{2}$ is the permittivity of the filler [257]. The formula by Lichtenecker is for a powerlaw dependence of the real part of the permittivity for −1 ≤ *k* ≤ 1, and where the volume fractions of the inclusions and host are ν*_p_* and ν*_m_*:

(159) $$\varepsilon^{k}=\nu_{p}\varepsilon_{p}^{k}+\nu_{m}\varepsilon_{m}^{k}.$$

This equation has successfully modeled composites with random inclusions embedded into a host. An approximation to this is

(160) $$\ln\varepsilon =V_{p}\ln\varepsilon_{p}+V_{m}\ln\varepsilon_{m}.$$

## 23. Discussion

The broad area of RF dielectric electromagnetic interactions with solid and liquid materials from the macroscale down to the nanoscale materials was overviewed. The goal was to give a researcher a broad overview and access to references in the various areas. The paper studied the categories of electromagnetic fields, relaxation, resonance, susceptibility, linear response, interface phenomena, plasmons, the concepts of permittivity and permeability, and relaxation times. Topics of current research interest, such as plasmonic behavior, negative-index behavior, noise, heating, nanoscale materials, wave cloaking, polariton surface waves, biomaterials, and other topics were covered. The definition and limitations of the concept of permittivity in materials was discussed. We emphasized that the permittivity and permeability are well defined when the applied field has a wavelength much longer than the effective particle size in the material and when multiple scattering between inclusions is minimal as the wave propagates through the material. In addition, the use of the concept of permittivity requires an ensemble of particles that each have dielectric response.

## Figures and Tables

**Fig. 12:**
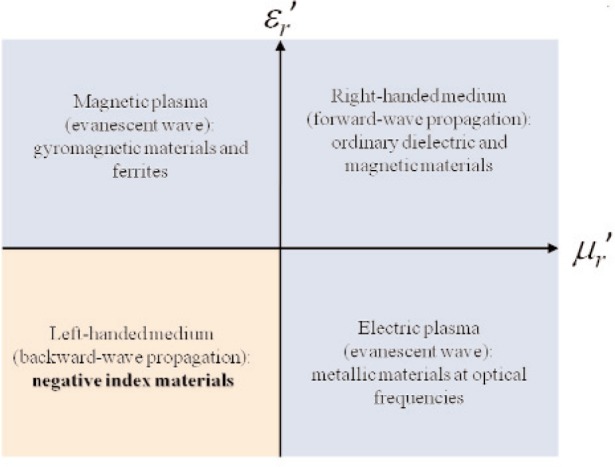
The regions of the permittivity-permeability space for different metamaterial behaviors.

**Fig. 14:**
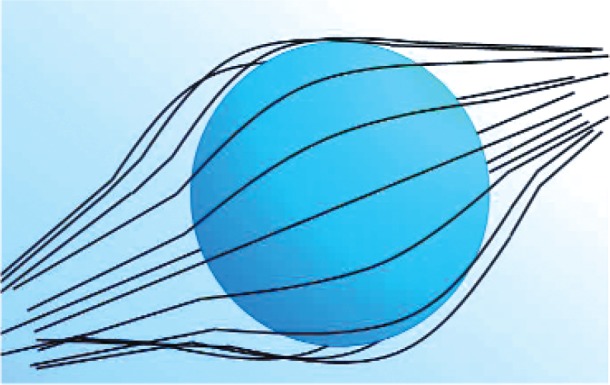
Cartoon of waves around a cloaked sphere.

**Fig. 23:**
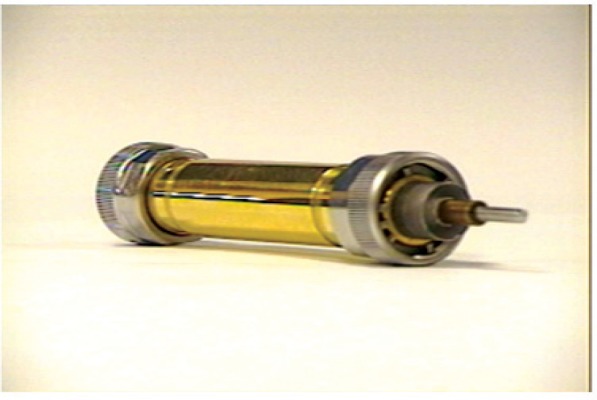
A typical coaxial line with a specimen inserted.

**Table 1:** Radio-Frequency Bands [1]

frequency	wavelength	band
3 – 30 kHz	100 – 10 km	VLF
30 – 300 kHz	10 – 1 km	LF
0.3 – 3 MHz	1 – 0.1 km	MF
3 – 30 MHz	100 – 10 m	HF
30 – 300 MHz	10 – 1 m	VHF
300 – 3000 MHz	100 – 10 cm	UHF
3 – 30 GHz	10 – 1 cm	SHF
30 – 300 GHz	10 – 1 mm	EHF
300 – 3000 GHz	1 – 0.1 mm	THz

**Table 2:** Relaxation Times of Common Liquids [105]

Material	ε*_s_*	ε_∞_	τ*_D_* (ps)	τ*_s_*(ps)
water (22 °C)	78.4	6.2	8.3	1.0
methanol (22 °C)	32.6	5.9	51.5	7.1
ethanol (22 °C)	24.3	4.5	163.0	9.0
1-propanol (22 °C)	20.4	3.7	329.0	15.0
2-propanol (22 °C)	19.4	2.4	59.0	..

**Table 3:** Heating Frequencies

Frequency (MHz)	Wavelength (cm)
13.56	2200
433.92	69
914	33
2450	12

**Table 4:** Radiation Classes and Approximate Photon Energies

Type	Frequency (Hz)	Photon energy (J)
γ-rays	3 × 10^20^	1.9 × 10^−13^
X-rays	3 × 10^16^	1.9 × 10^−14^
Ultraviolet	1 × 10^15^	6.4 × 10^−19^
Visible light	6 × 10^14^	4.0 × 10^−19^
Infrared light	3 × 10^12^	2.0 × 10^−22^
Microwave	2 × 10^9^	6.0 × 10^−25^
High frequency (HF)	1 × 10^6^	6.4 × 10^−28^

**Table 5:** Approximate dipole moments

Material	Dipole Moment (D)
H_2_O	1.85
CO	0.12
NaCl	9.00
Typical protein	500
Peptide	3.7
Amino acid	20
Polypeptide	1000
